# Global Epidemiologic Characteristics of Sexually Transmitted Infections Among Individuals Using Preexposure Prophylaxis for the Prevention of HIV Infection: A Systematic Review and Meta-analysis

**DOI:** 10.1001/jamanetworkopen.2019.17134

**Published:** 2019-12-11

**Authors:** Jason J. Ong, Rachel C. Baggaley, Teodora E. Wi, Joseph D. Tucker, Hongyun Fu, M. Kumi Smith, Sabrina Rafael, Vanessa Anglade, Jane Falconer, Richard Ofori-Asenso, Fern Terris-Prestholt, Ioannis Hodges-Mameletzis, Philippe Mayaud

**Affiliations:** 1 Department of Clinical Research, London School of Hygiene and Tropical Medicine, London, United Kingdom; 2 Central Clinical School, Monash University, Melbourne, Victoria, Australia; 3 Department of HIV, World Health Organization, Geneva, Switzerland; 4 Community Health and Research Division, Eastern Virginia Medical School, Norfolk; 5 Division of Epidemiology and Community Health, University of Minnesota Twin Cities, Minneapolis

## Abstract

**Importance:**

Despite a global increase in sexually transmitted infections (STIs), there is limited focus and investment in STI management within HIV programs, in which risks for STIs are likely to be elevated.

**Objective:**

To estimate the prevalence of STIs at initiation of HIV preexposure prophylaxis (PrEP; emtricitabine and tenofovir disoproxil fumarate) and the incidence of STIs during PrEP use.

**Data Sources:**

Nine databases were searched up to November 20, 2018, without language restrictions. The implementers of PrEP were also approached for additional unpublished data.

**Study Selection:**

Studies reporting STI prevalence and/or incidence among PrEP users were included.

**Data Extraction and Synthesis:**

Data were extracted independently by at least 2 reviewers. The methodological quality of studies was assessed using the Joanna Briggs Institute critical assessment tool for prevalence and incidence studies. Random-effects meta-analysis was performed.

**Main Outcomes and Measures:**

Pooled STI prevalence (ie, within 3 months of PrEP initiation) and STI incidence (ie, during PrEP use, after 3 months).

**Results:**

Of the 3325 articles identified, 88 were included (71 published and 17 unpublished). Data came from 26 countries; 62 studies (70%) were from high-income countries, and 58 studies (66%) were from programs only for men who have sex with men. In studies reporting a composite outcome of chlamydia, gonorrhea, and early syphilis, the pooled prevalence was 23.9% (95% CI, 18.6%-29.6%) before starting PrEP. The prevalence of the STI pathogen by anatomical site showed that prevalence was highest in the anorectum (chlamydia, 8.5% [95% CI, 6.3%-11.0%]; gonorrhea, 9.3% [95% CI, 4.7%-15.2%]) compared with genital sites (chlamydia, 4.0% [95% CI, 2.0%-6.6%]; gonorrhea, 2.1% [95% CI, 0.9%-3.7%]) and oropharyngeal sites (chlamydia, 2.4% [95% CI, 0.9%-4.5%]; gonorrhea, 4.9% [95% CI, 1.9%-9.1%]). The pooled incidence of studies reporting the composite outcome of chlamydia, gonorrhea, and early syphilis was 72.2 per 100 person-years (95% CI, 60.5-86.2 per 100 person-years).

**Conclusions and Relevance:**

Given the high burden of STIs among individuals initiating PrEP as well as persistent users of PrEP, this study highlights the need for active integration of HIV and STI services for an at-risk and underserved population.

## Key Points

**Question:**

What is the burden of sexually transmitted infections among individuals using preexposure prophylaxis (emtricitabine and tenofovir disoproxil fumarate) for the prevention of HIV infection?

**Findings:**

This systematic review and meta-analysis identified 88 studies (71 published and 17 unpublished), with 26 (30%) from low- and middle-income countries. For studies reporting a composite outcome of chlamydia, gonorrhea, and early syphilis, the pooled prevalence was 23.9% at initiation of HIV preexposure prophylaxis, and the pooled incidence was 72.2 per 100 person-years during HIV preexposure prophylaxis.

**Meaning:**

These estimates indicate a high burden of sexually transmitted infections among individuals initiating preexposure prophylaxis and persistent users of preexposure prophylaxis for the prevention of HIV infection, highlighting the opportunities for active integration of services for sexually transmitted infections and HIV preexposure prophylaxis.

## Teaser

This systematic review and meta-analysis estimates the prevalence of sexually transmitted infections at initiation of HIV preexposure prophylaxis (PrEP) and the incidence of sexually transmitted infections during use of HIV PrEP.

## Introduction

Preexposure prophylaxis (PrEP; emtricitabine and tenofovir disoproxil fumarate) for the prevention of HIV infection is safe and effective when there is a high level of adherence.^1,2,3,4^ The World Health Organization recommends the use of PrEP in subpopulations at substantial risk of HIV (ie, incidence >3 per 100 person-years).^5^ Operationally, this means that PrEP services are prioritized for men who have sex with men (MSM) in all world regions. Preexposure prophylaxis is also offered to the HIV-negative partner in HIV-serodiscordant partnerships as a bridge to viral suppression in several countries. In countries in East and Southern Africa with a high burden of HIV, PrEP services are provided for sex workers or for young women when the epidemiologic characteristics warrant.^6^ There is increasing interest and investment in implementing PrEP in low- and middle-income countries (LMICs) by large donors, such as the US President’s Emergency Plan for AIDS Relief and the Global Fund to Fight AIDS, Tuberculosis and Malaria. The Bill and Melinda Gates Foundation and Unitaid have also made substantial investments in PrEP in LMICs. However, recent estimates of the global burden of sexually transmitted infections (STIs)^7^ stress the need to consider programs that could address the synergistic epidemic of HIV and STIs.

Global guidelines dictate that PrEP programs focus on people at substantial risk for HIV, who are the same population at risk for other STIs. With growing interest in PrEP, more members of key populations are motivated to engage with health care systems than ever before. This change provides a unique opportunity to package PrEP services with more comprehensive sexual and reproductive health services at a moment of peak receptivity, particularly in LMICs where such services are currently limited. This plan is consistent with the World Health Organization Sustainable Development Goals to end the HIV epidemic and other communicable diseases, to improve sexual and reproductive health, and to achieve universal health coverage.^8^

In recent years, access to PrEP has shifted from provision in the context of demonstration projects to wider implementation through national health systems.^9^ To synthesize the latest available data to inform policies and practice around the provision of STI services within PrEP programs, we conducted a systematic review to estimate the prevalence and incidence of STIs among PrEP users. We supplemented data from the systematic review with data from key PrEP implementers who provided unpublished STI data. Previous systematic reviews have aimed to compare STI rates among PrEP users and nonusers, focused only on MSM, used data almost exclusively from high-income countries (HICs), and had limited search strategies.^10,11,12^ Since those reviews, an expanding body of PrEP studies from LMICs provides additional data. Unlike previous reviews, we aimed to describe the STI burden among PrEP users to highlight the potential lost opportunities if STI services are not provided for individuals initiating PrEP as well as persistent PrEP users. In particular, we contribute to the literature by providing pooled estimates according to anatomical site (ie, pharyngeal, genital, or anal site) that are valuable for informing STI testing recommendations and cost-effectiveness analyses.

## Methods

This review was conducted in 2 stages. First, a systematic review and meta-analysis was conducted in accordance with the Preferred Reporting Items for Systematic Reviews and Meta-analyses (PRISMA) checklist^13^ (PROSPERO registration: CRD42018116721). Second, a contact list of 82 PrEP implementers and/or researchers provided by the World Health Organization and some of us (J.J.O., J.D.T., F.T.-P., I.H.-M., and P.M.) was used. An email invitation to contribute unpublished STI data was sent to individuals on the contact list with a follow-up email 1 week later if there was no response. No financial incentives were offered for contributing the data.

We followed the guidelines in the *Cochrane Handbook for Systematic Reviews of Interventions*, version 5.1.^14^ The following 9 databases were searched from inception to November 20, 2018, without language restriction: Ovid MEDLINE (and In-Process and Other Nonindexed Citations and Daily), Ovid Embase, Ovid Global Health, Ovid EconLit, EBSCO CINAHL Plus, EBSCO Africa-Wide Information, Web of Science Core Collection, VHL LILACS, and Ovid Northern Light Life Sciences Conference Abstracts. The 2 key concepts anchoring our search strategy were STIs and PrEP (full details in eAppendix 1 in the Supplement). We included data from routine implementation programs (PrEP, prospective cohorts, randomized clinical trials, or demonstration projects of oral PrEP) that reported at least 1 of the following: frequency of STI testing and laboratory-confirmed STI positivity (incidence or prevalence). We included data from key STIs: *Chlamydia trachomatis*; *Neisseria gonorrhoeae*; *Treponema pallidum*; *Trichomonas vaginalis*; *Mycoplasma genitalium*; hepatitis A, B, and C; and herpes simplex virus. We excluded systematic reviews, letters, editorials, studies using only qualitative research methods, duplicated results from the same study, laboratory studies about testing STI diagnostic performance, and studies restricting study populations by clinical outcomes (eg, men with urethritis or women with cervicitis). We manually searched the references of existing systematic reviews^10,11,12^ to ensure our search strategy included all relevant articles. Once duplicates were removed, the titles and abstracts of articles were independently screened by at least 2 reviewers (M.K.S. and V.A.) according to a list of eligibility criteria; disagreements were discussed with 1 of us (J.J.O.). Data were reviewed by 1 of us (J.J.O.) for consistency and accuracy. Variables used for the data extraction are summarized in eAppendix 2 in the Supplement. We obtained missing data from articles of interest by contacting the corresponding authors. We emailed PrEP implementers to request data related to STI prevalence and/or incidence. Unpublished data were included if they fulfilled the same inclusion criteria, and at the time of request, these data have not yet been published or incorporated into existing publications.

### Statistical Analysis

Baseline prevalence was defined as STI diagnoses within 3 months of starting PrEP and confirmed by laboratory test results. Incidence was defined as STI diagnoses while the individual was taking PrEP and calculated as the number of new laboratory-confirmed STI cases divided by the total duration of exposure to PrEP, calculated as cases per 100 person-years. We extracted reported incidence rates and their 95% CIs when provided. If unavailable, we calculated the incidence by dividing the reported numbers of STI cases and time at risk, and we manually calculated the 95% CIs using the delta method to derive log rates and SEs. When time at risk was not available, we contacted authors for these data and excluded articles when we could not confidently measure STI prevalence or incidence.

Random-effects meta-analysis was used to calculate across-study pooled estimates of STI prevalence and STI incidence to account for sampling error and heterogeneity. Pooled estimates and 95% CIs were generated using a Freeman-Tukey–type double arcsine transformation to adjust for variance instability.^15^ Statistical heterogeneity between studies was assessed with the *I*^2^ statistic. Predefined subgroup meta-analyses were based on the following covariates: anatomical site (oropharyngeal, anorectal, or genital), study populations (MSM only or mixed [MSM and non-MSM]), type of study (observational or experimental), and country income level (HIC or LMIC). Observational studies include settings in which there may be additional user costs for STI testing (but could also be paid through a private insurance company, national health insurance, or from philanthropic groups) and thus may result in less systematic STI screening. Experimental studies follow a predefined study protocol for STI testing and thus may have more systematic STI screening. *High-income country* was defined as any country with a gross national income per capita of US $12 056 or more in 2017.^16^ Random-effects metaregression models were conducted to examine the association of these variables with the effect size. Funnel plots were generated to assess for the possibility of small-study effects that may be associated with publication bias. The Egger test was performed to confirm the presence of this bias.^17^ All analyses were conducted using Stata, version 13.1 (StataCorp LLC). We evaluated the methodological quality using the Joanna Briggs Institute critical assessment tool for prevalence and incidence studies.^18^ A score of 5 (out of 10) or above was deemed to be of sufficient quality to be included in the review.

## Results

**Figure 1.**
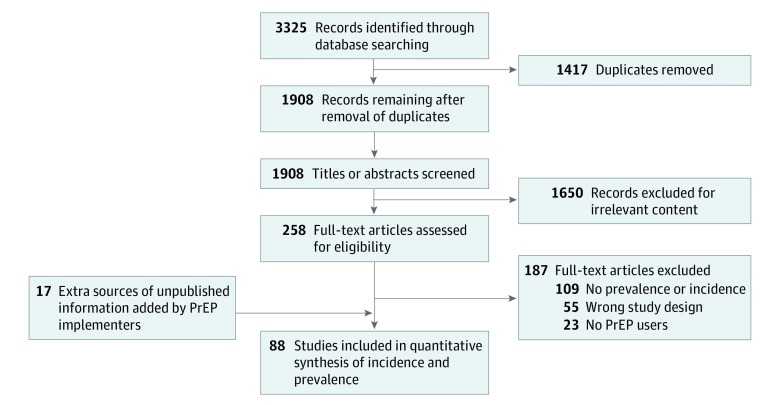
PRISMA Flowchart PrEP indicates preexposure prophylaxis.

**Figure 2.**
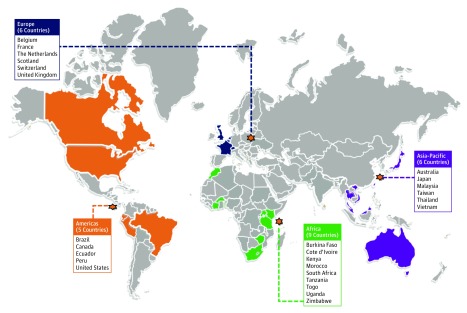
Countries That Provided Data for the Systematic Review

Of 3325 articles identified, 88 (71 published and 17 unpublished) met the inclusion criteria for prevalence and incidence data (Figure 1). Table 1 summarizes the characteristics of these studies: data came from 26 countries, mostly from HICs (62 [70%]) and from MSM-only programs (58 [66%]). Table 2 provides more data on included studies, all of which were deemed to be of sufficient methodological quality as determined by the Joanna Briggs Institute tool (ie, score of ≥5).^2,3,4,18,19,20,21,22,23,24,25,26,27,28,29,30,31,32,33,34,35,36,37,38,39,40,41,42,43,44,45,46,47,48,49,50,51,52,53,54,55,56,57,58,59,60,61,62,63,64,65,66,67,68,69,70,71,72,73,74,75,76,77,78,79,80,81,82,83,84,85,86^ A summary of the countries that provided data is shown in Figure 2.

**Table 1.** Characteristics of Reviewed Studies Reporting Sexually Transmitted Infection Prevalence or Incidence

Characteristic	Studies, No. (%) (N = 88)
Latest year of data	
Before 2013	9 (10)
2013-2015	25 (28)
2016-2018	50 (57)
Not available	4 (5)
Population	
MSM only	65 (74)
Mixed (ie, included non-MSM)^a^	23 (26)
Type of study	
Observational	73 (83)
Experimental	15 (17)
World Bank income level	
High income	62 (70)
Low or middle income	26 (30)

Abbreviation: MSM, men who have sex with men.

^a^ Non-MSM included serodiscordant couples, female sex workers, cisgender females, transgender individuals, and heterosexual individuals.

**Table 2.** Descriptive Characteristics of Included Studies and the Risk-of-Bias Assessment Using the Joanna Briggs Institute Tool

Source	Years of Data	Study Type	Country	Sample Size of PrEP Users, No.	MSM Only or Mixed Population, %^a^	Risk-of-Bias Assessment
Abrams-Downey et al,^19^ 2017	2013-2016	Observational	United States	599	MSM, 93; heterosexual, 7	8
Aloysius et al,^20^ 2017	2016-2017	Observational	United Kingdom	641	MSM	8
Anthony et al,^21^ 2016	2015-2016	Observational	United States	44	MSM, 89; female, 5	8
Vuylsteke et al,^22^ 2018	2015-2016	Observational	Belgium	200	MSM	8
Baeten et al,^23^ 2012	2008	Experimental	Kenya and Uganda	4758	MSM	7
Chaix et al,^24^ 2018	2014	Experimental	France and Canada	400	MSM	8
Beymer et al,^25^ 2018	2015-2016	Observational	United States	275	MSM	8
Bhatia et al,^26^ 2018	2012-2015	Observational	United States	40	MSM	8
Blaylock et al,^27^ 2018	2013-2016	Observational	United States	159	MSM, 63; female, 2; serodiscordant, 22; and young, 41	7
Bradshaw,^28^ 2018	2017-2018	Observational	United Kingdom	36	MSM	5
Bristow et al,^29^ 2018	Not available	Observational	United States	394	MSM	9
Celum et al,^30^ 2014	2008-2010	Experimental	Kenya and Uganda	1041	Serodiscordant, 100; female, 20	7
Chau and Goings,^31^ 2018	2017-2018	Observational	United States	1423	MSM, 93; female, 7	8
Cohen et al,^32^ 2015	2012-2013	Observational	United States	557	MSM	8
Cohen et al,^33^ 2016	2012-2014	Observational	United States	557	MSM	8
Coyer et al,^34^ 2018	2015-2017	Observational	The Netherlands	52	MSM	8
De Baetselier et al,^35^ 2018	2015-2016	Observational	Belgium	200	MSM	8
Delany-Moretlwe et al,^36^ 2018	2016-2017	Observational	South Africa and Tanzania	431	Female and young, 100	5
Elliott et al,^37^ 2018	2016-2017	Observational	United Kingdom	119	MSM	8
Freeborn et al,^38^ 2018	Not available	Observational	United States	81	MSM	5
Golub et al,^39^ 2018	Not available	Observational	United States	261	MSM	4
Grant et al,^40^ 2014	2011-2013	Observational	United States, Peru, Brazil, Thailand, South Africa, and Ecuador	1225	MSM	8
Grinsztejn et al,^41^ 2018	2014-2016	Observational	Brazil	375	MSM	6
Wu et al,^42^ 2018	2016-2017	Observational	Taiwan	302	MSM, 92; sex workers, 2; female, 4; heterosexual, 8	5
Hevey et al,^43^ 2018	2010-2016	Observational	United States	134	MSM, 96; heterosexual, 4	5
Hojilla,^44^ 2017	2014-2015	Observational	United States	268	MSM	5
Hoornenborg et al,^45^ 2018	2015	Observational	The Netherlands	330	MSM	5
Hosek et al,^46^ 2017	2013-2014	Observational	United States	78	MSM	8
Hosek et al,^47^ 2017	2013	Observational	United States	200	MSM	9
Irungu et al,^48^ 2016	2016	Observational	Kenya and Uganda	1694	Serodiscordant, 100	8
John et al,^49^ 2018	2015-2016	Observational	United States	104	MSM	8
Kenneth et al,^50^ 2016	2005-2015	Observational	United States	960	MSM, 76; young, 12	6
Kipyego et al,^51^ 2016	2008-2010	Observational	Kenya	967	Serodiscordant, 100	8
Knapper et al,^52^ 2018	2017	Observational	Wales	96	MSM	8
Cotte et al,^53^ 2018	2016-2017	Observational	France and Canada	162	MSM	7
Lal et al,^54^ 2017	2014-2015	Observational	Australia	114	MSM, 95; transgender, 1	8
Lalley-Chareczko et al,^55^ 2018	2015	Observational	United States	50	MSM	8
Liu et al,^56^ 2016	2014-2015	Observational	United States	437	MSM	8
La Fata et al,^57^ 2017	2016	Observational	France	202	MSM	6
Marcus et al,^58^ 2013	2007-2009	Experimental	Peru, Ecuador, South Africa, Brazil, Thailand, and United States	2205	MSM	9
Marcus et al,^59^ 2014	2007-2009	Experimental	Peru, Ecuador, South Africa, Brazil, Thailand, and United States	692	MSM	9
Marcus et al,^60^ 2016	2012-2014	Observational	United States	972	MSM	6
Mayer et al,^61^ 2017	2005-2015	Observational	United States	1631	MSM	8
McCormack and Dunn,^62^ 2015	2012-2014	Experimental	United Kingdom	545	MSM	9
McCormack et al,^3^ 2016	2012-2015	Experimental	United Kingdom	275	MSM	9
Molina et al,^4^ 2015	2012-2015	Experimental	France and Canada	199	MSM	8
Molina et al,^63^ 2018	2015-2016	Experimental	France	116	MSM	9
Molina et al,^64^ 2017	2014-2016	Observational	France and Canada	361	MSM	8
Nguyen et al,^65^ 2018	2010-2015	Observational	Canada	109	MSM	8
Nguyen et al,^66^ 2016	2015-2016	Observational	Canada	133	MSM	8
Noret et al,^67^ 2018	2015-2018	Observational	France	1049	MSM	8
Phanuphak et al,^68^ 2018	2016-2017	Observational	Thailand	1697	MSM	8
Hechter et al,^69^ 2018	2014-2016	Observational	United States	304	MSM	8
Reyniers et al,^70^ 2018	2015-2016	Observational	Belgium	200	MSM	9
Solomon et al,^71^ 2014	2007-2011	Experimental	Brazil, Peru, Ecuador, United States, South Africa, and Thailand	1251	MSM	9
Tabidze et al,^72^ 2018	2014-2016	Observational	United States	2981	MSM	7
Tiberio et al,^73^ 2016	2014-2015	Observational	United States	33	MSM, 82; young, 33; and heterosexual, 15	7
Tiraboschi et al,^74^ 2014	2013	Observational	United Kingdom	393	MSM	7
Traeger et al,^75^ 2018	2016-2018	Observational	Australia	2490	MSM	9
Volk et al,^76^ 2015	2012-2015	Observational	United States	657	MSM	6
Zablotska et al,^77^ 2015	2015	Observational	Australia	268	MSM	6
Grant et al,^2^ 2010	2007-2009	Experimental	Peru, Ecuador, South Africa, Brazil, Thailand, and United States	1251	MSM	9
Cotte et al,^78^ 2018	2016-2017	Observational	France	930	MSM	9
Hoornenborg et al,^79^ 2018	2015-2016	Observational	The Netherlands	376	MSM	7
Celum et al,^80^ 2019	2016-2018	Observational	South Africa and Zimbabwe	412	Female and young, 100	9
Hoornenborg et al,^81^ 2018	2015-2016	Observational	Amsterdam	376	MSM	9
Montaño et al,^82^ 2019	2014-2017	Observational	United States	183	MSM	7
Page et al,^83^ 2018	2016-2017	Observational	United States	170	MSM, 73; female, 17; and young, 19	7
Parsons et al,^84^ 2018	Not available	Observational	United States	281	MSM	7
Antonucci et al,^85^ 2014	2014	Experimental	United Kingdom	511	MSM	7
Volk et al,^86^ 2015	2011-2014	Observational	United States	485	MSM	5
Data direct from implementers						
Kimberley Green, PhD (written communication, January 2019)	2018	Observational	Vietnam	1221	Mixed	NA
Nittaya Phanuphak, PhD (written communication, December 2018)	2016-2017	Observational	Thailand	1697	Mixed	NA
Jennifer Morton, MPH (3P) (written communication, January 2019)	2017-2018	Observational	South Africa	200	Female, 100	NA
Jennifer Morton, MHP (POWER) (written communication, January 2019)	2017-2018	Observational	South Africa and Kenya	1255	Female, 100	NA
Pedro Carneiro, MPH (written communication, January 2019)	2015-2018	Observational	United States	13 685	MSM	NA
Andrew Grulich, PhD (EPIC-NSW) (written communication, December 2018)	2016-2018	Observational	Australia	8296	MSM	NA
Michalina Montaño, PhD (written communication, January 2019)	2014-2017	Observational	United States	365	MSM	NA
Iskandar Azwa, MRCP (written communication, January 2019)	2018-2019	Observational	Malaysia	-	MSM	NA
Daisuke Mizushima, PhD (written communication, January 2019)	2018	Observational	Japan	57	MSM	NA
Amal Ben Moussa, MD, and Mehdi Karkouri, MD (written communication, January 2019)	2018	Observational	Morocco	189	MSM, female sex workers	NA
Connie Celum, PhD (Voice) (written communication, March 2019)	2008	Experimental	South Africa, Uganda, and Zimbabwe	5029	Mixed	NA
Connie Celum, PhD (written communication, March 2019)	2008	Experimental	Kenya and Uganda	4758	Heterosexual and serodiscordant, 100	NA
Connie Celum, PhD (Plus pills) (written communication, March 2019)	2016	Observational	South Africa	150	Mixed	NA
de Baetselier, PhD (written communication, March 2019)	2018	Observational	Togo	103	MSM	NA
de Baetselier, PhD (written communication, March 2019)	2018	Observational	Cote D’Ivoire	100	MSM	NA
de Baetselier, PhD (written communication, March 2019)	2018	Observational	Burkina Faso	103	MSM	NA
Ellen White, MSc (PROUD) (written communication, February 2019)	2012-2016	Experimental	United Kingdom	275	MSM	NA

Abbreviations: 3P, PrEP-Power-Pride; EPIC-NSW, Expanded PrEP Implementation in Communities–New South Wales; MSM, men who have sex with men; NA, not applicable; Plus pills, Choices for Adolescent Prevention Methods for South Africa, Pilot Study B; POWER, Prevention Options for Women Evaluation Research; PrEP, preexposure prophylaxis; PROUD, Pre-exposure Option for Reducing HIV in the UK; Voice, Vagina and Oral Interventions to Control the Epidemic.

^a^ Mixed population may not add up to 100% as individuals may belong to more than 1 category or there are missing data. May include cisgender females, heterosexual individuals, transgender individuals, serodiscordant couples, female sex workers, or young people (<25 years of age).

### STI Prevalence and STI Incidence

Table 3 shows that, among studies reporting a composite outcome of any chlamydia, gonorrhea, and early syphilis, the pooled prevalence was 23.9% (95% CI, 18.6%-29.6%). The prevalence of chlamydia or gonorrhea by anatomical site was highest in the anorectum (chlamydia, 8.5% [95% CI, 6.3%-11.0%]; gonorrhea, 9.3% [95% CI, 4.7%-15.2%]) compared with genital sites (chlamydia, 4.0% [95% CI, 2.0%-6.6%]; gonorrhea, 2.1% [95% CI, 0.9%-3.7%]) and oropharyngeal sites (chlamydia, 2.4% [95% CI, 0.9%-4.5%]; gonorrhea, 4.9% [95% CI, 1.9%-9.1%]). The forest plots for the pooled prevalence by subgroups are provided in eAppendix 3 in the Supplement. For example, the prevalence of chlamydia differed by study population (MSM, 6.9% [95% CI, 5.4%-8.6%]; mixed, 10.7% [95% CI, 0%-38.0%]), study type (observational, 7.9% [95% CI, 5.6%-10.4%]; experimental, 3.1% [95% CI, 1.1%-6.1%]), and country income level (HIC, 7.5% [95% CI, 5.7%-9.6%]; LMIC, 6.6% [95% CI, 2.2%-12.8%]).

**Table 3.** Pooled Prevalence of STIs When Starting PrEP and Pooled Incidence of STIs, by Anatomical Site of Detection

Pathogen	Prevalence					Incidence				
	No. of Studies Pooled	Total Sample Size, No.	Prevalence (95% CI)	*I*^2^ Statistic, %	*P* Value	No. of Studies Pooled	Total Sample Size, No.	Incidence per 100 Person-Years (95% CI)	*I*^2^ Statistic, %	*P* Value
*Chlamydia trachomatis*										
Any site	12	4918	10.8 (6.4-16.1)	97	<.001	14	6756	21.5 (17.9-25.8)	97	<.001
Genital	6	1019	4.0 (2.0-6.6)	66	.01	9	1698	10.4 (9.2-11.8)	0	.78
Anorectal	8	1660	8.5 (6.3-11.0)	61	.01	11	2171	29.9 (24.1-37.1)	87	<.001
Oropharyngeal	5	939	2.4 (0.9-4.5)	63	.03	7	1237	4.6 (3.3-6.3)	46	.10
*Neisseria gonorrhoeae*										
Any site	14	6340	11.6 (7.6-16.2)	96	<.001	13	6462	37.1 (18.3-25.5)	96	<.001
Genital	6	2166	2.1 (0.9-3.7)	70	.01	8	1564	9.9 (8.3-11.8)	28	.20
Anorectal	8	1558	9.3 (4.7-15.2)	92	<.001	11	2171	21.6 (16.4-28.4)	90	<.001
Oropharyngeal	5	940	4.9 (1.9-9.1)	83	<.001	8	1646	19.7 (16.0-24.3)	76	<.001
*Treponema pallidum* ^a^	22	9757	5.0 (3.1-7.4)	95	<.001	23	12 459	11.6 (9.2-14.6)	92	<.001
Hepatitis A virus	1	1049	5.4 (4.1-7.0)	NA	NA	NA	NA	NA	NA	NA
Hepatitis B virus	4	4370	1.3 (0.1-3.5)	95	<.001	2	1353	1.2 (0.6-2.6)	0	.53
Hepatitis C virus	4	2555	2.0 (0.8-3.7)	84	<.001	8	3786	0.3 (0.1-0.9)	87	<.001
*Mycoplasma genitalium*	1	198	17.2 (12.2-23.2)	NA	NA	NA	NA	NA	NA	NA
*Trichomonas vaginalis*	2	1379	5.9 (4.7-7.2)	NA	NA	1	50	0	NA	NA
Any *C trachomatis*, *N gonorrhoeae*, or *T pallidum*	16	8431	23.9 (18.6-29.6)	97	<.001	11	6301	72.2 (60.5-86.2)	95	<.001

Abbreviations: NA, not applicable; PrEP, preexposure prophylaxis; STI, sexually transmitted infection.

^a^ Early syphilis, primary or secondary syphilis, or early latent syphilis.

In studies that reported a composite outcome of any chlamydia, gonorrhea, and early syphilis, the pooled incidence was 72.2 per 100 person-years (95% CI, 60.5-86.2 per 100 person-years). The incidence of chlamydia or gonorrhea by anatomical site was highest in the anorectum (chlamydia, 29.9 per 100 person-years [95% CI, 24.1-37.1 per 100 person-years]; gonorrhea, 21.6 per 100 person-years [95% CI, 16.4-28.4 per 100 person-years]) compared with genital sites (chlamydia, 10.4 per 100 person-years [95% CI, 9.2-11.8 per 100 person-years]; gonorrhea, 9.9 per 100 person-years [95% CI, 8.3-11.8 per 100 person-years]) and oropharyngeal sites (chlamydia, 4.6 per 100 person-years [95% CI, 3.3-6.3 per 100 person-years]; gonorrhea, 19.7 per 100 person-years [95% CI, 16.0-24.3 per 100 person-years]). Compared with oropharyngeal chlamydia, the reported incidence of oropharyngeal gonorrhea was significantly higher. The forest plots for the pooled incidence by subgroup are provided in eFigures 1 to 11 in the Supplement (eAppendix 3 in the Supplement). The incidence of chlamydia differed by study type (observational, 22.4 per 100 person-years [95% CI, 18.6-27.0 per 100 person-years]; experimental, 17.0 per 100 person-years [95% CI, 8.7-33.3 per 100 person-years]) and country income level (HIC, 22.1 per 100 person-years [95% CI, 18.5-26.5 per 100 person-years]; LMIC, 8 per 100 person-years [95% CI, 5.6-11.5 per 100 person-years]).

A few observations from the metaregression results are notable (eTables 1-7 in the Supplement). The prevalence of gonorrhea was higher in studies that enrolled MSM only (adjusted odds ratio [AOR], 1.11 [95% CI, 1.00-1.22]) compared with studies also containing non-MSM populations (eTable 2 in the Supplement). The incidence of chlamydia was higher in the anorectum (AOR, 7.25 [95% CI, 4.83-10.86]) and genital sites (AOR, 2.20 [95% CI, 4.83-10.86]) than in oropharyngeal sites, and it was higher in HICs (AOR, 4.92 [95% CI, 2.35-10.32]) than in LMICs (eTable 5 in the Supplement). Visual inspection of the funnel plots and the Egger test found an indication of small-study effects, with underestimation of the true chlamydia incidence rate (eFigure 7 in the Supplement). The incidence of gonorrhea was lower in genital sites than in oropharyngeal sites (AOR, 0.50 [95% CI, 0.32-0.77]), and it was higher in HICs than in LMICs (AOR, 7.03 [95% CI, 2.62-18.88]; eTable 6 in the Supplement). Visual inspection of the funnel plots and the Egger test found an indication of small-study effects, with underestimation of the true gonorrhea incidence rate (eFigure 6 in the Supplement). The incidence of early syphilis was higher in HICs (AOR, 3.93 [95% CI, 1.36-11.41]) than in LMICs (eTable 7 in the Supplement). Visual inspection of the funnel plots and the Egger test found an indication of small-study effects, with underestimation of the true early hepatitis C incidence rate (eFigure 11 in the Supplement).

## Discussion

This systematic review and meta-analysis consolidates the published and unpublished evidence of the high STI burden among individuals initiating PrEP as well as among persistent PrEP users. Our findings underscore the lost opportunities if STI services are not provided for individuals initiating PrEP and highlights the opportunity to harness the growing interest in providing PrEP programs globally to be a gateway to provide more comprehensive sexual and reproductive health services for PrEP users. There are opportunities for economies of scope and scale to control STIs by leveraging the growing infrastructure of PrEP delivery and access to higher-risk individuals. Synergistically, the identification of high-risk individuals with STIs can be a gateway for the provision of PrEP. Implementing more frequent STI screening and testing and partner services among high-risk individuals may potentially lessen the effect of STI epidemics.^87,88^ As we strengthen the delivery of sexual and reproductive health services for PrEP users globally, there may also be a positive flow-on effect for nonusers living with HIV who also are at high risk for STIs, and other nonusers may also be able to access these services.

The high pooled prevalence of STIs among those starting PrEP reinforces the belief that we are reaching groups at high risk for HIV and STIs, and the high pooled incidence emphasizes the need for ongoing STI testing and treatment services because PrEP users remain at high risk for STIs. Our study complements other meta-analyses of STI incidence among MSM only^10,11,12^; however, we extend their findings by examining sources of heterogeneity according to anatomical site of detection, study population composition, country income level, and study type. We noted a high level of heterogeneity in our pooled estimates, which may be due to additional factors, including differences in background HIV prevalence in country or setting, case mix of populations (ie, sampling different underlying populations: different distributions of socioeconomic status, race/ethnicity, age, or sexual mixing networks), study designs (variable inclusion criteria for PrEP, different frequency of testing), and STI diagnostic protocols (eg, the Pre-exposure Prophylaxis Initiative [iPrEx] trial^2,40^ analyzed urethral samples for chlamydia or gonorrhea only if leucocytes were present in urine, whereas Australian demonstration projects^75^ did not impose such reliance on urine leucocytes). Nevertheless, despite this high level of heterogeneity between studies, the consistently high STI prevalence and incidence reported in individual studies cannot be ignored.

This systematic review uncovered several important gaps in evidence. First, we found only 1 article that reported antimicrobial-resistant *M genitalium* among PrEP users.^35^ With expected high yields of positive samples from PrEP users, PrEP programs may be useful as sentinel surveillance sites for STI–antimicrobial resistance monitoring for *N gonorrhoeae* and *M genitalium*. Second, there are inconsistencies in how STI prevalence and STI incidence are reported, precluding their inclusion in meta-analyses. For future meta-analyses, reporting the number of cases with person-years at risk or incidence rates with 95% CIs would be a minimum requirement. We recommend disaggregating STI prevalence and STI incidence by pathogen and subpopulations (eg, age, sex, or transgender identity).

### Policy Implications

Our study is useful to advocate for improved access to STI services for PrEP users and to inform program design and cost-effectiveness analyses. There is a clear need to facilitate the development of affordable, accurate, and easy-to-use point-of-care tests for STIs and developing models for STI case management in resource-constrained settings. A reevaluation is needed of how diagnostic costs can be reduced and how economies of scope and scale may be gained from using the existing infrastructure of cartridge-based molecular diagnostic machines that are used for other diseases (such as tuberculosis). The current interest, demand, and support for PrEP services in LMICs is predicated on a need to provide PrEP as simply and cheaply as possible. Therefore, a tension exists between the increasing costs and complexity of PrEP implementation and the opportunity and need to provide effective STI services. A market and technology landscape report for STI diagnostics (similar to HIV self-testing^89^) would be a helpful resource for PrEP programs. Furthermore, guidance from international authorities, such as the World Health Organization, will be needed to define what may be considered as essential sexual health services compared with enhanced services, particularly in resource-constrained settings.

There are ongoing challenges in implementing integrated STI services within PrEP programs. The key challenges are related to STI diagnostics, program logistics of combined STI and PrEP delivery, and lack of STI capacity building. Particularly for LMICs, there is a lack of access to triple–anatomical site sampling (ie, testing from oropharyngeal, urogenital, and anorectal sites), which is critical for detecting STIs in MSM.^90^ This situation is usually related to lack of funding, so considerations should be given to the burgeoning evidence for pooled samples testing.^91^ A robust economic case is pertinent because cost has been raised as a major barrier, even in HICs where direct user costs may be incurred by those with no health insurance.

### Strengths and Limitations

The strength of our review is the inclusion of data from 26 countries including non-MSM populations, LMIC settings, and previously unreported STI data. Our findings should be considered in light of several limitations. First, there is a potential for selection and detection bias. The high STI prevalence for individuals starting PrEP may reflect the inclusion criteria for some PrEP programs (ie, some clinicians may encourage same-day referral for PrEP when a rectal STI is diagnosed). The pooled incidence may be overestimated owing to more frequent testing and from more anatomical sites. Second, not all PrEP-related publications focused on reporting STI data. We mitigated this factor by approaching PrEP programs for unpublished STI data. Third, we included only laboratory-confirmed STIs. Therefore, most estimates came from HICs where diagnostics were available, whereas estimates obtained in LMICs are representative of externally funded research programs.

## Conclusions

Given the high STI burden among individuals initiating PrEP and among persistent PrEP users, there are opportunities to leverage the global interest in PrEP policy and the development of programs to actively promote the integration of STI services, which includes appropriate asymptomatic testing, treatment, and targeted vaccination. Currently, fewer STI data are available from programs offering PrEP to women, young people, serodiscordant couples, and transgender individuals outside HICs. More data would help guide recommendations on the frequency and optimal STI testing approaches for all population groups accessing PrEP.

## Notes

Supplement. **eAppendix 1.** Search Methodology **eAppendix 2.** Data Variables Used in Data Extraction **eAppendix 3.** Forest Plots by Pathogen and Subgroups **eFigure 1.** Random Effects Meta-Analysis of Chlamydia Prevalence **eTable 1.** Meta-Regression Results for the Predictors of Chlamydia Prevalence and Sources of Between-Study Heterogeneity **eFigure 2.** Random Effects Meta-Analysis of Gonorrhea Prevalence **eTable 2.** Meta-Regression Results for the Predictors of Gonorrhea Prevalence and Sources of Between-Study Heterogeneity **eFigure 3.** Random Effects Meta-Analysis of Early Syphilis Prevalence **eTable 3.** Meta-Regression Results for the Predictors of Early Syphilis Prevalence and Sources of Between-Study Heterogeneity **eFigure 4.** Random Effects Meta-Analysis of Any Chlamydia, Gonorrhea, or Early Syphilis Prevalence **eTable 4.** Meta-Regression Results for the Predictors of Any Chlamydia, Gonorrhea, or Early Syphilis Prevalence and Sources of Between-Study Heterogeneity **eFigure 5.** Random Effects Meta-Analysis of Hepatitis B Prevalence **eFigure 6.** Random Effects Meta-Analysis of Hepatitis C Prevalence **eFigure 7.** Random Effects Meta-Analysis of Chlamydia Incidence **eTable 5.** Meta-Regression Results for the Predictors of Chlamydia Incidence and Sources of Between-Study Heterogeneity **eFigure 8.** Random Effects Meta-Analysis of Gonorrhea Incidence **eTable 6.** Meta-Regression Results for the Predictors of Gonorrhea Incidence and Sources of Between-Study Heterogeneity **eFigure 9.** Random Effects Meta-Analysis of Early Syphilis Incidence **eTable 7.** Meta-Regression Results for the Predictors of Early Syphilis Incidence and Sources of Between-Study Heterogeneity **eFigure 10.** Random Effects Meta-Analysis of Any Chlamydia, Gonorrhea or Early Syphilis Incidence **eFigure 11.** Random Effects Meta-Analysis of Hepatitis C Incidence

## References

[1] Cohen MS, Chen YQ, McCauley M, ; HPTN 052 Study Team Prevention of HIV-1 infection with early antiretroviral therapy. N Engl J Med. 2011;365(6):-. doi:10.1056/NEJMoa1105243 21767103 PMC3200068

[2] Grant RM, Lama JR, Anderson PL, ; iPrEx Study Team Preexposure chemoprophylaxis for HIV prevention in men who have sex with men. N Engl J Med. 2010;363(27):2587-2599. doi:10.1056/NEJMoa1011205 21091279 PMC3079639

[3] McCormack S, Dunn DT, Desai M, Pre-exposure prophylaxis to prevent the acquisition of HIV-1 infection (PROUD): effectiveness results from the pilot phase of a pragmatic open-label randomised trial. Lancet. 2016;387(10013):53-60. doi:10.1016/S0140-6736(15)00056-2 26364263 PMC4700047

[4] Molina JM, Capitant C, Spire B, ; ANRS IPERGAY Study Group On-demand preexposure prophylaxis in men at high risk for HIV-1 infection. N Engl J Med. 2015;373(23):2237-2246. doi:10.1056/NEJMoa1506273 26624850

[5] World Health Organization WHO expands recommendation on oral pre-exposure prophylaxis of HIV infection (PrEP): policy brief. https://www.who.int/hiv/pub/prep/policy-brief-prep-2015/en/. Published November 2015. Accessed April 1, 2019.

[6] Hodges-Mameletzis I, Dalal S, Msimanga-Radebe B, Rodolph M, Baggaley R Going global: the adoption of the World Health Organization’s enabling recommendation on oral pre-exposure prophylaxis for HIV. Sex Health. 2018;15(6):489-500. doi:10.1071/SH18125 30496718

[7] Rowley J, Vander Hoorn S, Korenromp E, Chlamydia, gonorrhoea, trichomoniasis and syphilis: global prevalence and incidence estimates, 2016. Bull World Health Organ. 2019;97(8):548-562P. doi:10.2471/BLT.18.228486 31384073 PMC6653813

[8] World Health Organization Sexual health and its linkages to reproductive health: an operational approach. https://www.who.int/reproductivehealth/publications/sexual_health/sh-linkages-rh/en/. Published 2017. Accessed May 2, 2019.

[9] Global Advocacy for HIV Prevention PrEPWatch. https://www.prepwatch.org/. Accessed April 10, 2019.

[10] Kojima N, Davey DJ, Klausner JD Pre-exposure prophylaxis for HIV infection and new sexually transmitted infections among men who have sex with men. AIDS. 2016;30(14):2251-2252. doi:10.1097/QAD.0000000000001185 27314179

[11] Traeger MW, Schroeder SE, Wright EJ, Effects of pre-exposure prophylaxis for the prevention of human immunodeficiency virus infection on sexual risk behavior in men who have sex with men: a systematic review and meta-analysis. Clin Infect Dis. 2018;67(5):676-686. doi:10.1093/cid/ciy182 29509889

[12] Werner RN, Gaskins M, Nast A, Dressler C Incidence of sexually transmitted infections in men who have sex with men and who are at substantial risk of HIV infection—a meta-analysis of data from trials and observational studies of HIV pre-exposure prophylaxis. PLoS One. 2018;13(12):e0208107. doi:10.1371/journal.pone.0208107 30507962 PMC6277101

[13] PRISMA Preferred Reporting Items for Systematic Reviews and Meta-analyses. http://www.prisma-statement.org/. Accessed April 5, 2019.

[14] Higgins JPT, Green S, eds. Cochrane Training. *Cochrane Handbook for Systematic Reviews of Interventions* Version 5.1. https://training.cochrane.org/handbook. The Cochrane Collaboration. Updated March 2011. Accessed April 5, 2019.

[15] Barendregt JJ, Doi SA, Lee YY, Norman RE, Vos T Meta-analysis of prevalence. J Epidemiol Community Health. 2013;67(11):974-978. doi:10.1136/jech-2013-203104 23963506

[16] The World Bank World Bank country and lending groups. https://datahelpdesk.worldbank.org/knowledgebase/articles/906519-world-bank-country-and-lending-groups. Accessed May 2, 2019.

[17] Egger M, Davey Smith G, Schneider M, Minder C Bias in meta-analysis detected by a simple, graphical test. BMJ. 1997;315(7109):629-634. doi:10.1136/bmj.315.7109.629 9310563 PMC2127453

[18] Joanna Briggs Institute. Critical appraisal tools. https://www.joannabriggs.org/research/critical-appraisal-tools.html. Accessed April 2, 2019.

[19] Abrams-Downey A, Ventuneac A, Duah B, Risk factors associated with sexually transmitted infections among pre-exposure prophylaxis users in an urban multi-clinic healthcare system. Open Forum Infect Dis. 2017;4(suppl_1):S668-S669. doi:10.1093/ofid/ofx163.1785

[20] Aloysius I, Savage A, Zdravkov J, InterPrEP: internet-based pre-exposure prophylaxis with generic tenofovir DF/emtricitabine in London: an analysis of outcomes in 641 patients. J Virus Erad. 2017;3(4):218-222. 29057086 10.1016/S2055-6640(20)30317-4 PMC5632549

[21] Anthony K, Asbel L, Thompson F, Madera RT Integrating PrEP for HIV in a categorical STD clinic within a high risk urban setting. Sex Transm Dis. 2016;43(10)(suppl 2):S132.

[22] Vuylsteke B, Reyniers T, Baetselier ID, Daily or event-driven PrEP? interim results of “Be-PrEP-Ared,” a PrEP demonstration project among men who have sex with men in Belgium. Paper presented at: 22nd International AIDS Conference; July 23-27, 2018; Amsterdam, the Netherlands.

[23] Baeten JM, Donnell D, Ndase P, ; Partners PrEP Study Team Antiretroviral prophylaxis for HIV prevention in heterosexual men and women. N Engl J Med. 2012;367(5):399-410. doi:10.1056/NEJMoa1108524 22784037 PMC3770474

[24] Chaix ML, Charreau I, Pintado C, ; ANRS IPERGAY Study Group Effect of on-demand oral pre-exposure prophylaxis with tenofovir/emtricitabine on herpes simplex virus-1/2 incidence among men who have sex with men: a substudy of the ANRS IPERGAY trial. Open Forum Infect Dis. 2018;5(11):ofy295. doi:10.1093/ofid/ofy295 30539039 PMC6286447

[25] Beymer MR, DeVost MA, Weiss RE, Does HIV pre-exposure prophylaxis use lead to a higher incidence of sexually transmitted infections? a case-crossover study of men who have sex with men in Los Angeles, California. Sex Transm Infect. 2018;94(6):457-462. doi:10.1136/sextrans-2017-053377 29487172 PMC6482844

[26] Bhatia R, Modali L, Lowther M, Outcomes of preexposure prophylaxis referrals from public STI clinics and implications for the preexposure prophylaxis continuum. Sex Transm Dis. 2018;45(1):50-55. doi:10.1097/OLQ.0000000000000690 28876282

[27] Blaylock JM, Hakre S, Decker CF, HIV PrEP in the military: experience at a tertiary care military medical center. Mil Med. 2018;183(3/4)(suppl 1):445-449. doi:10.1093/milmed/usx143 29635556

[28] Bradshaw H Pre-exposure prophylaxis (PrEP) with tenofovir and emtricitabine in clinical practice and the issues involved. HIV Med. 2018;19(suppl 2):S57.

[29] Bristow C, Moore DJ, Dube M, Sexually transmitted infections and adherence to PrEPns. Top Antivir Med. 2018;26(suppl 1):469-470.

[30] Celum C, Morrow RA, Donnell D, Daily oral tenofovir and emtricitabine-tenofovir preexposure prophylaxis reduces herpes simplex virus type 2 acquisition among heterosexual HIV-1–uninfected men and women: a subgroup analysis of a randomized trial [published correction appears in *Ann Intern Med*. 2016;165(11):832]. Ann Intern Med. 2014;161(1):11-19. doi:10.7326/L16-0549 24979446

[31] Chau D, Goings S STI rates among PrEP users within a sexual health clinic in Austin, TX. Sex Transm Dis. 2018;45(suppl 2):S111.

[32] Cohen SE, Vittinghoff E, Bacon O, High interest in preexposure prophylaxis among men who have sex with men at risk for HIV infection: baseline data from the US PrEP demonstration project. J Acquir Immune Defic Syndr. 2015;68(4):439-448. doi:10.1097/QAI.0000000000000479 25501614 PMC4334721

[33] Cohen SE, Vittinghoff E, Philip SS, Elion R, Kolber MA, Liu AY Repeat rectal gonorrhea and chlamydia infections in a cohort of participants on PrEP. Sex Transm Dis. 2016;43(10)(suppl 2):S177.

[34] Coyer L, van Bilsen W, Bil J, Pre-exposure prophylaxis among men who have sex with men in the Amsterdam Cohort Studies: use, eligibility, and intention to use. PLoS One. 2018;13(10):e0205663. doi:10.1371/journal.pone.0205663 30312336 PMC6185853

[35] De Baetselier I, Smet H, Wouters K, High level of macrolide resistance of *Mycoplasma genitalium* found among MSM at high risk for HIV in a Belgian PrEP demonstration project. AIDS Res Hum Retroviruses. 2018;34(suppl 1):291.

[36] Delany-Moretlwe S, Chersich M, Harvey S, Empowerment clubs did not increase PrEP continuation among adolescent girls and young women in South Africa and Tanzania—results from the EMPOWER randomised trial. J Int AIDS Soc. 2018;21(suppl 6):169.

[37] Elliott T, Hawkins L, Proserpio M, Dosekun O Experience of a PrEP service in a central London sexual health clinic. HIV Med. 2018;19(suppl 2):S49.

[38] Freeborn K, Portillo C, Boyer CB, Santos GM Misclassification of sexual health risks in a self-identified low risk cohort of men who have sex with men (MSM) enrolled in a community based PrEP program [published online May 25, 2019]. AIDS Care. doi:10.1080/09540121.2019.1620167 PMC6875612 31129982

[39] Golub SA, Pena S, Fikslin RA, Goldberg M, Radix A Partners, not condom use, drive STI rates among PrEP users in community health center. Top Antivir Med. 2018;26(suppl 1):468.

[40] Grant RM, Anderson PL, McMahan V, ; iPrEx Study Team Uptake of pre-exposure prophylaxis, sexual practices, and HIV incidence in men and transgender women who have sex with men: a cohort study. Lancet Infect Dis. 2014;14(9):820-829. doi:10.1016/S1473-3099(14)70847-3 25065857 PMC6107918

[41] Grinsztejn B, Hoagland B, Moreira RI, ; PrEP Brasil Study Team Retention, engagement, and adherence to pre-exposure prophylaxis for men who have sex with men and transgender women in PrEP Brasil: 48 week results of a demonstration study. Lancet HIV. 2018;5(3):e136-e145. doi:10.1016/S2352-3018(18)30008-0 29467098

[42] Wu HJ, Strong C, Ku SWW, Syphilis acquisition and dosing schedule for pre-exposure prophylaxis (PrEP) users in Taiwan PrEP demonstration project. Paper presented at: 22nd International AIDS Conference July 23-27, 2018; Amsterdam, the Netherlands.

[43] Hevey MA, Walsh JL, Petroll AE PrEP continuation, HIV and STI testing rates, and delivery of preventive care in a clinic-based cohort. AIDS Educ Prev. 2018;30(5):393-405. doi:10.1521/aeap.2018.30.5.393 30332309 PMC6535209

[44] Hojilla JC Optimizing the Delivery of HIV Pre-Exposure Prophylaxis (PrEP): An Evaluation of Risk Compensation, Disengagement, and the PrEP Cascade. San Francisco: School of Nursing, University of California; 2017.

[45] Hoornenborg E, Coyer L, van Laarhoven A, ; Amsterdam PrEP Project Team in the HIV Transmission Elimination Amsterdam Initiative Change in sexual risk behaviour after 6 months of pre-exposure prophylaxis use: results from the Amsterdam pre-exposure prophylaxis demonstration project. AIDS. 2018;32(11):1527-1532. doi:10.1097/QAD.0000000000001874 29762169

[46] Hosek SG, Landovitz RJ, Kapogiannis B, Safety and feasibility of antiretroviral preexposure prophylaxis for adolescent men who have sex with men aged 15 to 17 years in the United States. JAMA Pediatr. 2017;171(11):1063-1071. doi:10.1001/jamapediatrics.2017.2007 28873128 PMC5710370

[47] Hosek SG, Rudy B, Landovitz R, ; Adolescent Trials Network (ATN) for HIVAIDS Interventions An HIV preexposure prophylaxis demonstration project and safety study for young MSM. J Acquir Immune Defic Syndr. 2017;74(1):21-29. doi:10.1097/QAI.0000000000001179 27632233 PMC5140725

[48] Irungu E, Heffron R, Ngure K, Baeten J, Mugo N Unmet need for PrEP among HBV-infected/HIV-uninfected partners in HIV serodiscordant partnerships. AIDS Res Hum Retroviruses. 2016;32(suppl 1):365.

[49] John SA, Parsons JT, Rendina HJ, Grov C Club drug users had higher odds of reporting a bacterial STI compared with non-club drug users: results from a cross-sectional analysis of gay and bisexual men on HIV pre-exposure prophylaxis. [published online August 20, 2018]. Sex Transm Infect. doi:10.1136/sextrans-2018-053591 30126949 PMC6383506

[50] Kenneth M, Kevin M, Kenneth L, HIV infection and PrEP use are independently associated with increasing diagnoses of bacterial sexually transmitted infections (BSTI) in men accessing care at a Boston community health center (CHC): 2005-2015. http://www.natap.org/2016/IDSA/IDSA_25.htm. Accessed March 14, 2019.

[51] Kipyego J, Kaguirie E, Ballidawa JB, Prevalence and correlates of sexually transmitted infections (STIs) among HIV-1 sero-discordant couples in Western, Kenya. AIDS Res Hum Retroviruses. 2016;32(suppl 1):278.

[52] Knapper C, Birley H, Couzens Z, Parker I, Jones A Comorbidity, polypharmacy and renal impairment: the experience of managing a PrEP cohort in an integrated sexual health service setting. HIV Med. 2018;19(suppl 2):S47.

[53] Cotte L, Veyer D, Charreau I, Anal, oral and genital distribution of HPV in PrEP-users MSM: results at baseline of the ANRS IPERGAY HPV Sub-Study. Paper presented at: 22nd International AIDS Conference; July 23-27, 2018; Amsterdam, the Netherlands.

[54] Lal L, Audsley J, Murphy DA, ; VicPrEP Study Team Medication adherence, condom use and sexually transmitted infections in Australian preexposure prophylaxis users. AIDS. 2017;31(12):1709-1714. doi:10.1097/QAD.0000000000001519 28700394

[55] Lalley-Chareczko L, Clark D, Conyngham C, Delivery of TDF/FTC for pre-exposure prophylaxis to prevent HIV-1 acquisition in young adult men who have sex with men and transgender women of color using a urine adherence assay. J Acquir Immune Defic Syndr. 2018;79(2):173-178. doi:10.1097/QAI.0000000000001772 29905593

[56] Liu AY, Cohen SE, Vittinghoff E, Preexposure prophylaxis for HIV infection integrated with municipal- and community-based sexual health services. JAMA Intern Med. 2016;176(1):75-84. doi:10.1001/jamainternmed.2015.4683 26571482 PMC5042323

[57] La Fata L, Cotte L, Godinot M, High rate of asymptomatic bacterial sexually transmitted infections (STIs) in men who have sex with men on pre exposure prophylaxis (PrEP). Open Forum Infect Dis. 2017;4(suppl_1):S669. doi:10.1093/ofid/ofx163.1786

[58] Marcus JL, Glidden DV, Mayer KH, No evidence of sexual risk compensation in the iPrEx trial of daily oral HIV preexposure prophylaxis. PLoS One. 2013;8(12):e81997. doi:10.1371/journal.pone.0081997 24367497 PMC3867330

[59] Marcus JL, Glidden DV, McMahan V, Daily oral emtricitabine/tenofovir preexposure prophylaxis and herpes simplex virus type 2 among men who have sex with men. PLoS One. 2014;9(3):e91513. doi:10.1371/journal.pone.0091513 24637511 PMC3956614

[60] Marcus JL, Hurley LB, Hare CB, Preexposure prophylaxis for HIV prevention in a large integrated health care system: adherence, renal safety, and discontinuation. J Acquir Immune Defic Syndr. 2016;73(5):540-546. doi:10.1097/QAI.0000000000001129 27851714 PMC5424697

[61] Mayer KH, Maloney KM, Levine K, Sociodemographic and clinical factors associated with increasing bacterial sexually transmitted infection diagnoses in men who have sex with men accessing care at a Boston community health center (2005-2015). Open Forum Infect Dis. 2017;4(4):ofx214. doi:10.1093/ofid/ofx214 29181421 PMC5695616

[62] McCormack S, Dunn D Pragmatic open-label randomised trial of preexposure prophylaxis: the PROUD Study. Top Antivir Med. 2015;23(E-1):9-10.

[63] Molina JM, Charreau I, Chidiac C, ; ANRS IPERGAY Study Group Post-exposure prophylaxis with doxycycline to prevent sexually transmitted infections in men who have sex with men: an open-label randomised substudy of the ANRS IPERGAY trial. Lancet Infect Dis. 2018;18(3):308-317. doi:10.1016/S1473-3099(17)30725-9 29229440

[64] Molina JM, Charreau I, Spire B, ; ANRS IPERGAY Study Group Efficacy, safety, and effect on sexual behaviour of on-demand pre-exposure prophylaxis for HIV in men who have sex with men: an observational cohort study. Lancet HIV. 2017;4(9):e402-e410. doi:10.1016/S2352-3018(17)30089-9 28747274

[65] Nguyen VK, Greenwald ZR, Trottier H, Incidence of sexually transmitted infections before and after preexposure prophylaxis for HIV. AIDS. 2018;32(4):523-530. doi:10.1097/QAD.0000000000001718 29239887 PMC5865505

[66] Nguyen VK, Trottier H, Tossa HG, Increased rate of *C trachomatis* infection after being prescribed PrEP. J Int AIDS Soc. 2016;19(8)(suppl 7):27-28.

[67] Noret M, Balavoine S, Pintado C, Daily or on-demand oral tenofovir disoproxil fumarate/emtricitabine for HIV pre-exposure prophylaxis: experience from a hospital-based clinic in France. AIDS. 2018;32(15):2161-2169. doi:10.1097/QAD.0000000000001939 30212403

[68] Phanuphak N, Sungsing T, Jantarapakde J, Princess PrEP program: the first key population-led model to deliver pre-exposure prophylaxis to key populations by key populations in Thailand. Sex Health. 2018;15(6):542-555. doi:10.1071/SH18065 30249317

[69] Hechter R, Chen LH, Yu K Healthcare utilization and STI incidence in young men on pre-exposure prophylaxis (PrEP) compared to young men who are not on PrEP: the PrEPARE Study. Paper presented at: 22nd International AIDS Conference; July 23-27, 2018; Amsterdam, the Netherlands.

[70] Reyniers T, Nöstlinger C, Laga M, Choosing between daily and event-driven pre-exposure prophylaxis: results of a Belgian PrEP demonstration project. J Acquir Immune Defic Syndr. 2018;79(2):186-194. doi:10.1097/QAI.0000000000001791 29975211

[71] Solomon MM, Mayer KH, Glidden DV, ; iPrEx Study Team Syphilis predicts HIV incidence among men and transgender women who have sex with men in a preexposure prophylaxis trial. Clin Infect Dis. 2014;59(7):1020-1026. doi:10.1093/cid/ciu450 24928295 PMC4166980

[72] Tabidze I, Rusie L, Hendry C, Baker KK Primary and secondary syphilis and pre exposure prophylaxis (PrEP), Chicago, IL, 2014-2016. Sex Transm Dis. 2018;45(suppl 2):S74.

[73] Tiberio PJ, Williams K, Barakat LA, Edelman EJ, Virata M, Ogbuagu O Prepared: implementation of a pre-exposure prophylaxis (PrEP) program in a hospital-based HIV clinic. J Gen Intern Med. 2016;31(2)(suppl 1):S904-S905.

[74] Tiraboschi J, Brodnicki E, Brady M, Acute hepatitis C in the PROUD pilot study. HIV Med. 2014;15(suppl 3):S16.

[75] Traeger M, Asselin J, Price B, Changes, patterns and predictors of sexually transmitted infections in gay and bisexual men using PrEP: interim analysis from the PrEPX demonstration study. J Int AIDS Soc. 2018;21(suppl 6):80.

[76] Volk JE, Marcus JL, Phengrasamy T, No new HIV infections with increasing use of HIV preexposure prophylaxis in a clinical practice setting. Clin Infect Dis. 2015;61(10):1601-1603. doi:10.1093/cid/civ778 26334052 PMC4809999

[77] Zablotska I, Vaccher S, Gianacas C, STI rates among gay men taking daily antiretrovirals for pre-exposure prophylaxis of HIV: the NSW demonstration project prelude. Sex Transm Infect. 2015;91(suppl 2):A78. doi:10.1136/sextrans-2015-052270.209

[78] Cotte L, Cua E, Reynes J, ; Dat’AIDS Study Group Hepatitis C virus incidence in HIV-infected and in preexposure prophylaxis (PrEP)-using men having sex with men. Liver Int. 2018;38(10):1736-1740. doi:10.1111/liv.13922 29959866

[79] Hoornenborg E, Coyer L, Achterbergh R, High incidence of hepatitis C virus (re-)infections among PrEP users in the Netherlands: implications for prevention, monitoring and treatment. J Viral Hepat. 2018;25:192. doi:10.1111/jvh.03_12935

[80] Celum C, Delany-Moretlwe S, Hosek S, Risk behavior, perception, and reasons for PrEP among young African women in HPTN 082. Paper presented at: 2019 Conference on Retroviruses and Opportunistic Infections; March 4-7, 2019; Seattle, WA.

[81] Hoornenborg E, Achterbergh RC, van der Loeff MFS, ; Amsterdam PrEP Project Team in the HIV Transmission Elimination AMsterdam Initiative Men who have sex with men more often chose daily than event-driven use of pre-exposure prophylaxis: baseline analysis of a demonstration study in Amsterdam. J Int AIDS Soc. 2018;21(3):e25105. doi:10.1002/jia2.25105 29603900 PMC5878413

[82] Montaño MA, Dombrowski JC, Dasgupta S, Changes in sexual behavior and STI diagnoses among MSM initiating PrEP in a clinic setting. AIDS Behav. 2019;23(2):548-555. doi:10.1007/s10461-018-2252-9 30117076 PMC6368873

[83] Page K, Akolo O, Redd R, Baseline sexually transmitted infections (STI) and patient retention among patients enrolling in PrEP in the Baltimore City Health Department sexual health clinic. Sex Transm Dis. 2018;45(suppl 2):S64.

[84] Parsons J, Rendina HJ, Whitfield T, Grov C Changes in rectal STI incidence and behavioral HIV risk before, during, and after PrEP in a national sample of gay and bisexual men in the United States. J Int AIDS Soc. 2018;21(suppl 6):14.

[85] Antonucci S, Desai M, Dolling D, The UK PROUD PrEP Pilot Study: a baseline analysis. Paper presented at: 20th International AIDS Conference; July 20-25, 2014; Melbourne, Australia.

[86] Volk JE, Marcus JL, Phengrasamy T, Hare CB Incident hepatitis C virus infections among users of HIV preexposure prophylaxis in a clinical practice setting. Clin Infect Dis. 2015;60(11):1728-1729. doi:10.1093/cid/civ129 25694649 PMC4850931

[87] Jenness SM, Weiss KM, Goodreau SM, Incidence of gonorrhea and chlamydia following human immunodeficiency virus preexposure prophylaxis among men who have sex with men: a modeling study. Clin Infect Dis. 2017;65(5):712-718. doi:10.1093/cid/cix439 28505240 PMC5848234

[88] Chow EPF, Callander D, Fairley CK, ; ACCESS Collaboration Increased syphilis testing of men who have sex with men: greater detection of asymptomatic early syphilis and relative reduction in secondary syphilis. Clin Infect Dis. 2017;65(3):389-395. doi:10.1093/cid/cix326 28419198

[89] Unitaid Market and technology landscape: HIV rapid diagnostic tests for self-testing, 4th edition. http://www.unitaid.org/assets/HIVST-landscape-report.pdf. Published July 2018. Accessed April 2, 2019.

[90] Patton ME, Kidd S, Llata E, Extragenital gonorrhea and chlamydia testing and infection among men who have sex with men—STD Surveillance Network, United States, 2010-2012. Clin Infect Dis. 2014;58(11):1564-1570. doi:10.1093/cid/ciu184 24647015 PMC4666527

[91] Sultan B, White JA, Fish R, The “3 in 1” Study: pooling self-taken pharyngeal, urethral, and rectal samples into a single sample for analysis for detection of *Neisseria gonorrhoeae* and *Chlamydia trachomatis* in men who have sex with men. J Clin Microbiol. 2016;54(3):650-656. doi:10.1128/JCM.02460-15 26719439 PMC4767962

