# Supplementary material for: Global Epidemiologic Characteristics of Sexually Transmitted Infections Among Individuals Using Preexposure Prophylaxis for the Prevention of HIV Infection: A Systematic Review and Meta-analysis
Source: JAMA Netw Open. 2019 Dec 11;2(12):e1917134. doi: 10.1001/jamanetworkopen.2019.17134 (PMC6991203; doi:10.1001/jamanetworkopen.2019.17134)
Supplement: Supplement. — eAppendix 1. Search Methodology eAppendix 2. Data Variables Used in Data Extraction eAppendix 3. Forest Plots by Pathogen and Subgroups eFigure 1. Random Effects Meta-Analysis of Chlamydia Prevalence eTable 1. Meta-Regression Results for the Predictors of Chlamydia Prevalence and Sources of Between-Study Heterogeneity eFigure 2. Random Effects Meta-Analysis of Gonorrhea Prevalence eTable 2. Meta-Regression Results for the Predictors of Gonorrhea Prevalence and Sources of Between-Study Heterogeneity eFigure 3. Random Effects Meta-Analysis of Early Syphilis Prevalence eTable 3. Meta-Regression Results for the Predictors of Early Syphilis Prevalence and Sources of Between-Study Heterogeneity eFigure 4. Random Effects Meta-Analysis of Any Chlamydia, Gonorrhea, or Early Syphilis Prevalence eTable 4. Meta-Regression Results for the Predictors of Any Chlamydia, Gonorrhea, or Early Syphilis Prevalence and Sources of Between-Study Heterogeneity eFigure 5. Random Effects Meta-Analysis of Hepatitis B Prevalence eFigure 6. Random Effects Meta-Analysis of Hepatitis C Prevalence eFigure 7. Random Effects Meta-Analysis of Chlamydia Incidence eTable 5. Meta-Regression Results for the Predictors of Chlamydia Incidence and Sources of Between-Study Heterogeneity eFigure 8. Random Effects Meta-Analysis of Gonorrhea Incidence eTable 6. Meta-Regression Results for the Predictors of Gonorrhea Incidence and Sources of Between-Study Heterogeneity eFigure 9. Random Effects Meta-Analysis of Early Syphilis Incidence eTable 7. Meta-Regression Results for the Predictors of Early Syphilis Incidence and Sources of Between-Study Heterogeneity eFigure 10. Random Effects Meta-Analysis of Any Chlamydia, Gonorrhea or Early Syphilis Incidence eFigure 11. Random Effects Meta-Analysis of Hepatitis C Incidence [file jamanetwopen-2-e1917134-s001.pdf]

Supplementary Online Content

Ong JJ, Baggaley RC, Wi TE, et al. Global epidemiologic characteristics of sexually transmitted infections among individuals using preexposure prophylaxis for the prevention of HIV infection: a systematic review and meta-analysis. *JAMA Netw Open.* 2019;2(12):e1917134. doi:10.1001/jamanetworkopen.2019.17134

eAppendix 1. Search Methodology
eAppendix 2. Data Variables Used in Data Extraction
eAppendix 3. Forest Plots by Pathogen and Subgroups
eFigure 1. Random Effects Meta-Analysis of Chlamydia Prevalence
eTable 1. Meta-Regression Results for the Predictors of Chlamydia Prevalence and Sources of Between-Study Heterogeneity
eFigure 2. Random Effects Meta-Analysis of Gonorrhea Prevalence
eTable 2. Meta-Regression Results for the Predictors of Gonorrhea Prevalence and Sources of Between-Study Heterogeneity
eFigure 3. Random Effects Meta-Analysis of Early Syphilis Prevalence
eTable 3. Meta-Regression Results for the Predictors of Early Syphilis Prevalence and Sources of Between-Study Heterogeneity
eFigure 4. Random Effects Meta-Analysis of Any Chlamydia, Gonorrhea, or Early Syphilis Prevalence
eTable 4. Meta-Regression Results for the Predictors of Any Chlamydia, Gonorrhea, or Early Syphilis Prevalence and Sources of Between-Study Heterogeneity
eFigure 5. Random Effects Meta-Analysis of Hepatitis B Prevalence
eFigure 6. Random Effects Meta-Analysis of Hepatitis C Prevalence
eFigure 7. Random Effects Meta-Analysis of Chlamydia Incidence
eTable 5. Meta-Regression Results for the Predictors of Chlamydia Incidence and Sources of Between-Study Heterogeneity
eFigure 8. Random Effects Meta-Analysis of Gonorrhea Incidence
eTable 6. Meta-Regression Results for the Predictors of Gonorrhea Incidence and Sources of Between-Study Heterogeneity
eFigure 9. Random Effects Meta-Analysis of Early Syphilis Incidence
eTable 7. Meta-Regression Results for the Predictors of Early Syphilis Incidence and Sources of Between-Study Heterogeneity
eFigure 10. Random Effects Meta-Analysis of Any Chlamydia, Gonorrhea or Early Syphilis Incidence
eFigure 11. Random Effects Meta-Analysis of Hepatitis C Incidence

This supplementary material has been provided by the authors to give readers additional information about their work.

© 2019 Ong JJ et al. *JAMA Network Open.*

eAppendix 1. Search Methodology

1 Search methodology

Nine databases were searched on 20 November 2018. The search strategies looked for information on sexually transmitted infections and pre-exposure prophylaxis, no limits were added. The search strategy was compiled in the OvidSP Medline database by Jane Falconer, using the list of sexually transmitted infections listed by the Centers for Disease Control and Prevention, and using terms used by the Cochrane STIs group.[1] The search strategy was refined with the project team until the results retrieved reflected the scope of the project. The final Medline search was amended to run across the other databases.

The databases searched were:

1. OvidSP Medline and In-Process & Other Non-Indexed Citations and Daily, 1946 to November 19, 2018.
2. OvidSP Embase, 1974 to 2018 November 19
3. OvidSP Global Health, 1910 to 2018 Week 45
4. OvidSP EconLit, 1886 to November 15, 2018
5. EBSCO CINAHL Plus, complete database
6. EBSCO Africa-Wide Information, complete database
7. Web of Science Core Collection, which included:
    a. Science Citation Index Expanded, 1970 – 19 November 2018
    b. Social Sciences Citation Index, 1970– 19 November 2018
    c. Arts & Humanities Citation Index, 1975– 19 November 2018
    d. Conference Proceedings Citation Index- Science, 1990– 19 November 2018
    e. Conference Proceedings Citation Index- Social Science & Humanities, 1990– 19 November 2018
    f. Emerging Sources Citation Index, 2015– 19 November 2018
8. VHL LILACS, complete database
9. OvidSP Northern Light Life Sciences Conference Abstracts, 2010 – 2018 Week 45

Complete search strategies are provided below.

2 Search results

Search results were uploaded to EndNote X9 and deduplicated. using a technique developed at the University of Leeds.[2] Number of results pre- and post- deduplication are listed in the table below.

Database name	EndNote import order	Number of references before deduplication	Number of references after deduplication

[1] List of CDC STIs at https://www.cdc.gov/std/general/default.htm. Initial list of STI terms derived from Obiero J, Mwethera PG, Wiysonge CS. Topical microbicides for prevention of sexually transmitted infections. Cochrane Database of Systematic Reviews 2012, Issue 6. Art. No.: CD007961. https://doi.org/10.1002/14651858.CD007961.pub2.

[2] Deduplication method is published at http://medhealth.leeds.ac.uk/download/2518/auhe_duplicate_checking_guide.

© 2019 Ong JJ et al. *JAMA Network Open.*

Medline	1	368	366
Embase	2	1248	870
Medline in process[3]	3	131	52
Global Health	4	526	284
EconLit	5	0	0
CINAHL Plus	6	197	30
Africa-Wide	7	58	3
Web of Science	8	677	235
LILACS	9	1	1
Northern Light	10	119	67
**Total**		3325	1908

3 Search strategies

3.1 OvidSP Medline

Database name	Medline
Database platform	OvidSP
Dates of database coverage	1946 to November Week 2 2018
Date searched	20/11/2018
Searched by	JF
Number of hits	368

1 Sexually Transmitted Diseases/ (23298)
2 sexually transmitted disease*.ti,ab. (14601)
3 sexually transmissible disease*.ti,ab. (222)
4 sexually transmitted infection*.ti,ab. (9921)
5 sexually transmissible infection*.ti,ab. (414)
6 sexually transmitted infectious disease*.ti,ab. (23)
7 sexually transmissible infectious disease*.ti,ab. (0)
8 sexually transmitted disorder*.ti,ab. (13)
9 sexually transmissible disorder*.ti,ab. (10)
10 STI.ti,ab. (6871)
11 STD.ti,ab. (8633)
12 genital ulcer*.ti,ab. (2136)

[3] Medline results are split into Medline and Medline In-Process & Other Non-Indexed Citations and Daily updates to allow automatic deduplication to retain potential duplicates from Embase.

© 2019 Ong JJ et al. *JAMA Network Open.*

13 genital infection*.ti,ab. (2474)
14 genital disorder*.ti,ab. (88)
15 venereal disease*.ti,ab. (3734)
16 venereal infection*.ti,ab. (139)
17 venereal disorder*.ti,ab. (1)
18 Sexually Transmitted Diseases, Bacterial/ (985)
19 vaginosis, bacterial/ (2812)
20 exp gardnerella/ (1114)
21 mobiluncus/ (62)
22 bacterial vaginosis.ti,ab. (3160)
23 gardnerella vaginalis.ti,ab. (1128)
24 mobiluncus.ti,ab. (224)
25 bacterial vaginitis.ti,ab. (58)
26 Chancroid/ (852)
27 Haemophilus ducreyi/ (646)
28 chancroid?.ti,ab. (815)
29 haemophilus ducreyi.ti,ab. (719)
30 hemophilus ducreyi.ti,ab. (37)
31 Chlamydia Infections/ (14801)
32 Lymphogranuloma Venereum/ (1542)
33 Chlamydia/ (2800)
34 chlamydia trachomatis/ (11454)
35 chlamydia.ti,ab. (22000)
36 lymphogranuloma venereum.ti,ab. (970)
37 LGV.ti,ab. (486)
38 lymphogranuloma inguinale.ti,ab. (53)
39 Gonorrhea/ (13295)
40 Neisseria gonorrhoeae/ (9317)
41 gonorrhea.ti,ab. (5992)
42 neisseria gonorrhoeae.ti,ab. (8569)
43 gonococcal urethritis.ti,ab. (1160)
44 gonococci.ti,ab. (1907)
45 Granuloma Inguinale/ (585)
46 Calymmatobacterium/ (71)
47 granuloma inguinale.ti,ab. (317)
48 Calymmatobacterium granulomatis.ti,ab. (33)
49 granuloma venereum.ti,ab. (48)
50 donovanosis.ti,ab. (203)
51 Mycoplasma Infections/ (7921)
52 Mycoplasma genitalium/ (595)
53 mycoplasma genitalium.ti,ab. (1005)
54 exp Syphilis/ (26708)
55 Treponema pallidum/ (3787)
56 syphilis.ti,ab. (20533)

© 2019 Ong JJ et al. *JAMA Network Open.*

57 chancre.ti,ab. (384)
58 treponema pallidum.ti,ab. (3561)
59 condylomata lata.ti,ab. (38)
60 Sexually Transmitted Diseases, Viral/ (1413)
61 Hepatitis, Viral, Human/ (11041)
62 Hepatitis Viruses/ (1507)
63 exp Hepadnaviridae Infections/ (54902)
64 Hepatitis B virus/ (24874)
65 Hepadnaviridae/ (209)
66 hepatitis-b.ti,ab. (65510)
67 hep-b.ti,ab. (91)
68 (hepatitis adj5 b).ti,ab. (69910)
69 hbv.ti,ab. (34075)
70 exp Hepatitis D/ (2239)
71 Hepatitis Delta Virus/ (1960)
72 hepatitis-d.ti,ab. (897)
73 (hepatitis adj5 delta).ti,ab. (2207)
74 hep-d.ti,ab. (5)
75 (hepatitis adj5 d).ti,ab. (1825)
76 (hep adj5 d).ti,ab. (158)
77 hdv.ti,ab. (2011)
78 Herpes Simplex/ (13838)
79 Herpes genitalis/ (4523)
80 simplexvirus/ (17028)
81 "herpesvirus 1, human"/ (9837)
82 "herpesvirus 2, human"/ (4095)
83 herpes genitalis.ti,ab. (310)
84 genital herpes.ti,ab. (2770)
85 herpes virus.ti,ab. (4737)
86 herpes simplex.ti,ab. (36737)
87 herpesvirus.ti,ab. (19753)
88 simplexvirus.ti,ab. (27)
89 "HSV-1".ti,ab. (10914)
90 "HHV-1".ti,ab. (64)
91 "HSV-2".ti,ab. (5476)
92 "HHV-2".ti,ab. (28)
93 papillomavirus infections/ (22575)
94 condylomata acuminata/ (5059)
95 "Human papillomavirus 6"/ (396)
96 "Human papillomavirus 18"/ (1653)
97 "Human papillomavirus 11"/ (353)
98 "Human papillomavirus 16"/ (4149)
99 human papillomavirus.ti,ab. (27122)
100 genital wart*.ti,ab. (2040)

© 2019 Ong JJ et al. *JAMA Network Open.*

101 anogenital wart*.ti,ab. (523)
102 anorectal wart*.ti,ab. (4)
103 penile wart*.ti,ab. (54)
104 condylomata acuminata.ti,ab. (1021)
105 condyloma.ti,ab. (2046)
106 cervical cancer.ti,ab. (34842)
107 HPV.ti,ab. (32230)
108 candidiasis/ (20581)
109 candidiasis, vulvovaginal/ (3359)
110 candida albicans/ (23171)
111 candidiasis.ti,ab. (12809)
112 candida.ti,ab. (50266)
113 candidal vaginitis.ti,ab. (103)
114 candidosis.ti,ab. (1680)
115 vulvovaginitis.ti,ab. (953)
116 vulvitis.ti,ab. (304)
117 vulvovaginal candidiasis.ti,ab. (865)
118 vulvodynia.ti,ab. (511)
119 balanitis.ti,ab. (629)
120 monilia albicans.ti,ab. (40)
121 monilial infection.ti,ab. (24)
122 exp Trichomonas Infections/ (5966)
123 Trichomonas vaginalis/ (3416)
124 trichomoniasis.ti,ab. (2717)
125 trichomoniases.ti,ab. (2)
126 trichomonas vaginalis.ti,ab. (4131)
127 trichomonas vaginitides.ti,ab. (0)
128 trichomonas vaginitis.ti,ab. (241)
129 trichomonas vaginali.ti,ab. (1)
130 or/1-129 (414688)
131 Pre-Exposure Prophylaxis/ (1184)
132 pre-exposure prophylaxis.ti,ab. (1171)
133 preexposure prophylaxis.ti,ab. (507)
134 prep.ti,ab. (2834)
135 or/131-134 (3869)
136 130 and 135 (368)

3.2 OvidSP Embase

Database name	Embase
Database platform	OvidSP
Dates of database coverage	1974 to 2018 November 19
Date searched	20/11/2018

© 2019 Ong JJ et al. *JAMA Network Open.*

Searched by	JF
Number of hits	1248

1 sexually transmitted disease/ (41708)
2 sexually transmitted disease*.ti,ab. (17402)
3 sexually transmissible disease*.ti,ab. (245)
4 sexually transmitted infection*.ti,ab. (15003)
5 sexually transmitted infectious disease*.ti,ab. (30)
6 sexually transmissible infectious disease*.ti,ab. (1)
7 sexually transmitted disorder*.ti,ab. (17)
8 sexually transmissible disorder*.ti,ab. (3)
9 STI.ti,ab. (12283)
10 STD.ti,ab. (13534)
11 genital ulcer*.ti,ab. (3230)
12 genital infection*.ti,ab. (3378)
13 genital disorder*.ti,ab. (113)
14 venereal disease*.ti,ab. (2680)
15 venereal infection*.ti,ab. (130)
16 venereal disorder*.ti,ab. (7)
17 exp vaginitis/ (13939)
18 gardnerella vaginalis/ (2173)
19 mobiluncus/ (301)
20 bacterial vaginosis.ti,ab. (4599)
21 gardnerella vaginalis.ti,ab. (1549)
22 mobiluncus.ti,ab. (309)
23 bacterial vaginitis.ti,ab. (92)
24 ulcus molle/ (1210)
25 haemophilus ducreyi/ (1040)
26 chancroid?.ti,ab. (847)
27 haemophilus ducreyi.ti,ab. (797)
28 hemophilus ducreyi.ti,ab. (20)
29 chlamydia/ (6730)
30 chlamydia trachomatis/ (17758)
31 chlamydiasis/ (15213)
32 lymphogranuloma venereum/ (1354)
33 chlamydia.ti,ab. (29208)
34 lymphogranuloma venereum.ti,ab. (980)
35 LGV.ti,ab. (752)
36 lymphogranuloma inguinale.ti,ab. (13)
37 gonorrhea/ (15390)
38 Neisseria gonorrhoeae/ (14434)
39 gonorrhea.ti,ab. (6408)
40 neisseria gonorrhoeae.ti,ab. (10140)
41 gonococcal urethritis.ti,ab. (1301)

© 2019 Ong JJ et al. *JAMA Network Open.*

42 gonococci.ti,ab. (1946)
43 granuloma inguinale/ (561)
44 klebsiella/ (14700)
45 klebsiella granulomatis/ (24)
46 granuloma inguinale.ti,ab. (200)
47 Calymmatobacterium granulomatis.ti,ab. (35)
48 granuloma venereum.ti,ab. (19)
49 donovanosis.ti,ab. (248)
50 mycoplasma genitalium/ (1725)
51 mycoplasmosis/ (1437)
52 mycoplasma genitalium.ti,ab. (1456)
53 exp syphilis/ (22619)
54 Treponema pallidum/ (5429)
55 condyloma latum/ (104)
56 syphilis.ti,ab. (20948)
57 chancre.ti,ab. (388)
58 treponema pallidum.ti,ab. (4043)
59 condylomata lata.ti,ab. (55)
60 virus hepatitis/ (19656)
61 hepadnavirus infection/ (85)
62 exp hepatitis b/ (92711)
63 exp Hepatitis B virus/ (50299)
64 hepadnaviridae/ (170)
65 hepatitis-b.ti,ab. (101147)
66 hep-b.ti,ab. (335)
67 (hepatitis adj5 b).ti,ab. (107479)
68 hbv.ti,ab. (61238)
69 delta agent hepatitis/ (2835)
70 Hepatitis delta virus/ (3446)
71 hepatitis-d.ti,ab. (1438)
72 (hepatitis adj5 delta).ti,ab. (2891)
73 hep-d.ti,ab. (5)
74 (hepatitis adj5 d).ti,ab. (2912)
75 (hep adj5 d).ti,ab. (173)
76 hdv.ti,ab. (3295)
77 herpes simplex/ (17575)
78 genital herpes/ (5706)
79 simplexvirus/ (628)
80 exp Herpes simplex virus/ (31602)
81 herpes genitalis.ti,ab. (414)
82 genital herpes.ti,ab. (3569)
83 herpes virus.ti,ab. (6922)
84 herpes simplex.ti,ab. (42909)
85 herpesvirus.ti,ab. (22067)

© 2019 Ong JJ et al. *JAMA Network Open.*

86 simplexvirus.ti,ab. (37)
87 "HSV-1".ti,ab. (13274)
88 "HHV-1".ti,ab. (94)
89 "HSV-2".ti,ab. (6792)
90 "HHV-2".ti,ab. (44)
91 papillomavirus infection/ (11864)
92 exp condyloma acuminatum/ (7832)
93 Wart virus/ (33380)
94 human papillomavirus type 6/ (2179)
95 Human papillomavirus type 18/ (5853)
96 human papillomavirus type 11/ (1878)
97 human papillomavirus type 16/ (11308)
98 human papillomavirus.ti,ab. (35689)
99 genital wart*.ti,ab. (3062)
100 anogenital wart*.ti,ab. (745)
101 anorectal wart*.ti,ab. (7)
102 penile wart*.ti,ab. (67)
103 condylomata acuminata.ti,ab. (1209)
104 condyloma.ti,ab. (2799)
105 cervical cancer.ti,ab. (53862)
106 HPV.ti,ab. (49090)
107 candidiasis/ (25077)
108 vagina candidiasis/ (4538)
109 Candida albicans/ (50400)
110 candidiasis.ti,ab. (17817)
111 candida.ti,ab. (69884)
112 candidal vaginitis.ti,ab. (119)
113 candidosis.ti,ab. (2142)
114 vulvovaginitis.ti,ab. (1259)
115 vulvitis.ti,ab. (353)
116 vulvovaginal candidiasis.ti,ab. (1409)
117 vulvodynia.ti,ab. (1001)
118 balanitis.ti,ab. (940)
119 monilia albicans.ti,ab. (11)
120 monilial infection.ti,ab. (12)
121 exp trichomoniasis/ (4501)
122 Trichomonas vaginalis/ (5983)
123 trichomoniasis.ti,ab. (2720)
124 trichomoniases.ti,ab. (3)
125 trichomonas vaginalis.ti,ab. (4661)
126 trichomonas vaginitides.ti,ab. (0)
127 trichomonas vaginitis.ti,ab. (129)
128 trichomonas vaginali.ti,ab. (0)
129 or/1-128 (593600)

© 2019 Ong JJ et al. *JAMA Network Open.*

130 pre-exposure prophylaxis/ (2315)
131 pre-exposure prophylaxis.ti,ab. (2297)
132 preexposure prophylaxis.ti,ab. (728)
133 prep.ti,ab. (7044)
134 or/130-133 (8569)
135 129 and 134 (1248)

3.3 OvidSP Medline In-Process & Other Non-Indexed Citations and Daily updates

Database name	Ovid MEDLINE(R) and In-Process & Other Non-Indexed Citations and Daily
Database platform	OvidSP
Dates of database coverage	1946 to November 19, 2018
Date searched	20/11/2018
Searched by	JF
Number of hits	131

1 Sexually Transmitted Diseases/ (23309)
2 sexually transmitted disease*.ti,ab. (15335)
3 sexually transmissible disease*.ti,ab. (228)
4 sexually transmitted infection*.ti,ab. (11308)
5 sexually transmissible infection*.ti,ab. (520)
6 sexually transmitted infectious disease*.ti,ab. (26)
7 sexually transmissible infectious disease*.ti,ab. (0)
8 sexually transmitted disorder*.ti,ab. (13)
9 sexually transmissible disorder*.ti,ab. (10)
10 STI.ti,ab. (7798)
11 STD.ti,ab. (9262)
12 genital ulcer*.ti,ab. (2362)
13 genital infection*.ti,ab. (2620)
14 genital disorder*.ti,ab. (100)
15 venereal disease*.ti,ab. (4324)
16 venereal infection*.ti,ab. (149)
17 venereal disorder*.ti,ab. (2)
18 Sexually Transmitted Diseases, Bacterial/ (985)
19 vaginosis, bacterial/ (2813)
20 exp gardnerella/ (1115)
21 mobiluncus/ (62)
22 bacterial vaginosis.ti,ab. (3487)
23 gardnerella vaginalis.ti,ab. (1213)
24 mobiluncus.ti,ab. (237)
25 bacterial vaginitis.ti,ab. (61)
26 Chancroid/ (852)

© 2019 Ong JJ et al. *JAMA Network Open.*

27 Haemophilus ducreyi/ (646)
28 chancroid?.ti,ab. (868)
29 haemophilus ducreyi.ti,ab. (743)
30 hemophilus ducreyi.ti,ab. (37)
31 Chlamydia Infections/ (14809)
32 Lymphogranuloma Venereum/ (1543)
33 Chlamydia/ (2803)
34 chlamydia trachomatis/ (11463)
35 chlamydia.ti,ab. (23252)
36 lymphogranuloma venereum.ti,ab. (1054)
37 LGV.ti,ab. (537)
38 lymphogranuloma inguinale.ti,ab. (81)
39 Gonorrhea/ (13303)
40 Neisseria gonorrhoeae/ (9325)
41 gonorrhea.ti,ab. (6399)
42 neisseria gonorrhoeae.ti,ab. (9017)
43 gonococcal urethritis.ti,ab. (1229)
44 gonococci.ti,ab. (1958)
45 Granuloma Inguinale/ (585)
46 Calymmatobacterium/ (71)
47 granuloma inguinale.ti,ab. (354)
48 Calymmatobacterium granulomatis.ti,ab. (37)
49 granuloma venereum.ti,ab. (66)
50 donovanosis.ti,ab. (228)
51 Mycoplasma Infections/ (7924)
52 Mycoplasma genitalium/ (596)
53 mycoplasma genitalium.ti,ab. (1125)
54 exp Syphilis/ (26711)
55 Treponema pallidum/ (3789)
56 syphilis.ti,ab. (23281)
57 chancre.ti,ab. (446)
58 treponema pallidum.ti,ab. (3895)
59 condylomata lata.ti,ab. (46)
60 Sexually Transmitted Diseases, Viral/ (1413)
61 Hepatitis, Viral, Human/ (11045)
62 Hepatitis Viruses/ (1507)
63 exp Hepadnaviridae Infections/ (54963)
64 Hepatitis B virus/ (24909)
65 Hepadnaviridae/ (209)
66 hepatitis-b.ti,ab. (71485)
67 hep-b.ti,ab. (103)
68 (hepatitis adj5 b).ti,ab. (76057)
69 hbv.ti,ab. (37819)
70 exp Hepatitis D/ (2241)

© 2019 Ong JJ et al. *JAMA Network Open.*

71 Hepatitis Delta Virus/ (1962)
72 hepatitis-d.ti,ab. (997)
73 (hepatitis adj5 delta).ti,ab. (2316)
74 hep-d.ti,ab. (6)
75 (hepatitis adj5 d).ti,ab. (2000)
76 (hep adj5 d).ti,ab. (164)
77 hdv.ti,ab. (2174)
78 Herpes Simplex/ (13850)
79 Herpes genitalis/ (4523)
80 simplexvirus/ (17034)
81 "herpesvirus 1, human"/ (9841)
82 "herpesvirus 2, human"/ (4096)
83 herpes genitalis.ti,ab. (330)
84 genital herpes.ti,ab. (2932)
85 herpes virus.ti,ab. (5173)
86 herpes simplex.ti,ab. (38343)
87 herpesvirus.ti,ab. (20826)
88 simplexvirus.ti,ab. (29)
89 "HSV-1".ti,ab. (11379)
90 "HHV-1".ti,ab. (69)
91 "HSV-2".ti,ab. (5764)
92 "HHV-2".ti,ab. (30)
93 papillomavirus infections/ (22600)
94 condylomata acuminata/ (5060)
95 "Human papillomavirus 6"/ (396)
96 "Human papillomavirus 18"/ (1654)
97 "Human papillomavirus 11"/ (353)
98 "Human papillomavirus 16"/ (4152)
99 human papillomavirus.ti,ab. (29925)
100 genital wart*.ti,ab. (2248)
101 anogenital wart*.ti,ab. (571)
102 anorectal wart*.ti,ab. (4)
103 penile wart*.ti,ab. (59)
104 condylomata acuminata.ti,ab. (1059)
105 condyloma.ti,ab. (2201)
106 cervical cancer.ti,ab. (39178)
107 HPV.ti,ab. (35834)
108 candidiasis/ (20595)
109 candidiasis, vulvovaginal/ (3360)
110 candida albicans/ (23199)
111 candidiasis.ti,ab. (14040)
112 candida.ti,ab. (54895)
113 candidal vaginitis.ti,ab. (107)
114 candidosis.ti,ab. (1732)

© 2019 Ong JJ et al. *JAMA Network Open.*

115 vulvovaginitis.ti,ab. (1015)
116 vulvitis.ti,ab. (330)
117 vulvovaginal candidiasis.ti,ab. (1012)
118 vulvodynia.ti,ab. (575)
119 balanitis.ti,ab. (701)
120 monilia albicans.ti,ab. (41)
121 monilial infection.ti,ab. (24)
122 exp Trichomonas Infections/ (5967)
123 Trichomonas vaginalis/ (3419)
124 trichomoniasis.ti,ab. (2904)
125 trichomoniases.ti,ab. (2)
126 trichomonas vaginalis.ti,ab. (4377)
127 trichomonas vaginitides.ti,ab. (0)
128 trichomonas vaginitis.ti,ab. (251)
129 trichomonas vaginali.ti,ab. (2)
130 or/1-129 (444552)
131 Pre-Exposure Prophylaxis/ (1187)
132 pre-exposure prophylaxis.ti,ab. (1597)
133 preexposure prophylaxis.ti,ab. (642)
134 prep.ti,ab. (3495)
135 or/131-134 (4721)
136 130 and 134 (499)
137 limit 135 to ("in data review" or in process or publisher or "pubmed not medline") (131)

3.4 OvidSP Global Health

Database name	Global Health
Database platform	OvidSP
Dates of database coverage	1910 to 2018 Week 45
Date searched	20/11/2018
Searched by	JF
Number of hits	526

1 sexually transmitted diseases/ (31490)
2 sexually transmitted disease*.ti,ab. (7720)
3 sexually transmissible disease*.ti,ab. (71)
4 sexually transmitted infection*.ti,ab. (8017)
5 sexually transmissible infection*.ti,ab. (436)
6 sexually transmitted infectious disease*.ti,ab. (13)
7 sexually transmissible infectious disease*.ti,ab. (0)
8 sexually transmitted disorder*.ti,ab. (3)
9 sexually transmissible disorder*.ti,ab. (0)

© 2019 Ong JJ et al. *JAMA Network Open.*

10 STI.ti,ab. (4704)
11 STD.ti,ab. (4482)
12 genital ulcer*.ti,ab. (946)
13 genital infection*.ti,ab. (1029)
14 genital disorder*.ti,ab. (26)
15 venereal disease*.ti,ab. (3091)
16 venereal infection*.ti,ab. (150)
17 venereal disorder*.ti,ab. (5)
18 bacterial vaginitis/ (1579)
19 gardnerella vaginalis/ (572)
20 mobiluncus/ (89)
21 bacterial vaginosis.ti,ab. (2004)
22 gardnerella vaginalis.ti,ab. (603)
23 mobiluncus.ti,ab. (94)
24 bacterial vaginitis.ti,ab. (73)
25 chancroid/ (639)
26 haemophilus ducreyi/ (448)
27 chancroid?.ti,ab. (682)
28 haemophilus ducreyi.ti,ab. (326)
29 chlamydia/ (11371)
30 chlamydia trachomatis/ (8775)
31 chlamydia.ti,ab. (10501)
32 lymphogranuloma venereum.ti,ab. (813)
33 LGV.ti,ab. (280)
34 lymphogranuloma inguinale.ti,ab. (375)
35 gonorrhoea/ (7695)
36 neisseria gonorrhoeae/ (9190)
37 gonorrhea.ti,ab. (2088)
38 neisseria gonorrhoeae.ti,ab. (4306)
39 gonococcal urethritis.ti,ab. (939)
40 gonococci.ti,ab. (1640)
41 granuloma inguinale/ (550)
42 klebsiella granulomatis/ (78)
43 granuloma inguinale.ti,ab. (426)
44 Calymmatobacterium granulomatis.ti,ab. (27)
45 granuloma venereum.ti,ab. (180)
46 donovanosis.ti,ab. (117)
47 mycoplasma genitalium/ (657)
48 mycoplasma genitalium.ti,ab. (621)
49 exp syphilis/ (16126)
50 treponema pallidum/ (16075)
51 syphilis.ti,ab. (16173)
52 chancre.ti,ab. (517)
53 treponema pallidum.ti,ab. (2475)

© 2019 Ong JJ et al. *JAMA Network Open.*

54 condylomata lata.ti,ab. (14)
55 hepatitis b/ (30605)
56 hepatitis b virus/ (30605)
57 hepadnaviridae/ (30660)
58 hepatitis-b.ti,ab. (33116)
59 hep-b.ti,ab. (46)
60 (hepatitis adj5 b).ti,ab. (34525)
61 hbv.ti,ab. (19089)
62 hepatitis d/ (743)
63 hepatitis delta virus/ (927)
64 hepatitis-d.ti,ab. (543)
65 (hepatitis adj5 delta).ti,ab. (674)
66 hep-d.ti,ab. (0)
67 (hepatitis adj5 d).ti,ab. (1075)
68 (hep adj5 d).ti,ab. (13)
69 hdv.ti,ab. (748)
70 herpes simplex/ (5892)
71 herpes simplex genitalis/ (1012)
72 simplexvirus/ (8660)
73 human herpesviruses/ (4325)
74 human herpesvirus 1/ (2664)
75 human herpesvirus 2/ (2709)
76 herpes genitalis.ti,ab. (146)
77 herpes virus.ti,ab. (1733)
78 herpes simplex.ti,ab. (9780)
79 herpesvirus.ti,ab. (4773)
80 simplexvirus.ti,ab. (10)
81 "HSV-1".ti,ab. (2390)
82 "HHV-1".ti,ab. (40)
83 "HSV-2".ti,ab. (2379)
84 "HHV-2".ti,ab. (16)
85 genital warts/ (1115)
86 condyloma acuminatum/ (690)
87 human papillomavirus 6/ (795)
88 human papillomavirus 18/ (2121)
89 human papillomavirus 11/ (0)
90 human papillomavirus 16/ (3778)
91 human papillomavirus.ti,ab. (12661)
92 genital wart*.ti,ab. (1150)
93 anogenital wart*.ti,ab. (302)
94 anorectal wart*.ti,ab. (2)
95 penile wart*.ti,ab. (19)
96 condylomata acuminata.ti,ab. (192)
97 condyloma.ti,ab. (679)

© 2019 Ong JJ et al. *JAMA Network Open.*

98 cervical cancer.ti,ab. (10672)
99 HPV.ti,ab. (14601)
100 candida/ (57192)
101 candidosis/ (29665)
102 candida albicans/ (39429)
103 candidiasis.ti,ab. (8439)
104 candida.ti,ab. (53347)
105 candidal vaginitis.ti,ab. (114)
106 candidosis.ti,ab. (5771)
107 vulvovaginitis.ti,ab. (495)
108 vulvitis.ti,ab. (75)
109 vulvovaginal candidiasis.ti,ab. (808)
110 vulvodynia.ti,ab. (34)
111 balanitis.ti,ab. (212)
112 monilia albicans.ti,ab. (64)
113 monilial infection.ti,ab. (33)
114 trichomoniasis/ (3597)
115 trichomonas vaginalis/ (4749)
116 trichomoniasis.ti,ab. (2161)
117 trichomoniases.ti,ab. (2)
118 trichomonas vaginalis.ti,ab. (4433)
119 trichomonas vaginitides.ti,ab. (0)
120 trichomonas vaginitis.ti,ab. (119)
121 trichomonas vaginali.ti,ab. (0)
122 or/1-121 (195226)
123 pre-exposure prophylaxis.ti,ab. (901)
124 preexposure prophylaxis.ti,ab. (395)
125 prep.ti,ab. (1142)
126 or/123-125 (1610)
127 122 and 125 (526)

3.5 OvidSP EconLit

Database name	EconLit
Database platform	OvidSP
Dates of database coverage	1886 to November 15, 2018
Date searched	20/11/2018
Searched by	JF
Number of hits	0

1 sexually transmitted disease*.ti,ab. (75)
2 sexually transmissible disease*.ti,ab. (1)
3 sexually transmitted infection*.ti,ab. (80)

© 2019 Ong JJ et al. *JAMA Network Open.*

4 sexually transmissible infection*.ti,ab. (0)
5 sexually transmitted infectious disease*.ti,ab. (0)
6 sexually transmissible infectious disease*.ti,ab. (0)
7 sexually transmitted disorder*.ti,ab. (0)
8 sexually transmissible disorder*.ti,ab. (0)
9 STI.ti,ab. (112)
10 STD.ti,ab. (53)
11 genital ulcer*.ti,ab. (0)
12 genital infection*.ti,ab. (2)
13 genital disorder*.ti,ab. (0)
14 venereal disease*.ti,ab. (7)
15 venereal infection*.ti,ab. (0)
16 venereal disorder*.ti,ab. (0)
17 bacterial vaginosis.ti,ab. (0)
18 gardnerella vaginalis.ti,ab. (0)
19 mobiluncus.ti,ab. (0)
20 bacterial vaginitis.ti,ab. (0)
21 chancroid?.ti,ab. (0)
22 haemophilus ducreyi.ti,ab. (0)
23 chlamydia.ti,ab. (19)
24 lymphogranuloma venereum.ti,ab. (0)
25 LGV.ti,ab. (6)
26 lymphogranuloma inguinale.ti,ab. (0)
27 gonorrhea.ti,ab. (27)
28 neisseria gonorrhoeae.ti,ab. (0)
29 gonococcal urethritis.ti,ab. (0)
30 gonococci.ti,ab. (0)
31 granuloma inguinale.ti,ab. (0)
32 Calymmatobacterium granulomatis.ti,ab. (0)
33 granuloma venereum.ti,ab. (0)
34 donovanosis.ti,ab. (0)
35 mycoplasma genitalium.ti,ab. (0)
36 syphilis.ti,ab. (31)
37 chancre.ti,ab. (0)
38 treponema pallidum.ti,ab. (0)
39 condylomata lata.ti,ab. (0)
40 hepatitis-b.ti,ab. (34)
41 hep-b.ti,ab. (0)
42 (hepatitis adj5 b).ti,ab. (34)
43 hbv.ti,ab. (7)
44 hepatitis-d.ti,ab. (0)
45 (hepatitis adj5 delta).ti,ab. (0)
46 hep-d.ti,ab. (0)
47 (hepatitis adj5 d).ti,ab. (0)

© 2019 Ong JJ et al. *JAMA Network Open.*

48 (hep adj5 d).ti,ab. (0)
49 hdv.ti,ab. (5)
50 herpes genitalis.ti,ab. (0)
51 herpes virus.ti,ab. (0)
52 herpes simplex.ti,ab. (3)
53 herpesvirus.ti,ab. (0)
54 simplexvirus.ti,ab. (0)
55 "HSV-1".ti,ab. (0)
56 "HHV-1".ti,ab. (0)
57 "HSV-2".ti,ab. (2)
58 "HHV-2".ti,ab. (0)
59 human papillomavirus.ti,ab. (15)
60 genital wart*.ti,ab. (1)
61 anogenital wart*.ti,ab. (3)
62 anorectal wart*.ti,ab. (0)
63 penile wart*.ti,ab. (0)
64 condylomata acuminata.ti,ab. (0)
65 condyloma.ti,ab. (0)
66 cervical cancer.ti,ab. (44)
67 HPV.ti,ab. (28)
68 candidiasis.ti,ab. (1)
69 candida.ti,ab. (6)
70 candidal vaginitis.ti,ab. (0)
71 candidosis.ti,ab. (0)
72 vulvovaginitis.ti,ab. (0)
73 vulvitis.ti,ab. (0)
74 vulvovaginal candidiasis.ti,ab. (1)
75 vulvodynia.ti,ab. (1)
76 balanitis.ti,ab. (0)
77 monilia albicans.ti,ab. (0)
78 monilial infection.ti,ab. (0)
79 trichomoniasis.ti,ab. (4)
80 trichomoniases.ti,ab. (0)
81 trichomonas vaginalis.ti,ab. (0)
82 trichomonas vaginitides.ti,ab. (0)
83 trichomonas vaginitis.ti,ab. (0)
84 trichomonas vaginali.ti,ab. (0)
85 or/1-84 (438)
86 pre-exposure prophylaxis.ti,ab. (2)
87 preexposure prophylaxis.ti,ab. (0)
88 prep.ti,ab. (28)
89 or/86-88 (29)
90 85 and 89 (0)

© 2019 Ong JJ et al. *JAMA Network Open.*

3.6 Ebsco CINAHL Plus

Database name	CINAHL Plus
Database platform	Ebsco
Dates of database coverage	Complete database to 20/11/2018
Date searched	20/11/2018
Searched by	JF
Number of hits	197

S1 (MH "Sexually Transmitted Diseases") (11,386)
S2 (TI sexually transmitted disease*) OR (AB sexually transmitted disease*) (3,965)
S3 (TI sexually transmissible disease*) OR (AB sexually transmissible disease*) (35)
S4 (TI sexually transmitted infection*) OR (AB sexually transmitted infection*) (5,650)
S5 (TI sexually transmissible infection*) OR (AB sexually transmissible infection*) (325)
S6 (TI sexually transmitted infectious disease*) OR (AB sexually transmitted infectious disease*) (6)
S7 (TI sexually transmissible infectious disease*) OR (AB sexually transmissible infectious disease*) (0)
S8 (TI sexually transmitted disorder*) OR (AB sexually transmitted disorder*) (1)
S9 (TI sexually transmissible disorder*) OR (AB sexually transmissible disorder*) (0)
S10 (TI STI) OR (AB STI) (4,001)
S11 (TI STD) OR (AB STD) (3,342)
S12 (TI genital ulcer*) OR (AB genital ulcer*) (380)
S13 (TI genital infection*) OR (AB genital infection*) (304)
S14 (TI genital disorder*) OR (AB genital disorder*) (16)
S15 (TI venereal disease*) OR (AB venereal disease*) (290)
S16 (TI venereal infection*) OR (AB venereal infection*) (11)
S17 (TI venereal disorder*) OR (AB venereal disorder*) (1)
S18 (MH "Sexually Transmitted Diseases, Bacterial") (245)
S19 (MH "Vaginosis, Bacterial") (1,152)
S20 (MH "Gardnerella") (99)
S21 (TI bacterial vaginosis) OR (AB bacterial vaginosis) (1,002)
S22 (TI gardnerella vaginalis) OR (AB gardnerella vaginalis) (122)
S23 (TI mobiluncus) OR (AB mobiluncus) (25)
S24 (TI bacterial vaginitis) OR (AB bacterial vaginitis) (5)
S25 (MH "Chancroid") (25)
S26 (TI chancroid*) OR (AB chancroid*) (65)
S27 (TI haemophilus ducreyi) OR (AB haemophilus ducreyi) (59)
S28 (TI hemophilus ducreyi) OR (AB hemophilus ducreyi) (1)
S29 (MH "Chlamydia Infections" (3,540)
S30 (MH "Lymphogranuloma Venereum") (195)
S31 (MH "Chlamydia+") (1,785)
S32 (TI chlamydia) OR (AB chlamydia) (4,067)
S33 (TI lymphogranuloma venereum) OR (AB lymphogranuloma venereum) (156)

© 2019 Ong JJ et al. *JAMA Network Open.*

S34 (TI LGV) OR (AB LGV) (71)
S35 (TI lymphogranuloma inguinale) OR (AB lymphogranuloma inguinale) (0)
S36 (MH "Gonorrhea") (2,475)
S37 (MH "Neisseria") (960)
S38 (TI gonorrhea) OR (AB gonorrhea) (1,812)
S39 (TI neisseria gonorrhoeae) OR (AB neisseria gonorrhoeae) (1,125)
S40 (TI gonococcal urethritis) OR (AB gonococcal urethritis) (80)
S41 (TI gonococci) OR (AB gonococci) (66)
S42 (MH "Granuloma Inguinale") (29)
S43 (MH "Klebsiella") (1,274)
S44 (TI granuloma inguinale) OR (AB granuloma inguinale) (12)
S45 (TI Calymmatobacterium granulomatis) OR (AB Calymmatobacterium granulomatis) (5)
S46 (TI granuloma venereum) OR (AB granuloma venereum) (0)
S47 (TI donovanosis) OR (AB donovanosis) (22)
S48 (MH "Mycoplasma Infections") (464)
S49 (TI mycoplasma genitalium) OR (AB mycoplasma genitalium) (265)
S50 (MH "Syphilis+") (3,291)
S51 (TI syphilis) OR (AB syphilis) (3,367)
S52 (TI chancre) OR (AB chancre) (34)
S53 (TI treponema pallidum) OR (AB treponema pallidum) (378)
S54 (TI condylomata lata) OR (AB condylomata lata) (3)
S55 (MH "Sexually Transmitted Diseases, Viral") (295)
S56 (MH "Hepatitis, Viral, Human") (958)
S57 (MH "Hepatitis Viruses") (3,677)
S58 (MH "Hepatitis B+") (8,088)
S59 (TI hepatitis-b) OR (AB hepatitis-b) (8,892)
S60 (TI hep-b) OR (AB hep-b) (60)
S61 (TI hepatitis N5 b) OR (AB hepatitis N5 b) (9,192)
S62 (TI hbv) OR (AB hbv) (3,674)
S63 (MH "Hepatitis D") (194)
S64 (TI hepatitis-d) OR (AB hepatitis-d) (93)
S65 (TI hepatitis N5 delta) OR (AB hepatitis N5 delta) (87)
S66 (TI hep-d) OR (AB hep-d) (1)
S67 (TI hepatitis N5 d) OR (AB hepatitis N5 d) (201)
S68 (TI hep N5 d) OR (AB hep N5 d) (3)
S69 (TI hdv) OR (AB hdv) (110)
S70 (MH "Herpes Simplex") (1,939)
S71 (MH "Herpes Genitalis") (1,230)
S72 (MH "Herpesviruses") (2,830)
S73 (TI herpes genitalis) OR (AB herpes genitalis) (25)
S74 (TI genital herpes) OR (AB genital herpes) (614)
S75 (TI herpes virus) OR (AB herpes virus) (405)
S76 (TI herpes simplex) OR (AB herpes simplex) (2,681)
S77 (TI herpesvirus) OR (AB herpesvirus) (786)
S78 (TI simplexvirus) OR (AB simplexvirus) (0)
S79 (TI "HSV-1") OR (AB "HSV-1") (401)

© 2019 Ong JJ et al. *JAMA Network Open.*

S80 (TI "HHV-1") OR (AB "HHV-1") (6)
S81 (TI "HSV-2") OR (AB "HSV-2") (610)
S82 (TI "HHV-2") OR (AB "HHV-2") (2)
S83 (MH "Warts, Venereal") (1,029)
S84 (MH "Papillomaviruses") (3,957)
S85 (TI human papillomavirus) OR (AB human papillomavirus) (7,103)
S86 (TI genital wart*) OR (AB genital wart*) (625)
S87 (TI anogenital wart*) OR (AB anogenital wart*) (126)
S88 (TI anorectal wart*) OR (AB anorectal wart*) (1)
S89 (TI penile wart*) OR (AB penile wart*) (9)
S90 (TI condylomata acuminata) OR (AB condylomata acuminata) (68)
S91 (TI condyloma) OR (AB condyloma) (198)
S92 (TI cervical cancer) OR (AB cervical cancer) (9,528)
S93 (TI HPV) OR (AB HPV) (7,944)
S94 (MH "Sexually Transmitted Diseases, Fungal") (2)
S95 (MH "Candidiasis, Vulvovaginal") (673)
S96 (MH "Candida Albicans") (1,256)
S97 (TI candidiasis) OR (AB candidiasis) (1,774)
S98 (TI candida) OR (AB candida) (3,936)
S99 (TI candidal vaginitis) OR (AB candidal vaginitis) (13)
S100 (TI candidosis) OR (AB candidosis) (121)
S101 (TI vulvovaginitis) OR (AB vulvovaginitis) (130)
S102 (TI vulvitis) OR (AB vulvitis) (37)
S103 (TI vulvovaginal candidiasis) OR (AB vulvovaginal candidiasis) (255)
S104 (TI vulvodynia) OR (AB vulvodynia) (324)
S105 (TI balanitis) OR (AB balanitis) (92)
S106 (TI monilia albicans) OR (AB monilia albicans) (4)
S107 (TI monilial infection) OR (AB monilial infection) (1)
S108 (MH "Sexually Transmitted Diseases, Protozoal") (4)
S109 (MH "Trichomonas Vaginitis") (440)
S110 (TI trichomoniasis) OR (AB trichomoniasis) (381)
S111 (TI trichomoniases) OR (AB trichomoniases) (1)
S112 (TI trichomonas vaginalis) OR (AB trichomonas vaginalis) (562)
S113 (TI trichomonas vaginali) OR (AB trichomonas vaginali) (435)
S114 S1 OR S2 OR S3 OR S4 OR S5 OR S6 OR S7 OR S8 OR S9 OR S10 OR S11 OR S12 OR S13 OR S14 OR S15 OR S16 OR S17 OR S18 OR S19 OR S20 OR S21 OR S22 OR S23 OR S24 OR S25 OR S26 OR S27 OR S28 OR S29 OR S30 OR S31 OR S32 OR S33 OR S34 OR S35 OR S36 OR S37 OR S38 OR S39 OR S40 OR S41 OR S42 OR S43 OR S44 OR S45 OR S46 OR S47 OR S48 OR S49 OR S50 OR S51 OR S52 OR S53 OR S54 OR S55 OR S56 OR S57 OR S58 OR S59 OR S60 OR S61 OR S62 OR S63 OR S64 OR S65 OR S66 OR S67 OR S68 OR S69 OR S70 OR S71 OR S72 OR S73 OR S74 OR S75 OR S76 OR S77 OR S78 OR S79 OR S80 OR S81 OR S82 OR S83 OR S84 OR S85 OR S86 OR S87 OR S88 OR S89 OR S90 OR S91 OR S92 OR S93 OR S94 OR S95 OR S96 OR S97 OR S98 OR S99 OR S100 OR S101 OR S102 OR S103 OR S104 OR S105 OR S106 OR S107 OR S108 OR S109 OR S110 OR S111 OR S112 OR S113 (71,555)
S115 (MH "Pre-Exposure Prophylaxis") (372)
S116 (TI pre-exposure prophylaxis) OR (AB pre-exposure prophylaxis) (766)

© 2019 Ong JJ et al. *JAMA Network Open.*

S117 (TI preexposure prophylaxis) OR (AB preexposure prophylaxis) (303)
S118 (TI prep) OR (AB prep) (1,791)
S119 S115 OR S116 OR S117 OR S118 (2,274)
S120 S114 AND S119 (197)

3.7 Ebsco Africa-Wide Information

Database name	Africa-Wide Information
Database platform	Ebsco
Dates of database coverage	Complete database to 20/11/2018
Date searched	20/11/2018
Searched by	JF
Number of hits	58

S1 (TI sexually transmitted disease*) OR (AB sexually transmitted disease*) (3,808)
S2 (TI sexually transmissible disease*) OR (AB sexually transmissible disease*) (37)
S3 (TI sexually transmitted infection*) OR (AB sexually transmitted infection*) (2,762)
S4 (TI sexually transmissible infection*) OR (AB sexually transmissible infection*) (70)
S5 (TI sexually transmitted infectious disease*) OR (AB sexually transmitted infectious disease*) (6)
S6 (TI sexually transmissible infectious disease*) OR (AB sexually transmissible infectious disease*) (0)
S7 (TI sexually transmitted disorder*) OR (AB sexually transmitted disorder*) (1)
S8 (TI sexually transmissible disorder*) OR (AB sexually transmissible disorder*) (1)
S9 (TI STI) OR (AB STI) (1,599)
S10 (TI STD) OR (AB STD) (4,078)
S11 (TI genital ulcer*) OR (AB genital ulcer*) (792)
S12 (TI genital infection*) OR (AB genital infection*) (285)
S13 (TI genital disorder*) OR (AB genital disorder*) (12)
S14 (TI venereal disease*) OR (AB venereal disease*) (614)
S15 (TI venereal infection*) OR (AB venereal infection*) (10)
S16 (TI venereal disorder*) OR (AB venereal disorder*) (0)
S17 (TI bacterial vaginosis) OR (AB bacterial vaginosis) (513)
S18 (TI gardnerella vaginalis) OR (AB gardnerella vaginalis) (96)
S19 (TI mobiluncus) OR (AB mobiluncus) (8)
S20 (TI bacterial vaginitis) OR (AB bacterial vaginitis) (4)
S21 (TI chancroid*) OR (AB chancroid*) (262)
S22 (TI haemophilus ducreyi) OR (AB haemophilus ducreyi) (172)
S23 (TI hemophilus ducreyi) OR (AB hemophilus ducreyi) (11)
S24 (TI chlamydia) OR (AB chlamydia) (1,979)
S25 (TI lymphogranuloma venereum) OR (AB lymphogranuloma venereum) (111)
S26 (TI LGV) OR (AB LGV) (43)
S27 (TI lymphogranuloma inguinale) OR (AB lymphogranuloma inguinale) (3)
S28 (TI gonorrhea) OR (AB gonorrhea) (776)
S29 (TI neisseria gonorrhoeae) OR (AB neisseria gonorrhoeae) (927)
S30 (TI gonococcal urethritis) OR (AB gonococcal urethritis) (107)

© 2019 Ong JJ et al. *JAMA Network Open.*

S31 (TI gonococci) OR (AB gonococci) (96)
S32 (TI granuloma inguinale) OR (AB granuloma inguinale) (66)
S33 (TI Calymmatobacterium granulomatis) OR (AB Calymmatobacterium granulomatis) (18)
S34 (TI granuloma venereum) OR (AB granuloma venereum) (4)
S35 (TI donovanosis) OR (AB donovanosis) (56)
S36 (TI mycoplasma genitalium) OR (AB mycoplasma genitalium) (117)
S37 (TI syphilis) OR (AB syphilis) (3,146)
S38 (TI chancre) OR (AB chancre) (102)
S39 (TI treponema pallidum) OR (AB treponema pallidum) (423)
S40 (TI condylomata lata) OR (AB condylomata lata) (1)
S41 (TI hepatitis-b) OR (AB hepatitis-b) (6,503)
S42 (TI hep-b) OR (AB hep-b) (9)
S43 (TI hepatitis N5 b) OR (AB hepatitis N5 b) (6,875)
S44 (TI hbv) OR (AB hbv) (3,118)
S45 (TI hepatitis-d) OR (AB hepatitis-d) (83)
S46 (TI hepatitis N5 delta) OR (AB hepatitis N5 delta) (142)
S47 (TI hep-d) OR (AB hep-d) (0)
S48 (TI hepatitis N5 d) OR (AB hepatitis N5 d) (176)
S49 (TI hep N5 d) OR (AB hep N5 d) (2)
S50 (TI hdv) OR (AB hdv) (152)
S51 (TI herpes genitalis) OR (AB herpes genitalis) (28)
S52 (TI genital herpes) OR (AB genital herpes) (292)
S53 (TI herpes virus) OR (AB herpes virus) (382)
S54 (TI herpes simplex) OR (AB herpes simplex) (1,856)
S55 (TI herpesvirus) OR (AB herpesvirus) (1,137)
S56 (TI simplexvirus) OR (AB simplexvirus) (1)
S57 (TI "HSV-1") OR (AB "HSV-1") (365)
S58 (TI "HHV-1") OR (AB "HHV-1") (5)
S59 (TI "HSV-2") OR (AB "HSV-2") (611)
S60 (TI "HHV-2") OR (AB "HHV-2") (4)
S61 (TI human papillomavirus) OR (AB human papillomavirus) (1,842)
S62 (TI genital wart*) OR (AB genital wart*) (218)
S63 (TI anogenital wart*) OR (AB anogenital wart*) (60)
S64 (TI anorectal wart*) OR (AB anorectal wart*) (0)
S65 (TI penile wart*) OR (AB penile wart*) (4)
S66 (TI condylomata acuminata) OR (AB condylomata acuminata) (47)
S67 (TI condyloma) OR (AB condyloma) (86)
S68 (TI cervical cancer) OR (AB cervical cancer) (3,269)
S69 (TI HPV) OR (AB HPV) (2,231)
S70 (TI candidiasis) OR (AB candidiasis) (1,366)
S71 (TI candida) OR (AB candida) (3,814)
S72 (TI candidal vaginitis) OR (AB candidal vaginitis) (3)
S73 (TI candidosis) OR (AB candidosis) (110)
S74 (TI vulvovaginitis) OR (AB vulvovaginitis) (58)
S75 (TI vulvitis) OR (AB vulvitis) (12)
S76 (TI vulvovaginal candidiasis) OR (AB vulvovaginal candidiasis) (94)

© 2019 Ong JJ et al. *JAMA Network Open.*

S77 (TI vulvodynia) OR (AB vulvodynia) (15)
S78 (TI balanitis) OR (AB balanitis) (150)
S79 (TI monilia albicans) OR (AB monilia albicans) (1)
S80 (TI monilial infection) OR (AB monilial infection) (3)
S81 (TI trichomoniasis) OR (AB trichomoniasis) (437)
S82 (TI trichomoniases) OR (AB trichomoniases) (3)
S83 (TI trichomonas vaginalis) OR (AB trichomonas vaginalis) (621)
S84 (TI trichomonas vaginali) OR (AB trichomonas vaginali) (184)
S85 (TI pre-exposure prophylaxis) OR (AB pre-exposure prophylaxis) (330)
S86 (TI preexposure prophylaxis) OR (AB preexposure prophylaxis) (87)
S87 (TI prep) OR (AB prep) (603)
S88 S1 OR S2 OR S3 OR S4 OR S5 OR S6 OR S7 OR S8 OR S9 OR S10 OR S11 OR S12 OR S13 OR S14 OR S15 OR S16 OR S17 OR S18 OR S19 OR S20 OR S21 OR S22 OR S23 OR S24 OR S25 OR S26 OR S27 OR S28 OR S29 OR S30 OR S31 OR S32 OR S33 OR S34 OR S35 OR S36 OR S37 OR S38 OR S39 OR S40 OR S41 OR S42 OR S43 OR S44 OR S45 OR S46 OR S47 OR S48 OR S49 OR S50 OR S51 OR S52 OR S53 OR S54 OR S55 OR S56 OR S57 OR S58 OR S59 OR S60 OR S61 OR S62 OR S63 OR S64 OR S65 OR S66 OR S67 OR S68 OR S69 OR S70 OR S71 OR S72 OR S73 OR S74 OR S75 OR S76 OR S77 OR S78 OR S79 OR S80 OR S81 OR S82 OR S83 OR S84 (34,702)
S89 S85 OR S86 OR S87 (744)
S90 S88 AND S89 (58)

3.8 Web of Science Core Collection

Database name	Web of Science Core Collection
Database platform	Web of Science
Dates of database coverage	• Science Citation Index Expanded (SCI-EXPANDED) --1970-present<br>• Social Sciences Citation Index (SSCI) --1970-present<br>• Arts & Humanities Citation Index (A&HCI) --1975-present<br>• Conference Proceedings Citation Index-Science (CPCI-S) --1990-present<br>• Conference Proceedings Citation Index- Social Science & Humanities (CPCI-SSH) --1990-present<br>• Emerging Sources Citation Index (ESCI) --2015-present<br><br>Data last updated: 2018-11-19
Date searched	20/11/2018
Searched by	JF
Number of hits	677

© 2019 Ong JJ et al. *JAMA Network Open.*

1 TOPIC: ("sexually transmitted disease*" OR "sexually transmissible disease*" OR "sexually transmitted infection*" OR "sexually transmissible infection*" OR "sexually transmitted infectious disease*" OR "sexually transmissible infectious disease*" OR "sexually transmitted disorder*" OR "sexually transmissible disorder*" OR STI OR STD OR "genital ulcer*" OR "genital infection*" OR "genital disorder*" OR "venereal disease*" OR "venereal infection*" OR "venereal disorder*") (49,744)
2 TOPIC: ("bacterial vaginosis" OR "gardnerella vaginalis" OR mobiluncus OR "bacterial vaginitis" OR chancroid* OR "haemophilus ducreyi" OR chlamydia OR "lymphogranuloma venereum" OR LGV OR "lymphogranuloma inguinale" OR gonorrhea OR "neisseria gonorrhoeae" OR "gonococcal urethritis" OR gonococci OR "granuloma inguinale" OR "Calymmatobacterium granulomatis" OR "granuloma venereum" OR donovanosis OR "mycoplasma genitalium" OR syphilis OR chancre OR "treponema pallidum" OR "condylomata lata") (61,141)
3 261,284 TOPIC: ("hepatitis-b" OR "hep-b" OR (hepatitis NEAR/5 b) OR (hep NEAR/5 b) OR hbv OR "hepatitis-d" OR (hepatitis NEAR/5 delta) OR "hep-d" OR (hepatitis NEAR/5 d) OR (hep NEAR/5 d) OR hdv OR "herpes genitalis" OR "herpes virus" OR "herpes simplex" OR herpesvirus OR simplexvirus OR "HSV-1" OR "HHV-1" OR "HSV-2" OR "HHV-2" OR "human papillomavirus" OR "genital wart*" OR "anogenital wart*" OR "anorectal wart*" OR "penile wart*" OR "venereal wart*" OR "condylomata acuminata" OR condyloma OR "cervical cancer" OR HPV) (261,284)
4 TOPIC: (candidiasis OR candida OR "candidal vaginitis" OR candidosis OR vulvovaginitis OR vulvitis OR "vulvovaginal candidiasis" OR vulvodynia OR balanitis OR "monilia albicans" OR "monilial infection") (87,540)
5 TOPIC: (trichomoniasis OR trichomoniases OR "trichomonas vaginalis" OR "trichomonas vaginitides" OR "trichomonas vaginitis" OR "trichomonas vaginali") (5,594)
6 #5 OR #4 OR #3 OR #2 OR #1 (440,484)
7 TOPIC: ("pre-exposure prophylaxis" OR "preexposure prophylaxis" OR prep) (7,074)
8 #7 AND #6 (677)

3.9 VHL LILACS

Database name	LILACS
Database platform	VHL
Dates of database coverage	complete database to 20/11/2018
Date searched	20/11/2018
Searched by	JF
Number of hits	1

("sexually transmitted disease$" OR "sexually transmissible disease$" OR "sexually transmitted infection$" OR "sexually transmissible infection$" OR "sexually transmitted infectious disease$" OR "sexually transmissible infectious disease$" OR "sexually transmitted disorder$" OR "sexually transmissible disorder$" OR STI OR STD OR "genital ulcer$" OR "genital infection$" OR "genital disorder$" OR "venereal disease$" OR "venereal infection$" OR "venereal disorder$" OR "bacterial vaginosis" OR "gardnerella vaginalis" OR mobiluncus OR "bacterial vaginitis" OR chancroid$ OR "haemophilus ducreyi" OR chlamydia

© 2019 Ong JJ et al. *JAMA Network Open.*

OR "lymphogranuloma venereum" OR LGV OR "lymphogranuloma inguinale" OR gonorrhea OR "neisseria gonorrhoeae" OR "gonococcal urethritis" OR gonococci OR "granuloma inguinale" OR "Calymmatobacterium granulomatis" OR "granuloma venereum" OR donovanosis OR "mycoplasma genitalium" OR syphilis OR chancre OR "treponema pallidum" OR "condylomata lata" OR "hepatitis-b" OR "hep-b" OR hbv OR "hepatitis-d" OR "hepatitis delta" OR "hep-d" OR hdv OR "herpes genitalis" OR "herpes virus" OR "herpes simplex" OR herpesvirus OR simplexvirus OR "HSV-1" OR "HHV-1" OR "HSV-2" OR "HHV-2" OR "human papillomavirus" OR "genital wart$" OR "anogenital wart$" OR "anorectal wart$" OR "penile wart$" OR "venereal wart$" OR "condylomata acuminata" OR condyloma OR "cervical cancer" OR HPV OR candidiasis OR candida OR "candidal vaginitis" OR candidosis OR vulvovaginitis OR vulvitis OR "vulvovaginal candidiasis" OR vulvodynia OR balanitis OR "monilia albicans" OR "monilial infection" OR trichomoniasis OR trichomoniases OR "trichomonas vaginalis" OR "trichomonas vaginitides" OR "trichomonas vaginitis" OR "trichomonas vaginali") [Words] and ("pre-exposure prophylaxis" OR "preexposure prophylaxis" OR prep) [Words] (1)

3.10 Northern Light Life Sciences Conference Abstracts

Database name	Northern Light Life Sciences Conference Abstracts
Database platform	OvidSP
Dates of database coverage	2010 - 2018 Week 45
Date searched	20/11/2018
Searched by	JF
Number of hits	119

1 sexually transmitted disease*.ti,ab. (326)
2 sexually transmissible disease*.ti,ab. (0)
3 sexually transmitted infection*.ti,ab. (719)
4 sexually transmissible infection*.ti,ab. (12)
5 sexually transmitted infectious disease*.ti,ab. (0)
6 sexually transmissible infectious disease*.ti,ab. (0)
7 sexually transmitted disorder*.ti,ab. (0)
8 sexually transmissible disorder*.ti,ab. (0)
9 STI.ti,ab. (1716)
10 STD.ti,ab. (1276)
11 genital ulcer*.ti,ab. (82)
12 genital infection*.ti,ab. (93)
13 genital disorder*.ti,ab. (1)
14 venereal disease*.ti,ab. (19)
15 venereal infection*.ti,ab. (0)
16 venereal disorder*.ti,ab. (0)
17 bacterial vaginosis.ti,ab. (139)
18 gardnerella vaginalis.ti,ab. (34)
19 mobiluncus.ti,ab. (1)

© 2019 Ong JJ et al. *JAMA Network Open.*

20 bacterial vaginitis.ti,ab. (0)
21 chancroid?.ti,ab. (6)
22 haemophilus ducreyi.ti,ab. (17)
23 chlamydia.ti,ab. (1332)
24 lymphogranuloma venereum.ti,ab. (44)
25 LGV.ti,ab. (60)
26 lymphogranuloma inguinale.ti,ab. (0)
27 gonorrhea.ti,ab. (387)
28 neisseria gonorrhoeae.ti,ab. (403)
29 gonococcal urethritis.ti,ab. (21)
30 gonococci.ti,ab. (15)
31 granuloma inguinale.ti,ab. (1)
32 Calymmatobacterium granulomatis.ti,ab. (0)
33 granuloma venereum.ti,ab. (2)
34 donovanosis.ti,ab. (3)
35 mycoplasma genitalium.ti,ab. (131)
36 syphilis.ti,ab. (1357)
37 chancre.ti,ab. (14)
38 treponema pallidum.ti,ab. (94)
39 condylomata lata.ti,ab. (1)
40 hepatitis-b.ti,ab. (7082)
41 hep-b.ti,ab. (49)
42 (hepatitis adj5 b).ti,ab. (7291)
43 hbv.ti,ab. (6443)
44 hepatitis-d.ti,ab. (80)
45 (hepatitis adj5 delta).ti,ab. (186)
46 hep-d.ti,ab. (0)
47 (hepatitis adj5 d).ti,ab. (162)
48 (hep adj5 d).ti,ab. (2)
49 hdv.ti,ab. (329)
50 herpes genitalis.ti,ab. (3)
51 herpes virus.ti,ab. (293)
52 herpes simplex.ti,ab. (1183)
53 herpesvirus.ti,ab. (521)
54 simplexvirus.ti,ab. (0)
55 "HSV-1".ti,ab. (692)
56 "HHV-1".ti,ab. (3)
57 "HSV-2".ti,ab. (335)
58 "HHV-2".ti,ab. (1)
59 human papillomavirus.ti,ab. (1650)
60 genital wart*.ti,ab. (106)
61 anogenital wart*.ti,ab. (18)
62 anorectal wart*.ti,ab. (1)
63 penile wart*.ti,ab. (1)

© 2019 Ong JJ et al. *JAMA Network Open.*

64 condylomata acuminata.ti,ab. (7)
65 condyloma.ti,ab. (43)
66 cervical cancer.ti,ab. (5261)
67 HPV.ti,ab. (5797)
68 candidiasis.ti,ab. (717)
69 candida.ti,ab. (2534)
70 candidal vaginitis.ti,ab. (0)
71 candidosis.ti,ab. (14)
72 vulvovaginitis.ti,ab. (49)
73 vulvitis.ti,ab. (11)
74 vulvovaginal candidiasis.ti,ab. (49)
75 vulvodynia.ti,ab. (77)
76 balanitis.ti,ab. (26)
77 monilia albicans.ti,ab. (0)
78 monilial infection.ti,ab. (0)
79 trichomoniasis.ti,ab. (58)
80 trichomoniases.ti,ab. (0)
81 trichomonas vaginalis.ti,ab. (164)
82 trichomonas vaginitides.ti,ab. (0)
83 trichomonas vaginitis.ti,ab. (1)
84 trichomonas vaginali.ti,ab. (0)
85 or/1-84 (32512)
86 pre-exposure prophylaxis.ti,ab. (481)
87 preexposure prophylaxis.ti,ab. (32)
88 prep.ti,ab. (1644)
89 or/86-88 (1725)
90 85 and 89 (119)

© 2019 Ong JJ et al. *JAMA Network Open.*

eAppendix 2. Data Variables Used in Data Extraction

- Author
- Title
- Year of data
- Study type
- Total sample size of PrEP users
- Country
- Eligibility for PrEP
- Study population
    - Age
    - % Female
    - % MSM
    - % Sex worker
    - % Transgender
    - % Young people (age <25 years)
    - % Serodiscordant couples
    - % Heterosexuals
- Diagnostic methods for STIs
- STI prevalence at baseline (by pathogen, and any aggregated measures e.g. ‘bacterial STIs)
- STI incidence (by pathogen, and any aggregated measures)

© 2019 Ong JJ et al. *JAMA Network Open.*

eAppendix 3. Forest Plots by Pathogen and Subgroups

This appendix contains the Forest plots by pathogen and subgroups. For figures with multiple rows for the same reference, these estimates arise from the same study but are reporting estimates from different anatomical sites. Incidence rates are reported as per 100 person-years. If there are blanks in the plots for cases and person years of follow-up, this is because we only had the reported incidence rate and 95% confidence intervals from the study or correspondence with the author. If there are 0 cases reported, we recorded 0.5 cases and added 0.5 to its person-years of follow up. Each study is referenced by the first author, study site and year(s) of data collection.

eFigure 1, panel A – Random effects meta-analysis of chlamydia prevalence

eFigure 1, panel B – Random effects meta-analysis of chlamydia prevalence by anatomical site

eFigure 1, panel C – Random effects meta-analysis of chlamydia prevalence by MSM status of the study population

eFigure 1, panel D – Random effects meta-analysis of chlamydia prevalence by country income level

eFigure 1, panel E – Random effects meta-analysis of chlamydia prevalence by study type

eFigure 1, Panel F – Random effects meta-analysis of chlamydia prevalence by publication status

eTable 1 Meta-regression results for the predictors of chlamydia prevalence and sources of between-study heterogeneity

eFigure 1, panel G – Funnel plot and Egger’s test for chlamydia prevalence

eFigure 2, panel A – Random effects meta-analysis of gonorrhoea prevalence

eFigure 2, panel B – Random effects meta-analysis of gonorrhoea prevalence by anatomical site

© 2019 Ong JJ et al. *JAMA Network Open.*

eFigure 2, panel C – Random effects meta-analysis of gonorrhoea prevalence by MSM status of the study population

eFigure 2, panel D – Random effects meta-analysis of gonorrhoea prevalence by country income level

eFigure 2, panel E – Random effects meta-analysis of gonorrhoea prevalence by study type

eFigure 2, panel F – Random effects meta-analysis of gonorrhoea prevalence by publication status

eTable 2 Meta-regression results for the predictors of gonorrhoea prevalence and sources of between-study heterogeneity

eFigure 2, panel G - Funnel plot and Egger's test for gonorrhoea prevalence

eFigure 3, panel A- Random effects meta-analysis of early syphilis prevalence

eFigure 3, panel B - Random effects meta-analysis of early syphilis prevalence by MSM status of the study population

eFigure 3, panel C - Random effects meta-analysis of early syphilis prevalence by country income level

eFigure 3, panel D - Random effects meta-analysis of early syphilis prevalence by study type

eFigure 3, panel E - Random effects meta-analysis of early syphilis prevalence by publication status

eTable 3 - Meta-regression results for the predictors of early syphilis prevalence and sources of between-study heterogeneity

eFigure 3, panel F – Funnel plot and Egger's test of early syphilis prevalence

eFigure 4, panel A - Random effects meta-analysis of any chlamydia, gonorrhoea or early syphilis prevalence

eFigure 4, panel B - Random effects meta-analysis of any chlamydia, gonorrhoea or early syphilis prevalence by MSM status of the study population

eFigure 4, panel C - Random effects meta-analysis of any chlamydia, gonorrhoea or early syphilis prevalence by study type

eFigure 4, panel D - Random effects meta-analysis of any chlamydia, gonorrhoea or early syphilis prevalence by publication status

© 2019 Ong JJ et al. *JAMA Network Open.*

eTable 4 - Meta-regression results for the predictors of any chlamydia, gonorrhoea or early syphilis prevalence and sources of between-study heterogeneity

eFigure 4, panel E - Funnel plot and Egger's test any chlamydia, gonorrhoea or early syphilis prevalence

eFigure 5, panel A - Random effects meta-analysis of Hepatitis B prevalence

eFigure 5, panel B - Random effects meta-analysis of Hepatitis B prevalence by MSM status of the study population

eFigure 5, panel C - Random effects meta-analysis of Hepatitis B prevalence by country income level

eFigure 5, panel D - Random effects meta-analysis of Hepatitis B prevalence by study type

eFigure 5, panel E - Funnel plot and Egger's test for Hepatitis B prevalence

eFigure 6, panel A - Random effects meta-analysis of Hepatitis C prevalence

eFigure 6, panel B - Random effects meta-analysis of Hepatitis C prevalence by MSM status

eFigure 6, panel C - Funnel plot and Egger's test for Hepatitis C prevalence

eFigure 7, panel A - Random effects meta-analysis of chlamydia incidence

eFigure 7, panel B - Random effects meta-analysis of chlamydia incidence by anatomical site

eFigure 7, panel C - Random effects meta-analysis of chlamydia incidence by MSM status of the study population

eFigure 7, panel D - Random effects meta-analysis of chlamydia incidence by country income level

eFigure 7, panel E - Random effects meta-analysis of chlamydia incidence by study type

eFigure 7, panel F - Random effects meta-analysis of chlamydia incidence by publication status

eTable 5 - Meta-regression results for the predictors of chlamydia incidence and sources of between-study heterogeneity

eFigure 7, panel G - Funnel plot and Egger's for chlamydia incidence

© 2019 Ong JJ et al. *JAMA Network Open.*

eFigure 8, panel A - Random effects meta-analysis of gonorrhoea incidence

eFigure 8, panel B - Random effects meta-analysis of gonorrhoea incidence by anatomical site

eFigure 8, panel C - Random effects meta-analysis of gonorrhoea incidence by MSM status of the study population

eFigure 8, panel D - Random effects meta-analysis of gonorrhoea incidence by country income level

eFigure 8, panel E - Random effects meta-analysis of gonorrhoea incidence by study type

eFigure 8, panel F - Random effects meta-analysis of gonorrhoea incidence by publication status

eTable 6 - Meta-regression results for the predictors of gonorrhoea incidence and sources of between-study heterogeneity

eFigure 8, panel G – Funnel plot and Egger's test of gonorrhoea incidence

eFigure 9, panel A - Random effects meta-analysis of early syphilis incidence

eFigure 9, panel B - Random effects meta-analysis of early syphilis incidence by MSM status of the study population

eFigure 9, panel C - Random effects meta-analysis of early syphilis incidence by country income level

eFigure 9, panel D - Random effects meta-analysis of early syphilis incidence by study type

eFigure 9, panel E - Random effects meta-analysis of early syphilis incidence by publication status

eTable 7 - Meta-regression results for the predictors of early syphilis incidence and sources of between-study heterogeneity

eFigure 9, panel F – Funnel plot and Egger's test for early syphilis incidence

eFigure 10, panel A - Random effects meta-analysis of any chlamydia, gonorrhoea or early syphilis incidence

© 2019 Ong JJ et al. *JAMA Network Open.*

eFigure 10, panel B - Random effects meta-analysis of any chlamydia, gonorrhoea or early syphilis incidence by MSM status of the study population

eFigure 10, panel C - Random effects meta-analysis of any chlamydia, gonorrhoea or early syphilis incidence by country income level

eFigure 10, panel D - Random effects meta-analysis of any chlamydia, gonorrhoea or early syphilis incidence by study type

eFigure 10, panel E - Random effects meta-analysis of any chlamydia, gonorrhoea or early syphilis incidence by publication status

eFigure 10, panel F Funnel plot and Egger's test for any chlamydia, gonorrhoea or early syphilis incidence

eFigure 11, panel A - Random effects meta-analysis of Hepatitis C incidence

eFigure 11, panel B - Random effects meta-analysis of Hepatitis C incidence by MSM status of the study population

eFigure 11, panel C - Random effects meta-analysis of Hepatitis C incidence by country income level

eFigure 11, panel D Funnel plot and Egger's test for Hepatitis C incidence

© 2019 Ong JJ et al. *JAMA Network Open.*

eFigure 1. Random Effects Meta-Analysis of Chlamydia Prevalence
panel A – Random effects meta-analysis of chlamydia prevalence

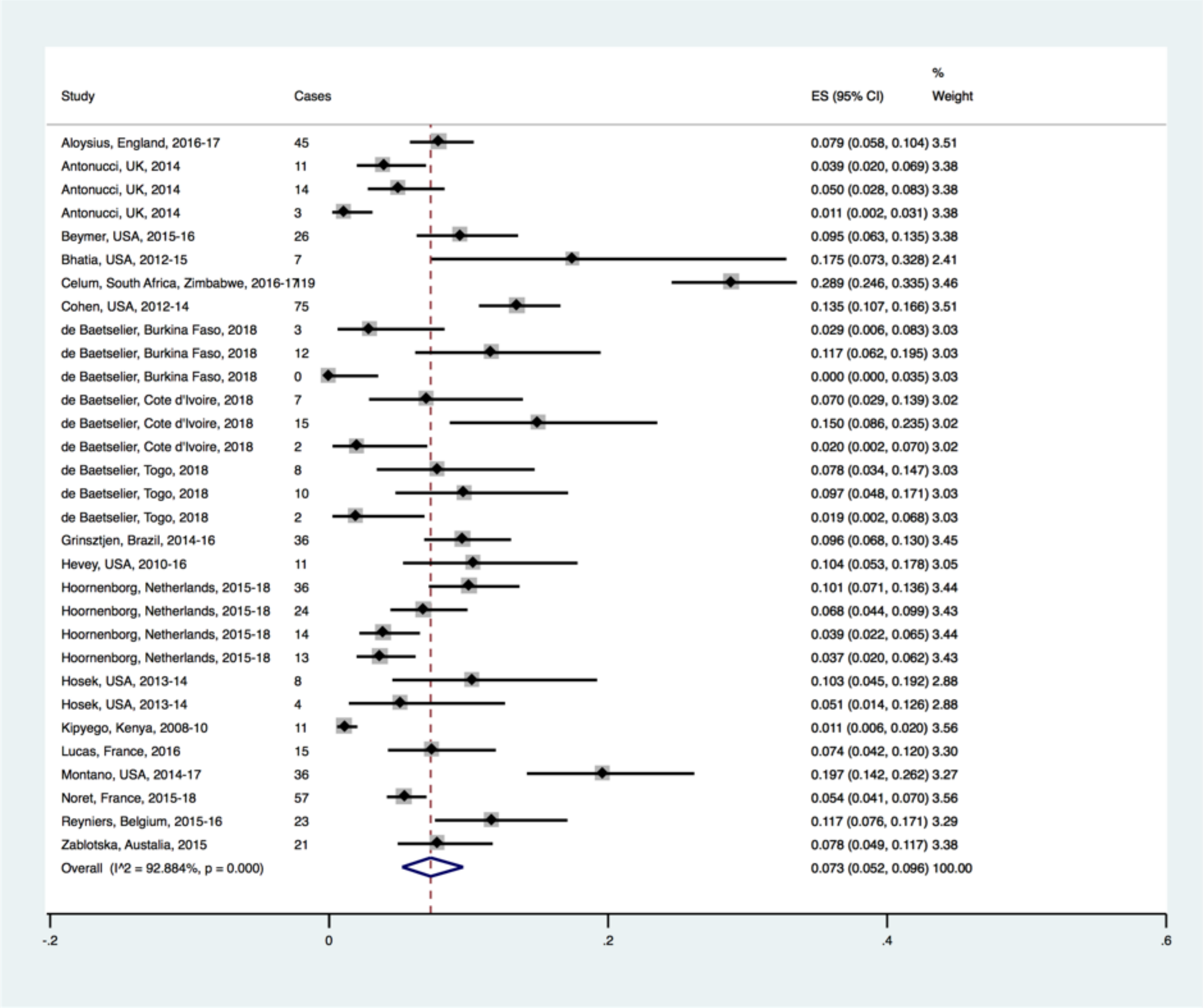

© 2019 Ong JJ et al. *JAMA Network Open.*

eFigure 1, panel B – Random effects meta-analysis of chlamydia prevalence by anatomical site

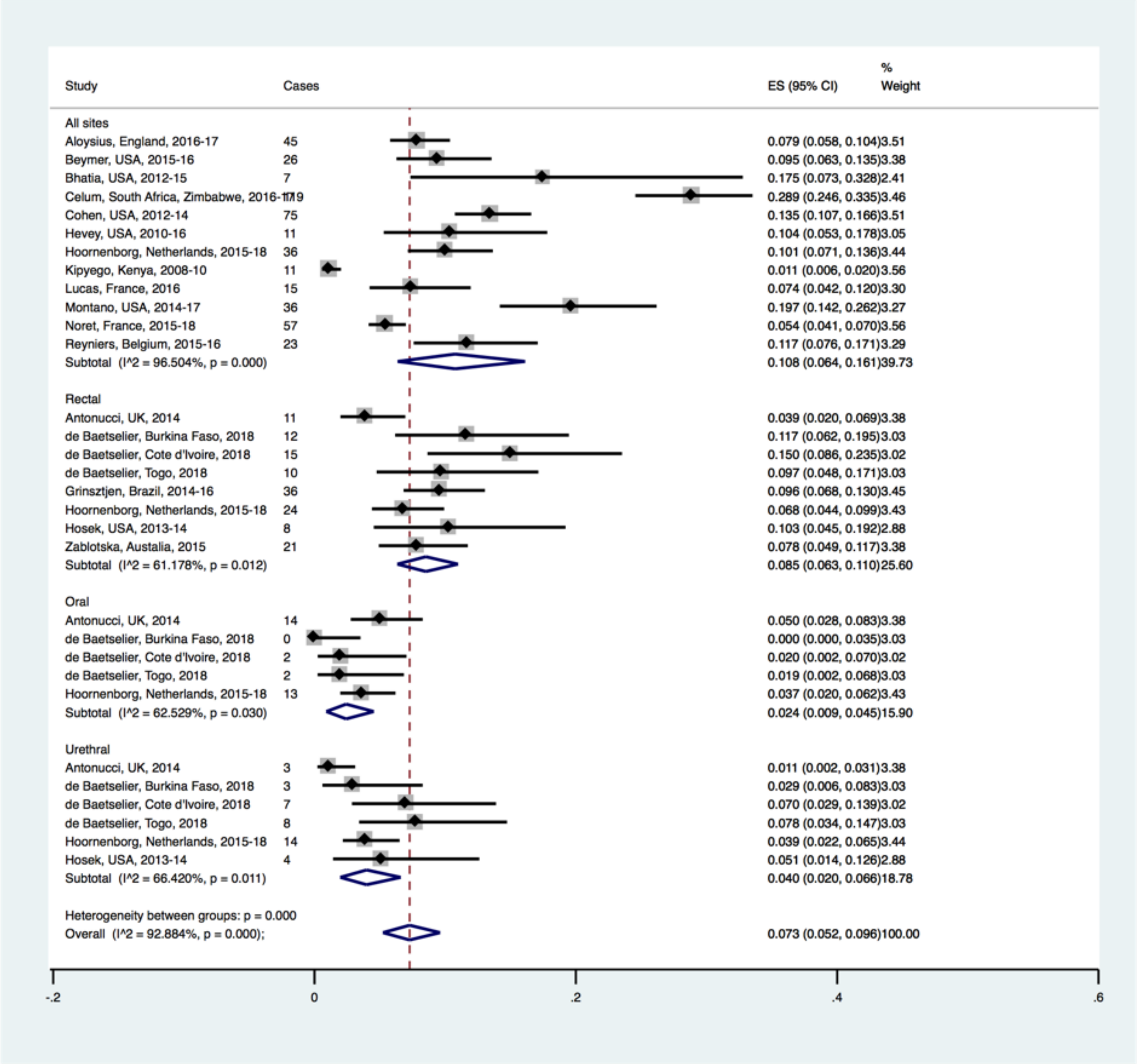

© 2019 Ong JJ et al. *JAMA Network Open.*

eFigure 1, panel C – Random effects meta-analysis of chlamydia prevalence by MSM status of the study population

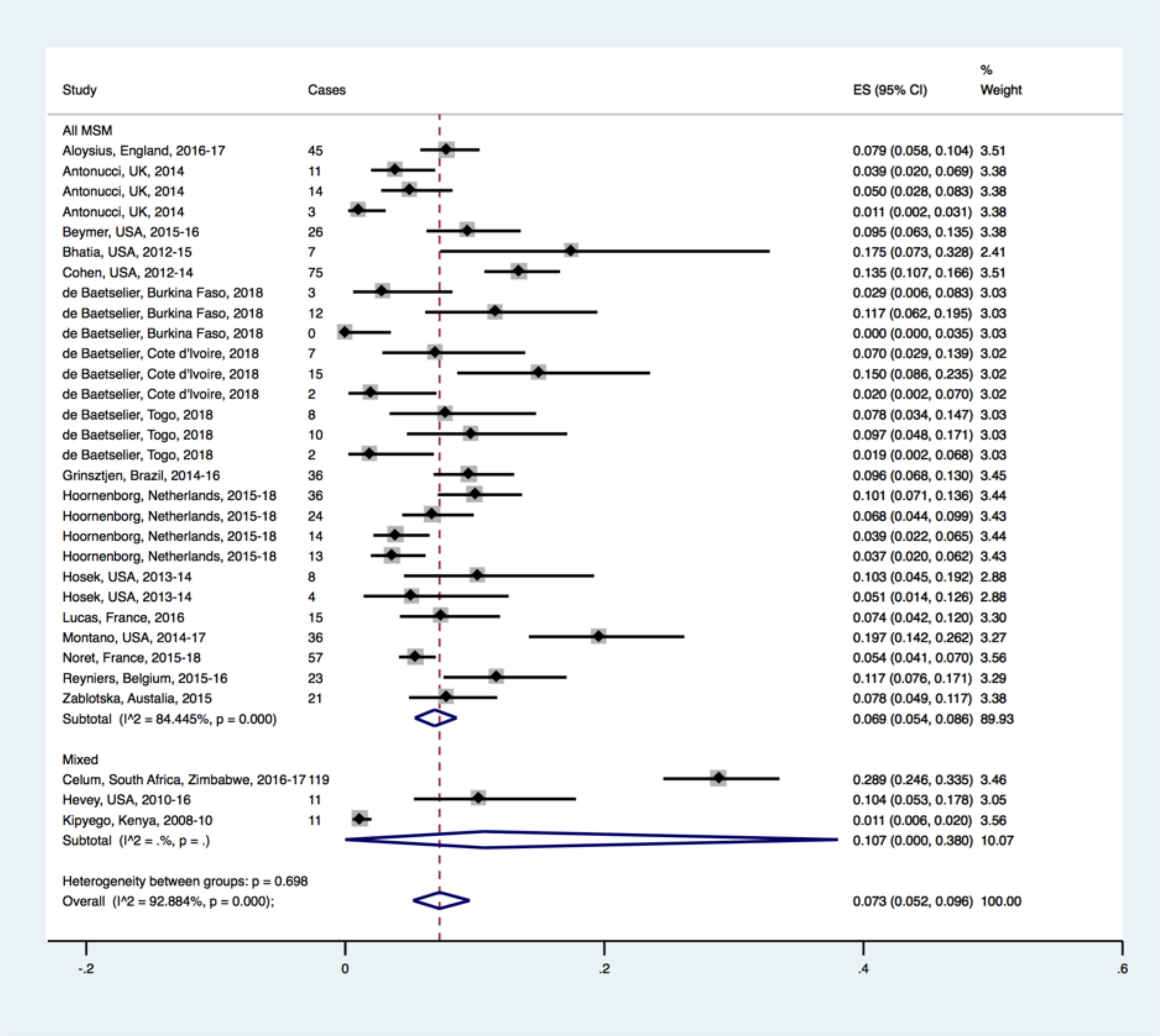

© 2019 Ong JJ et al. *JAMA Network Open.*

eFigure 1, panel D – Random effects meta-analysis of chlamydia prevalence by country income level

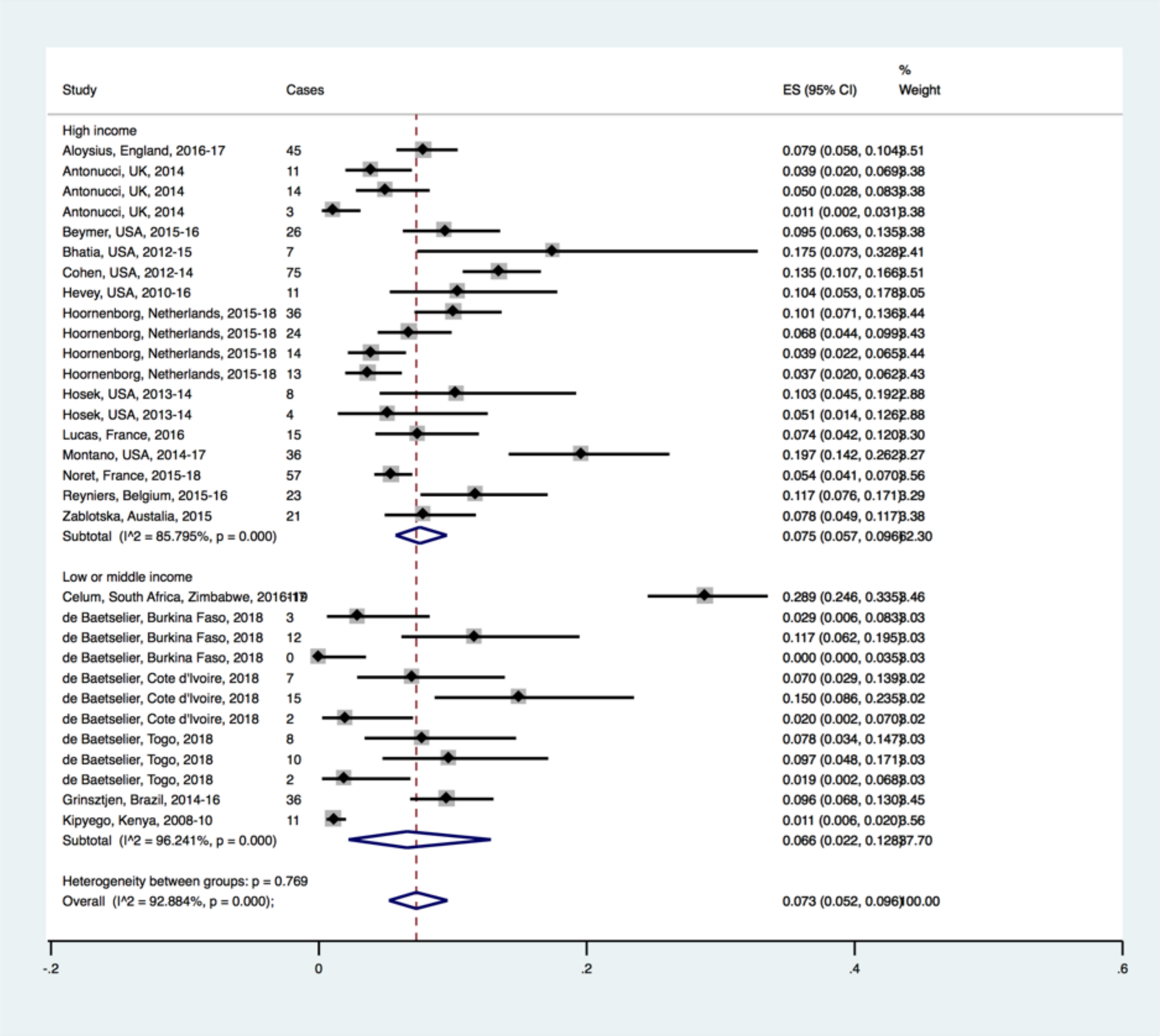

© 2019 Ong JJ et al. *JAMA Network Open.*

eFigure 1, panel E – Random effects meta-analysis of chlamydia prevalence by study type

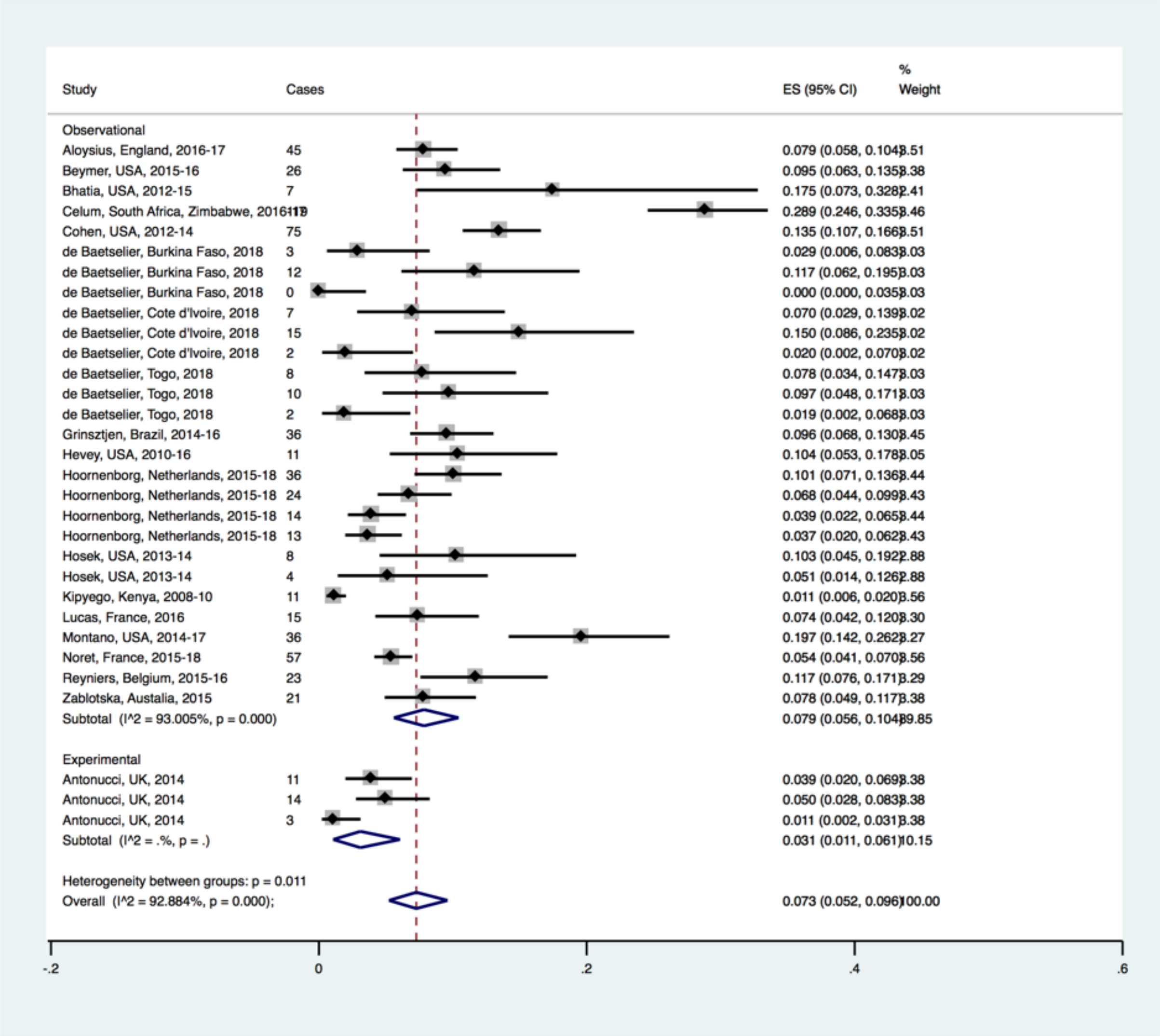

© 2019 Ong JJ et al. *JAMA Network Open.*

eFigure 1, Panel F – Random effects meta-analysis of chlamydia prevalence by publication status

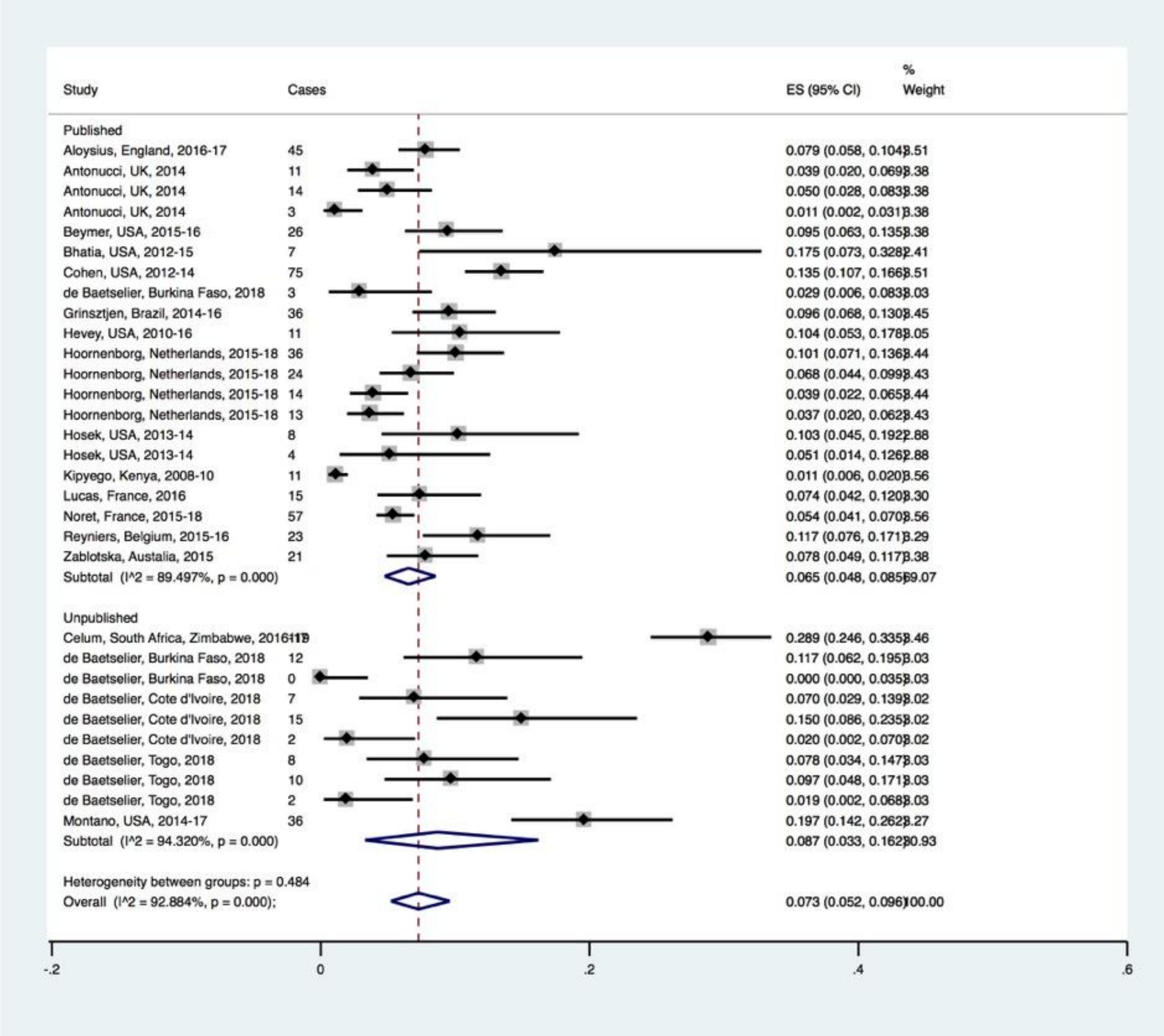

© 2019 Ong JJ et al. *JAMA Network Open.*

eTable 1. Meta-regression Results for the Predictors of Chlamydia Prevalence and Sources of Between-Study Heterogeneity

Characteristic	Variable	OR (95% CI)	p value	AOR (95% CI)	p value
Anatomical site	Oral	1		1	
	All sites	1.05 (0.99-1.11)	0.09	1.07 (0.96-1.20)	0.20
	Genital	0.95 (0.88-1.03)	0.22	1.01 (0.90-1.13)	0.87
	Anorectal	1.00 (0.94-1.07)	0.91	1.05 (0.95-1.17)	0.31
Population	Mixed	1		1	
	MSM only	0.96 (0.88-1.04)	0.29	0.99 (0.88-1.12)	0.90
Country income level	LMIC	1		1	
	High	0.99 (0.93-1.05)	0.66	0.99 (0.90-1.08)	0.73
Study type	Observational	1		1	
	Experimental	0.95 (0.87-1.03)	0.22	0.98 (0.88-1.10)	0.76

AOR = adjusted odds ratio; LMIC = low-middle income country; MSM = men who have sex with men; OR = odds ratio; RCT = randomized controlled trial

© 2019 Ong JJ et al. *JAMA Network Open.*

eFigure 1, panel G – Funnel plot and Egger’s test for chlamydia prevalence

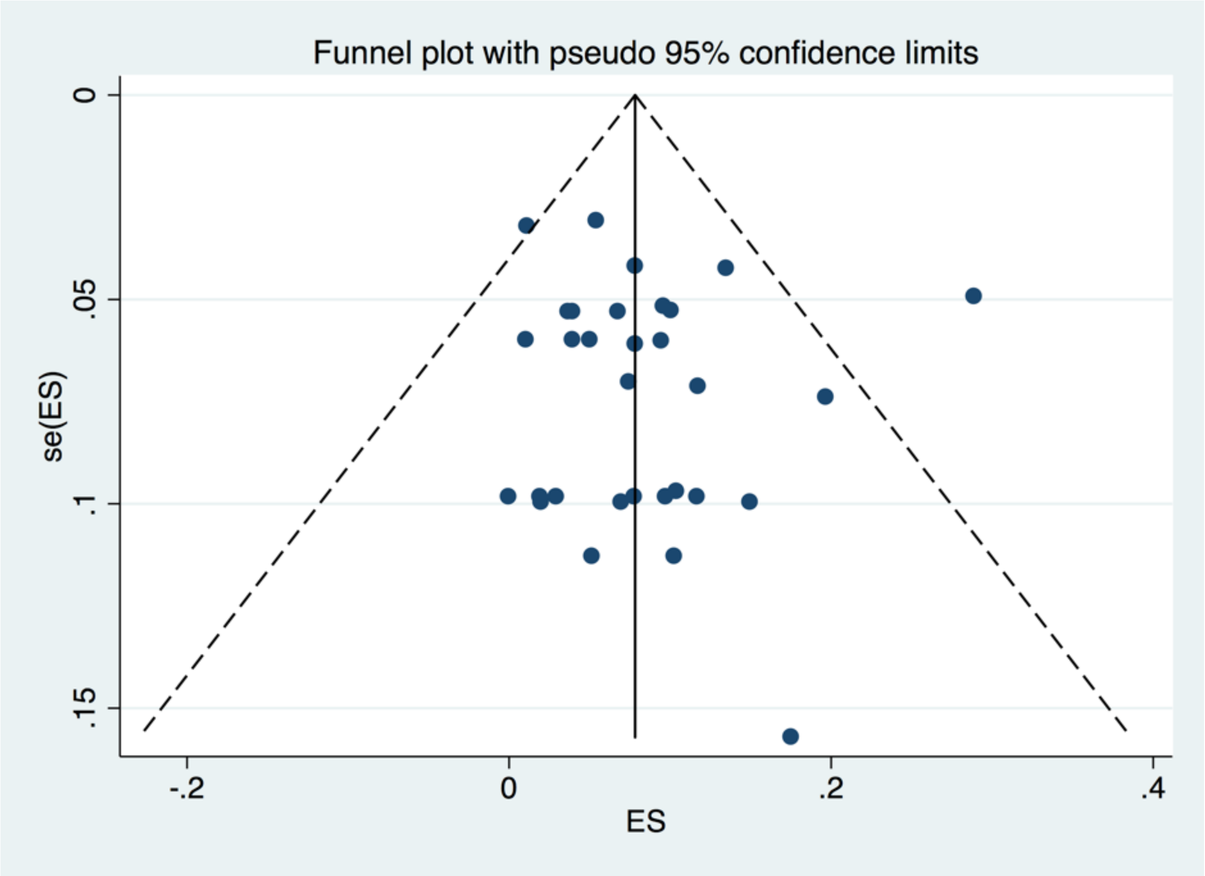

Egger’s test = 0.30

© 2019 Ong JJ et al. *JAMA Network Open.*

eFigure 2. Random Effects Meta-Analysis of Gonorrhea Prevalence
panel A – Random effects meta-analysis of gonorrhoea prevalence

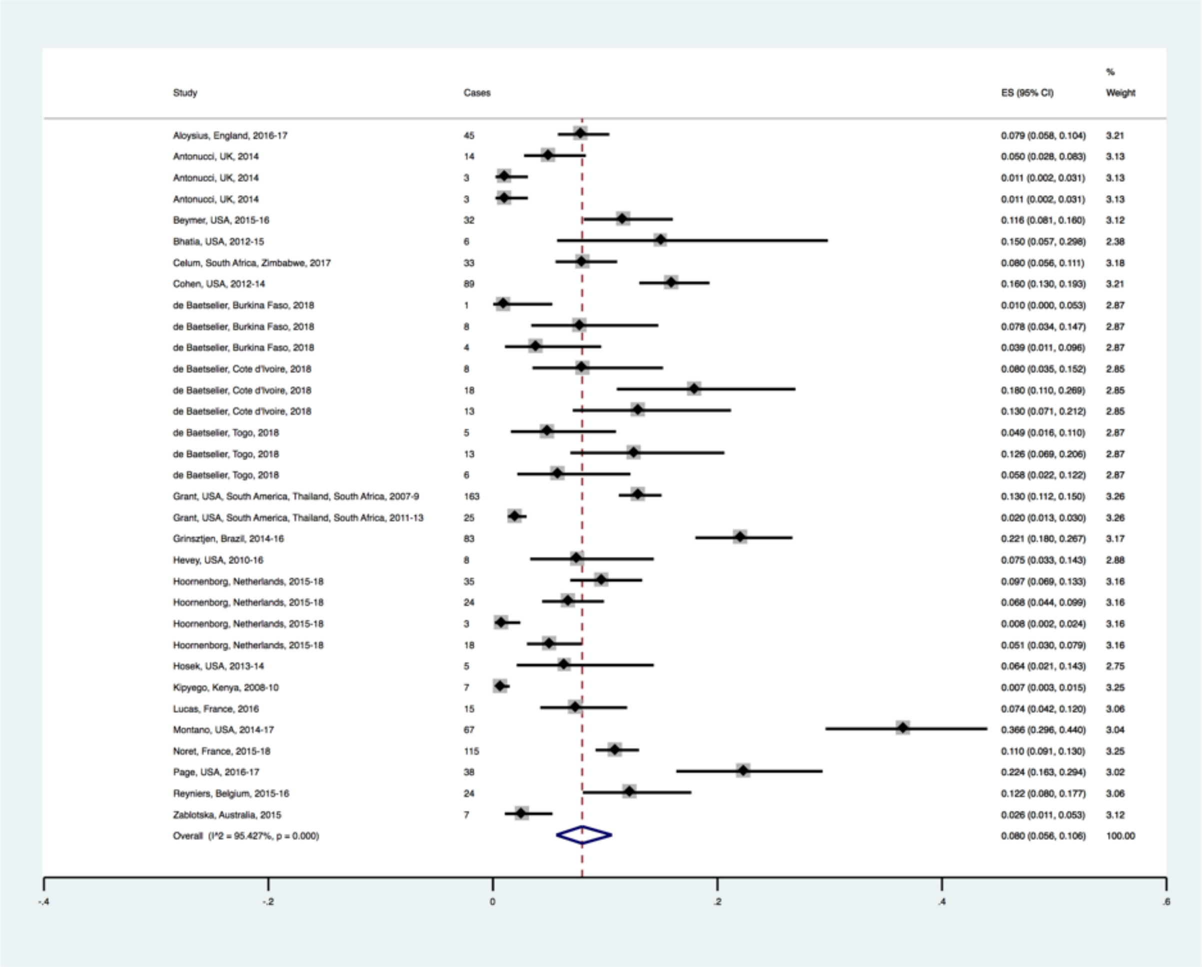

© 2019 Ong JJ et al. *JAMA Network Open.*

eFigure 2, panel B – Random effects meta-analysis of gonorrhoea prevalence by anatomical site

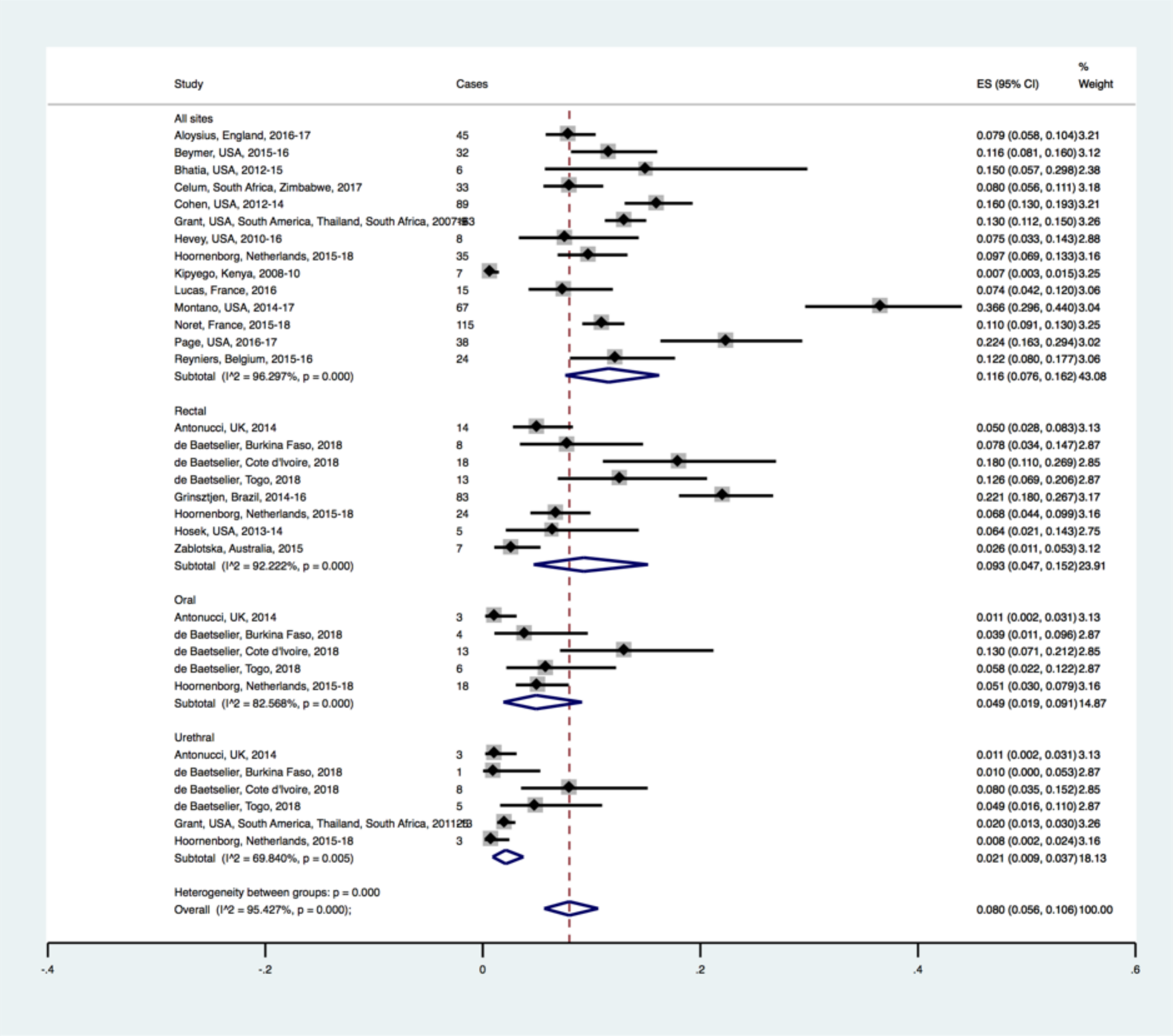

© 2019 Ong JJ et al. *JAMA Network Open.*

eFigure 2, panel C – Random effects meta-analysis of gonorrhoea prevalence by MSM status of the study population

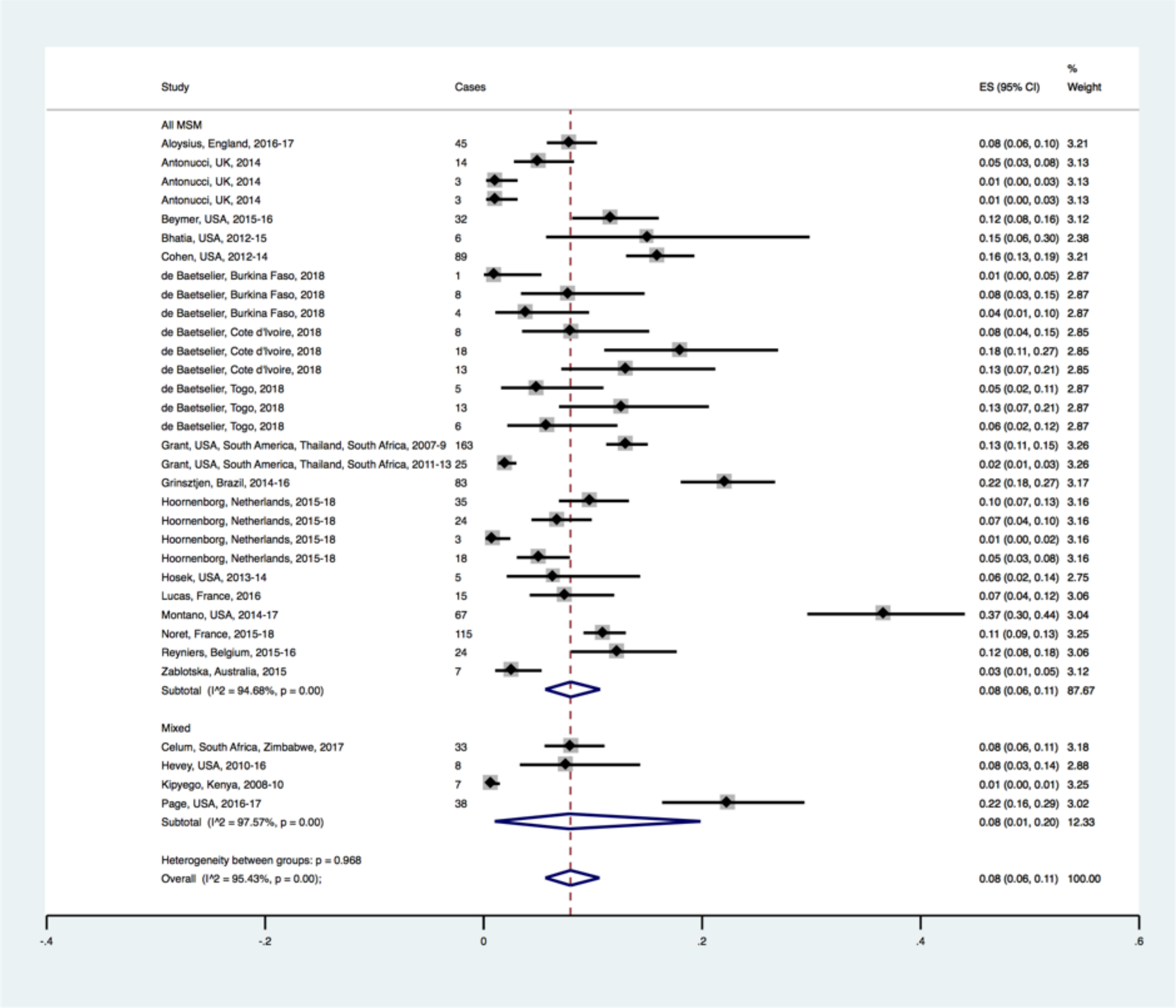

© 2019 Ong JJ et al. *JAMA Network Open.*

eFigure 2, panel D – Random effects meta-analysis of gonorrhoea prevalence by country income level

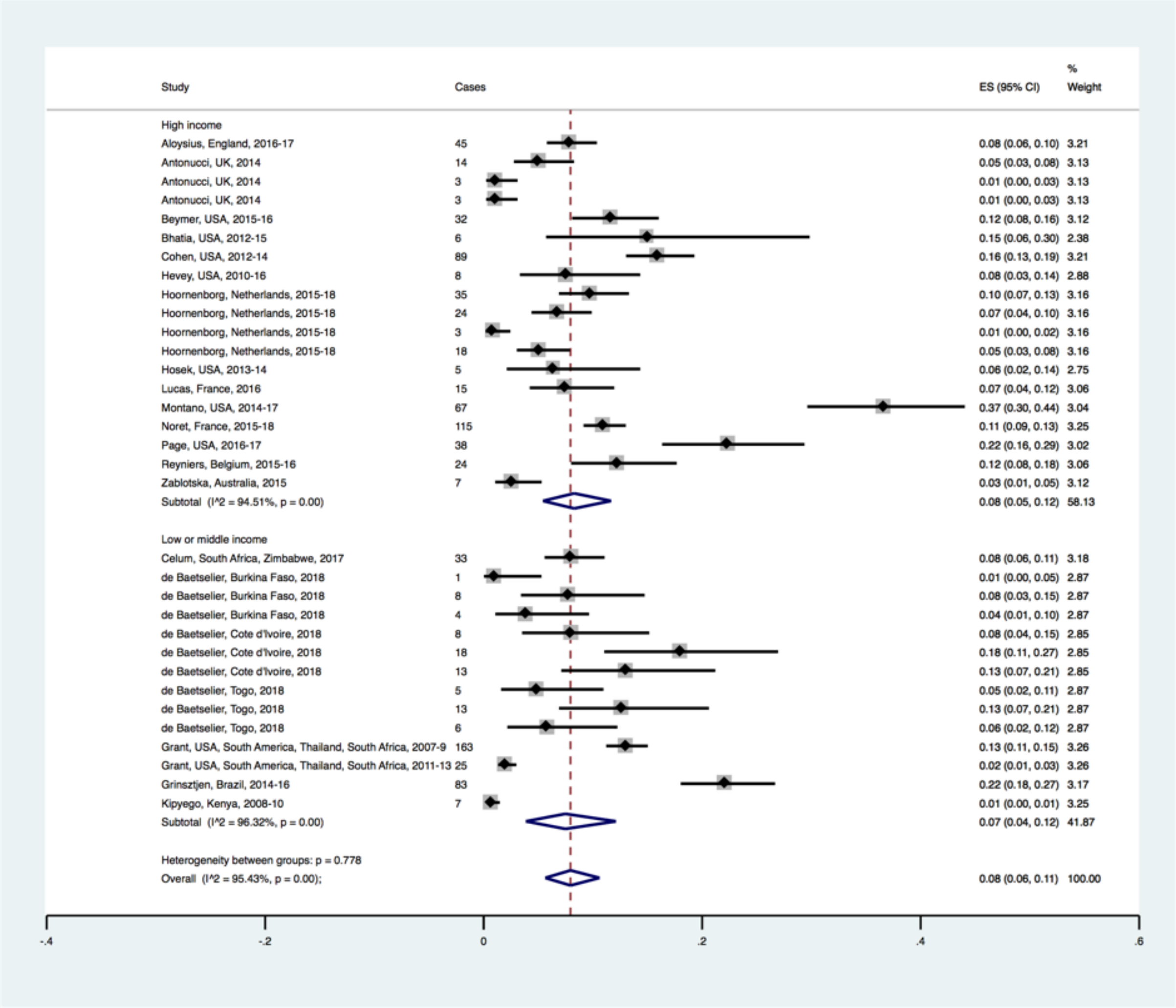

© 2019 Ong JJ et al. *JAMA Network Open.*

eFigure 2, panel E – Random effects meta-analysis of gonorrhoea prevalence by study type

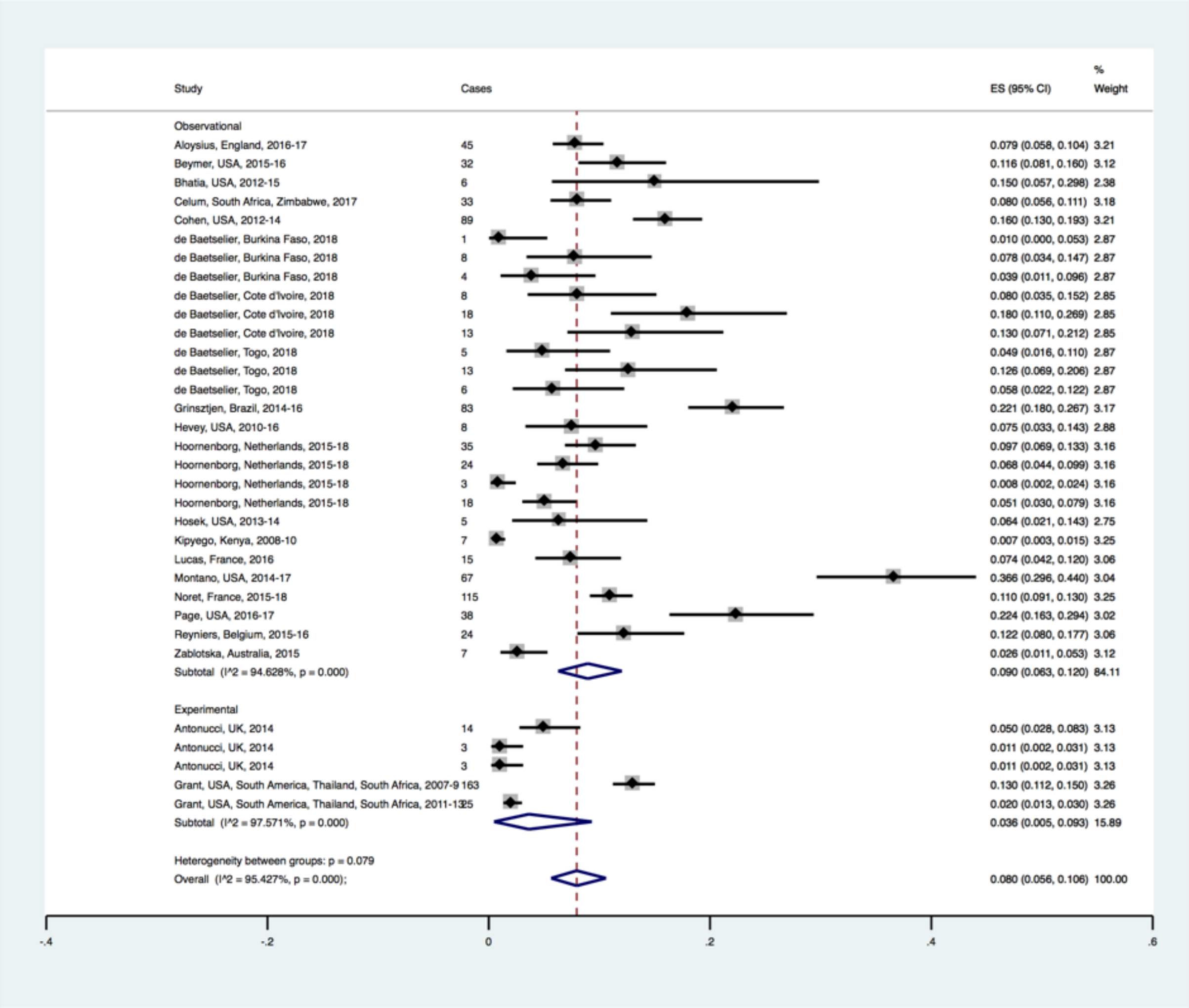

© 2019 Ong JJ et al. *JAMA Network Open.*

eFigure 2, panel F – Random effects meta-analysis of gonorrhoea prevalence by publication status

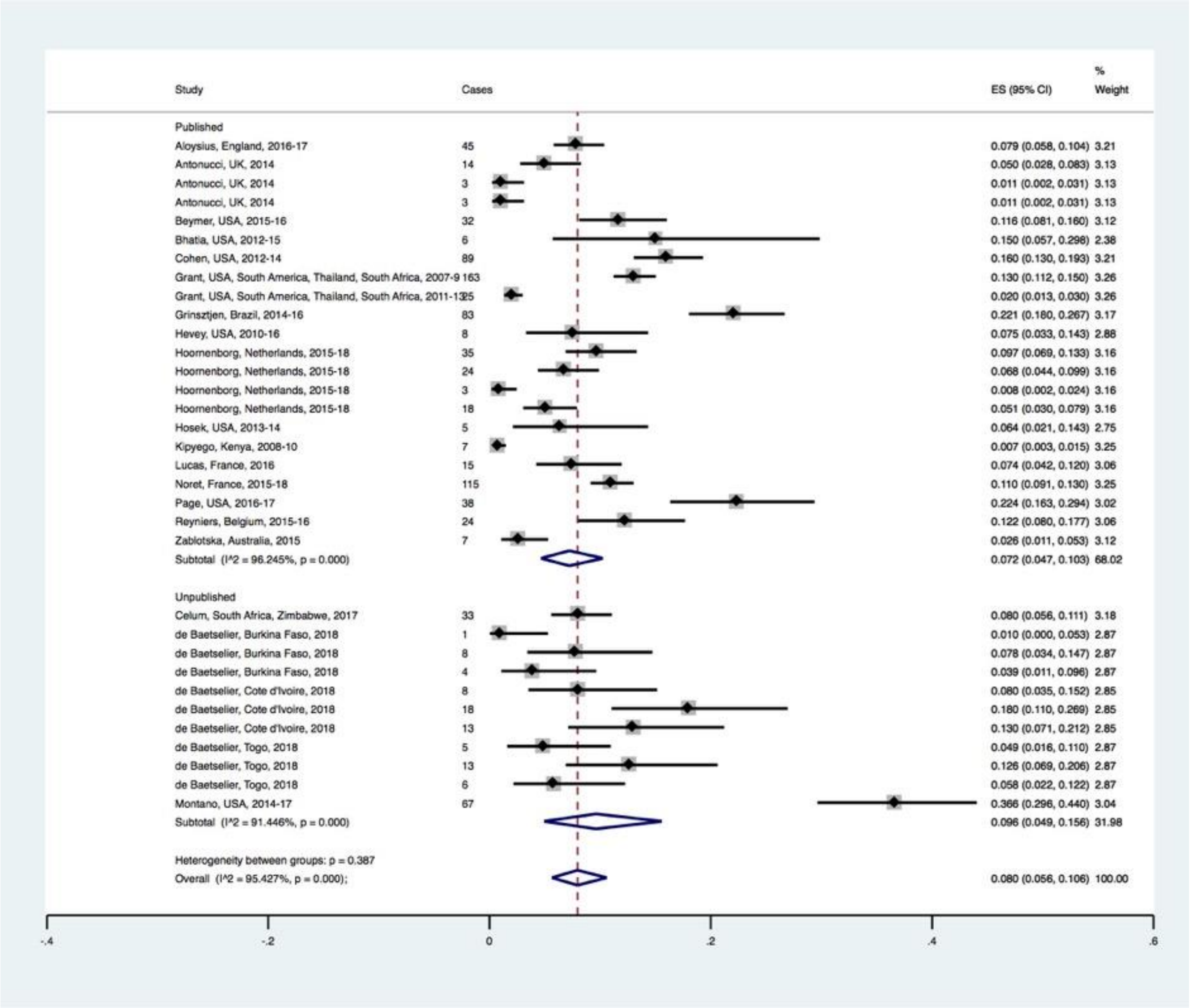

© 2019 Ong JJ et al. *JAMA Network Open.*

eTable 2. Meta-regression Results for the Predictors of Gonorrhea Prevalence and Sources of Between-Study Heterogeneity

Characteristic	Variable	OR (95% CI)	p value	AOR (95% CI)	p value
Anatomical site	Oral	1		1	
	All sites	1.06 (1.00-1.11)	0.05	1.09 (1.00-1.18)	0.05
	Genital	0.92 (0.86-0.99)	0.02	0.97 (0.89-1.07)	0.58
	Anorectal	1.02 (0.95-1.09)	0.63	1.05 (0.96-1.15)	0.28
Population	Mixed	1		1	
	MSM only	1.02 (0.94-1.10)	0.66	1.09 (1.01-1.18)	0.04
Country income level	LMIC	1		1	
	High	1.01 (0.95-1.07)	0.74	0.97 (0.92-1.03)	0.31
Study type	Observational	1		1	
	Experimental	0.96 (0.89-1.02)	0.18	0.97 (0.91-1.03)	0.36

AOR = adjusted odds ratio; LMIC = low-middle income country; MSM = men who have sex with men; OR = odds ratio; RCT = randomized controlled trial

© 2019 Ong JJ et al. *JAMA Network Open.*

eFigure 2, panel F - Funnel plot and Egger's test for gonorrhoea prevalence

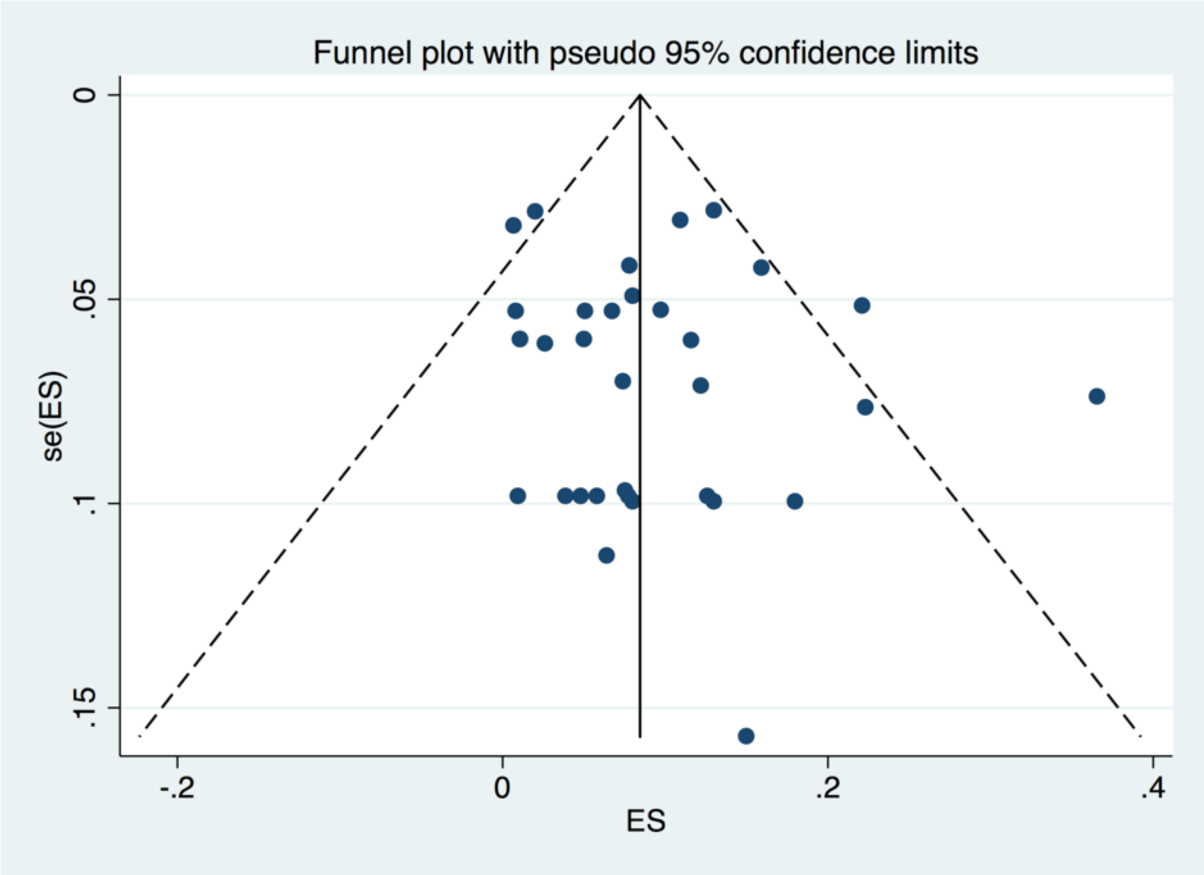

Egger's test = 0.22

© 2019 Ong JJ et al. *JAMA Network Open.*

eFigure 3. Random Effects Meta-Analysis of Early Syphilis Prevalence
panel A - Random effects meta-analysis of early syphilis prevalence

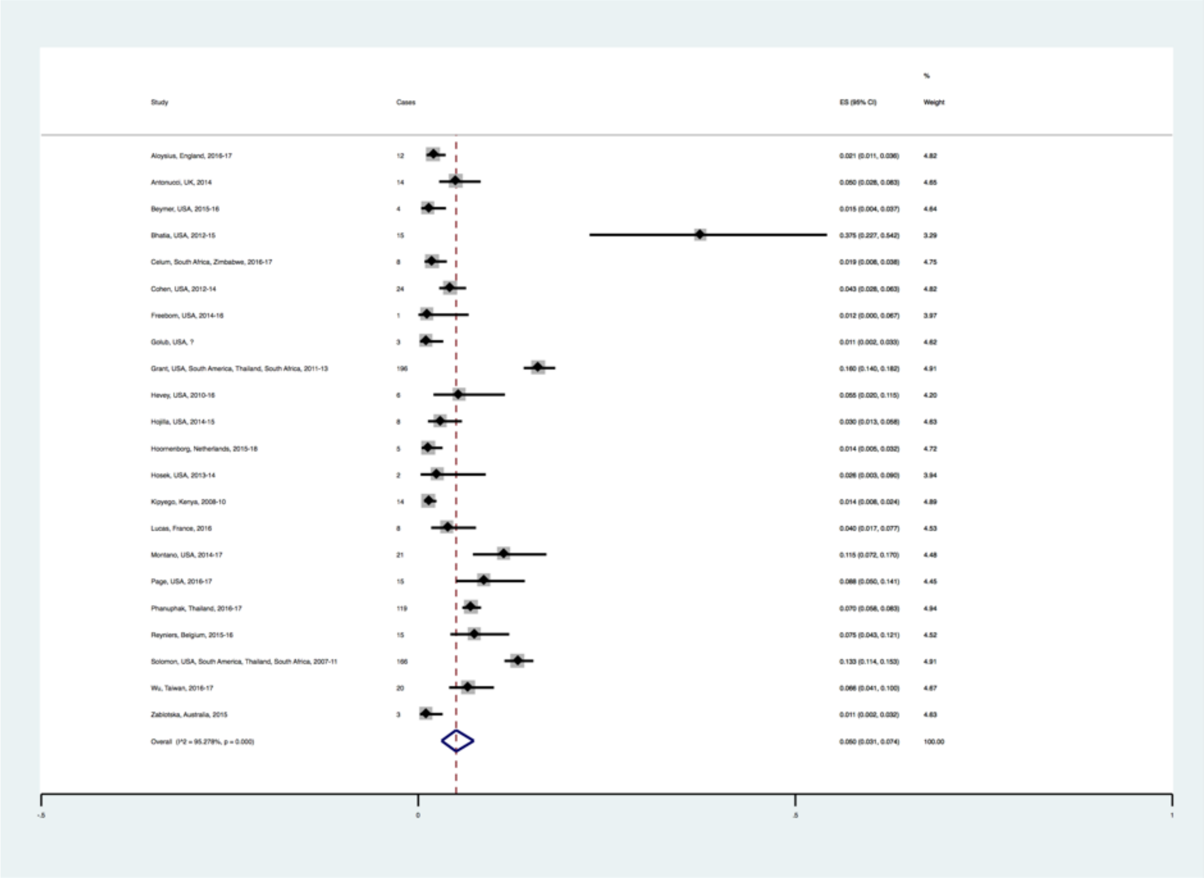

© 2019 Ong JJ et al. *JAMA Network Open.*

eFigure 3, panel B - Random effects meta-analysis of early syphilis prevalence by MSM status of the study population

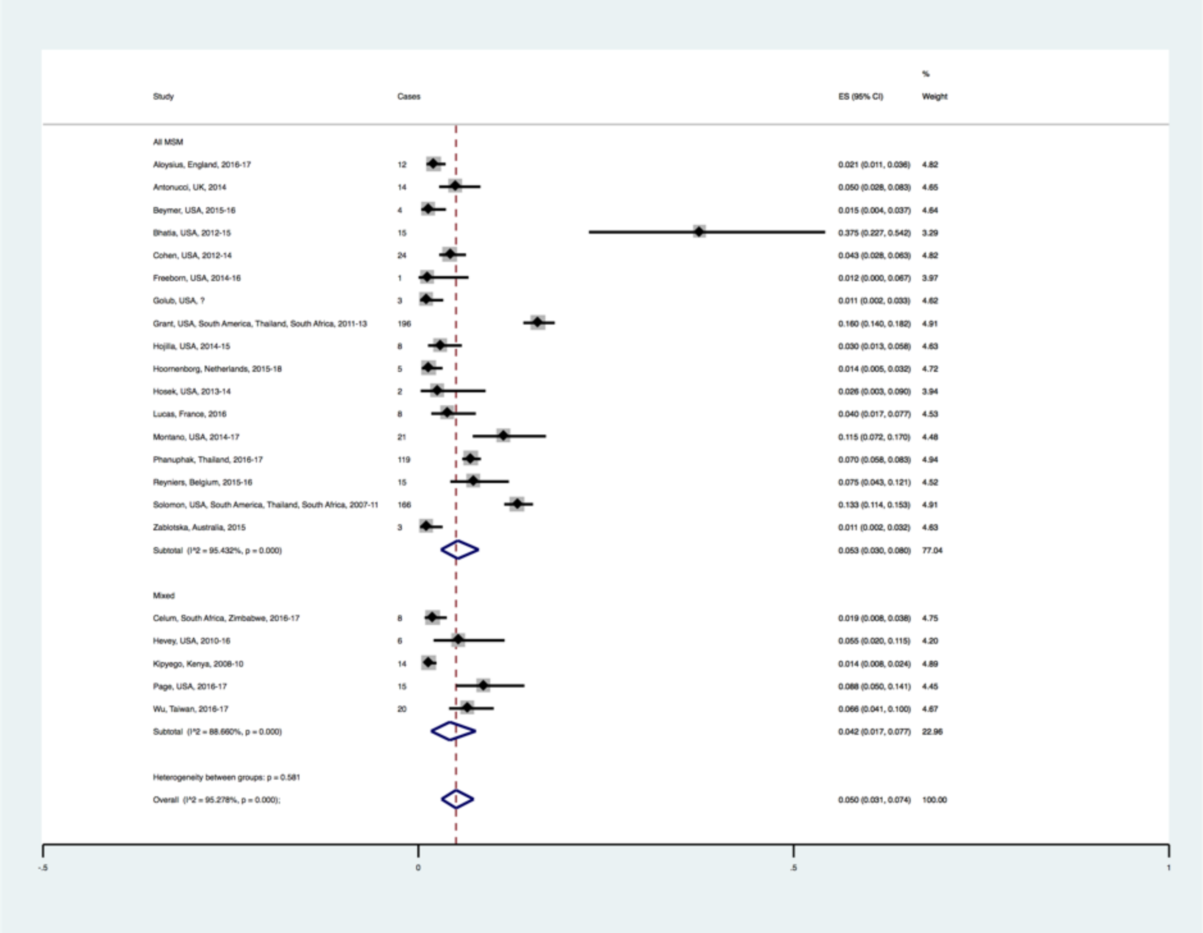

© 2019 Ong JJ et al. *JAMA Network Open.*

eFigure 3, panel C - Random effects meta-analysis of early syphilis prevalence by country income level

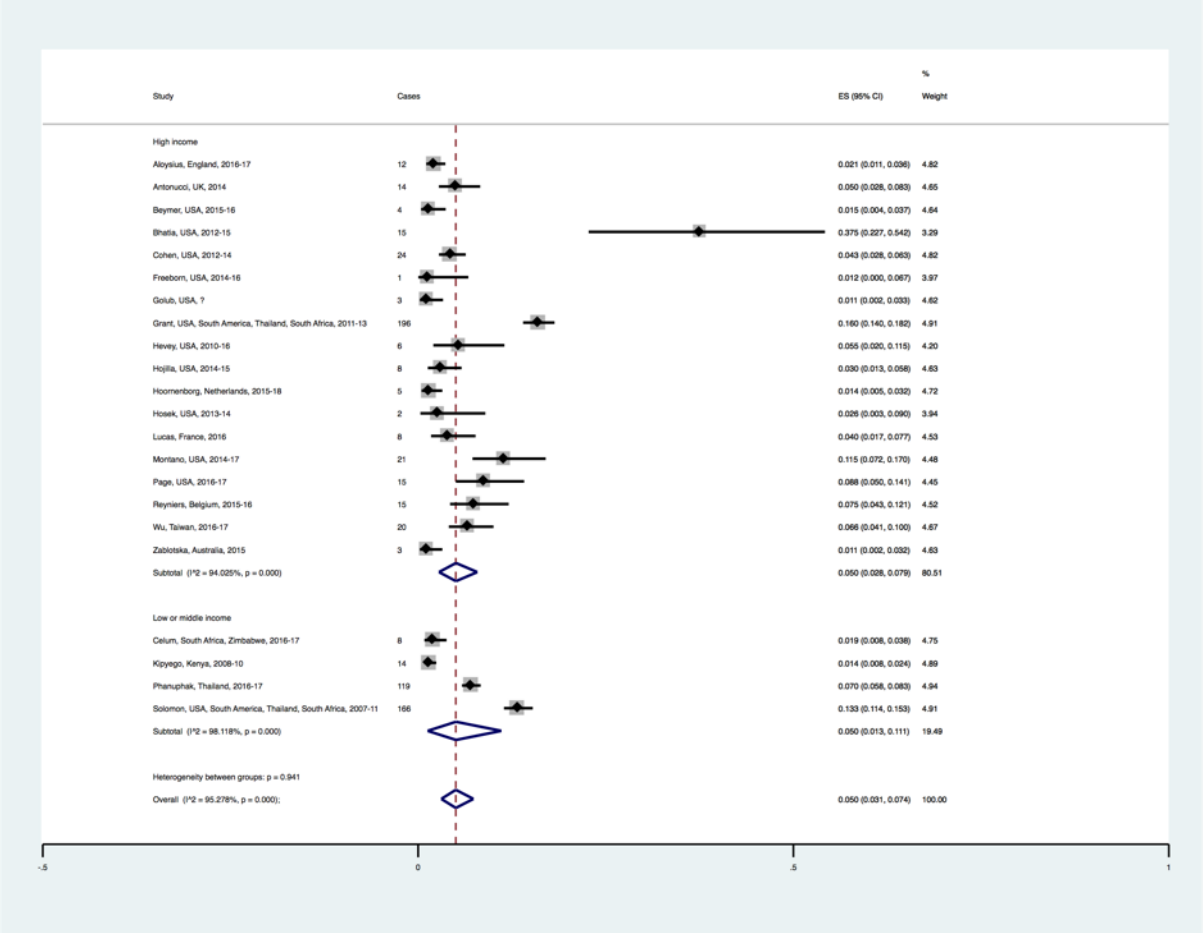

© 2019 Ong JJ et al. *JAMA Network Open.*

eFigure 3, panel D - Random effects meta-analysis of early syphilis prevalence by study type

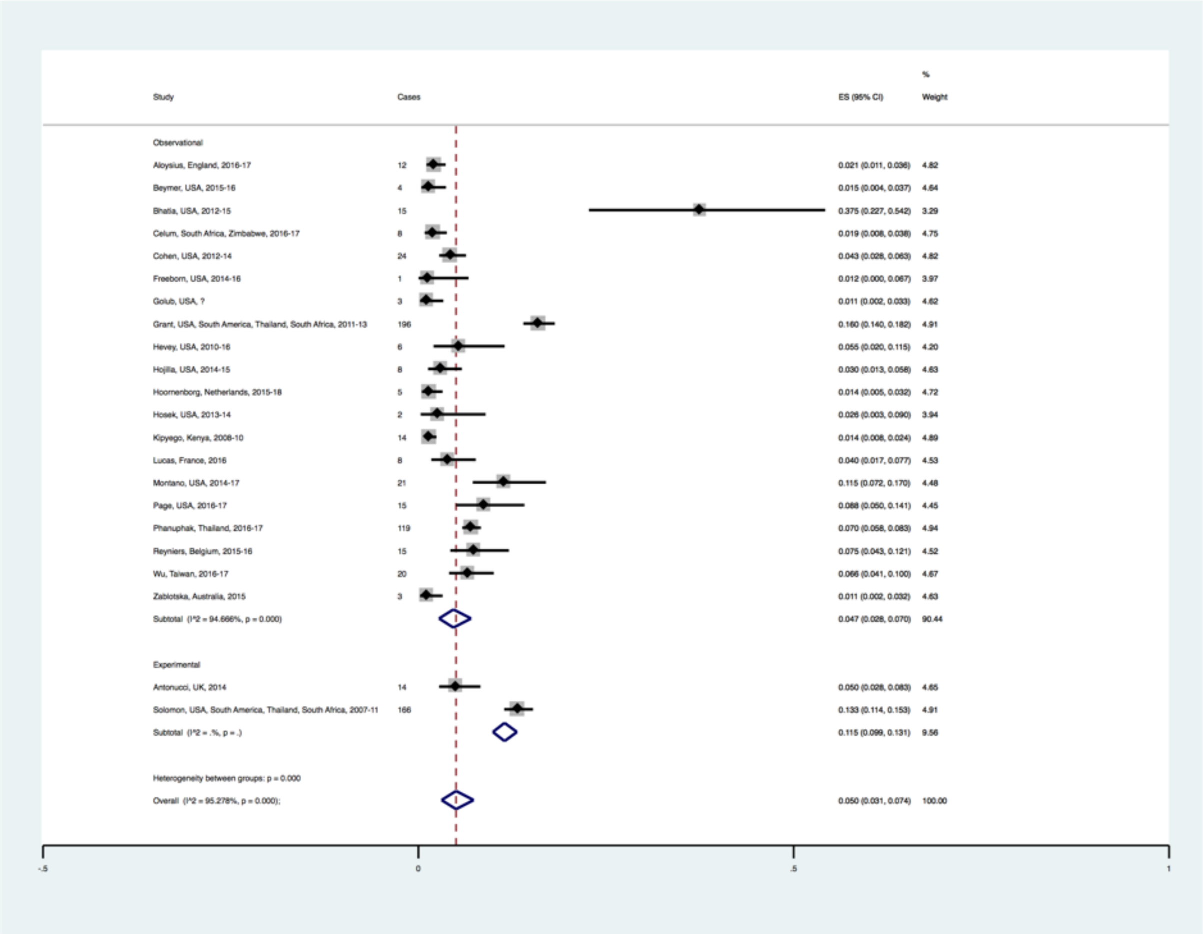

© 2019 Ong JJ et al. *JAMA Network Open.*

eFigure 3, panel E - Random effects meta-analysis of early syphilis prevalence by publication status

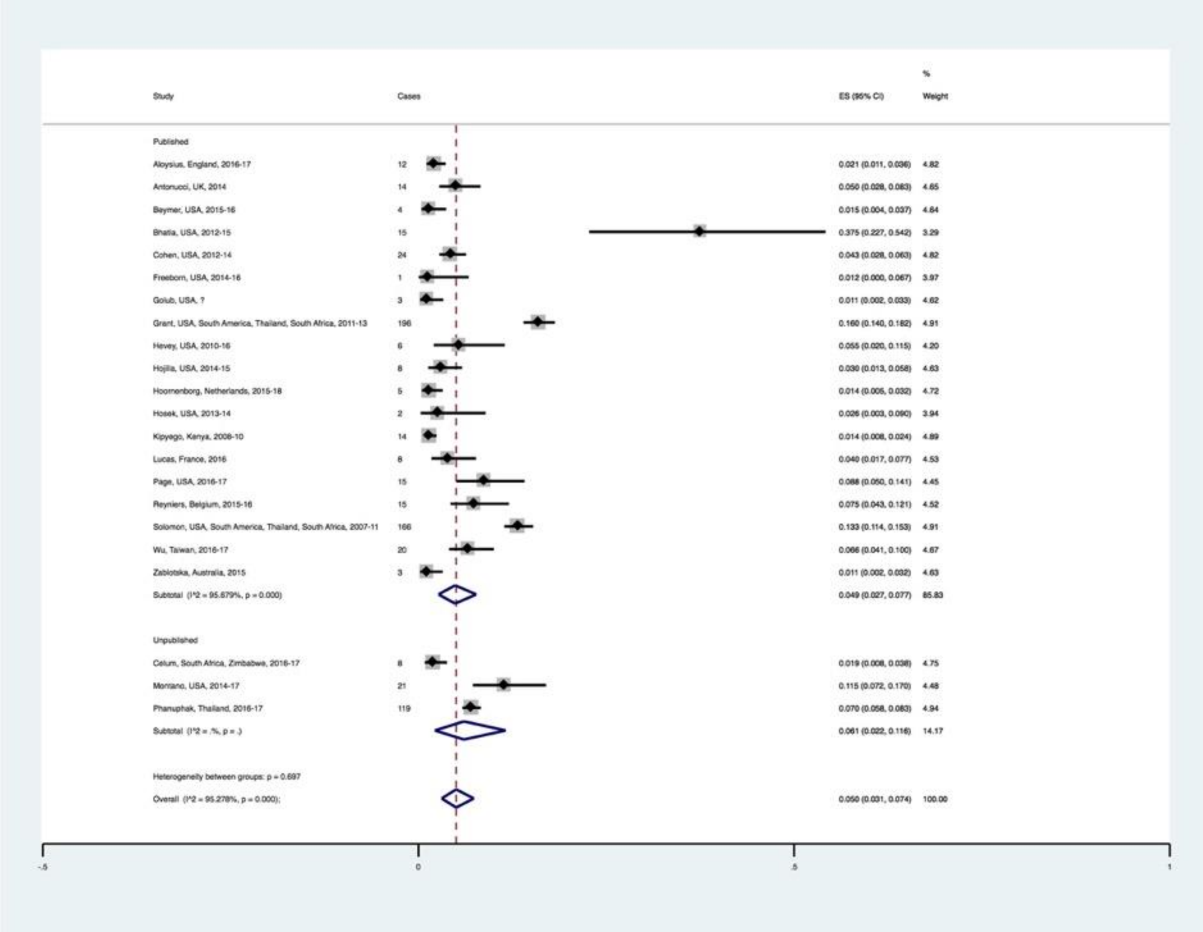

© 2019 Ong JJ et al. *JAMA Network Open.*

eTable 3. Meta-regression Results for the Predictors of Early Syphilis Prevalence and Sources of Between-Study Heterogeneity

Characteristic	Variable	OR (95% CI)	p value	AOR (95% CI)	p value
Population	Mixed	1		1	
	MSM only	1.03 (0.97-1.11)	0.33	1.02 (0.95-1.11)	0.51
Country income level	LMIC	1		1	
	High	0.99 (0.93-1.06)	0.85	1.00 (0.93-1.07)	0.95
Study type	Observational	1		1	
	Experimental	1.06 (0.97-1.15)	0.19	1.05 (0.95-1.15)	0.32

AOR = adjusted odds ratio; LMIC = low-middle income country; MSM = men who have sex with men; OR = odds ratio; RCT = randomized controlled trial

© 2019 Ong JJ et al. *JAMA Network Open.*

eFigure 3, panel F – Funnel plot and Egger's test of early syphilis prevalence

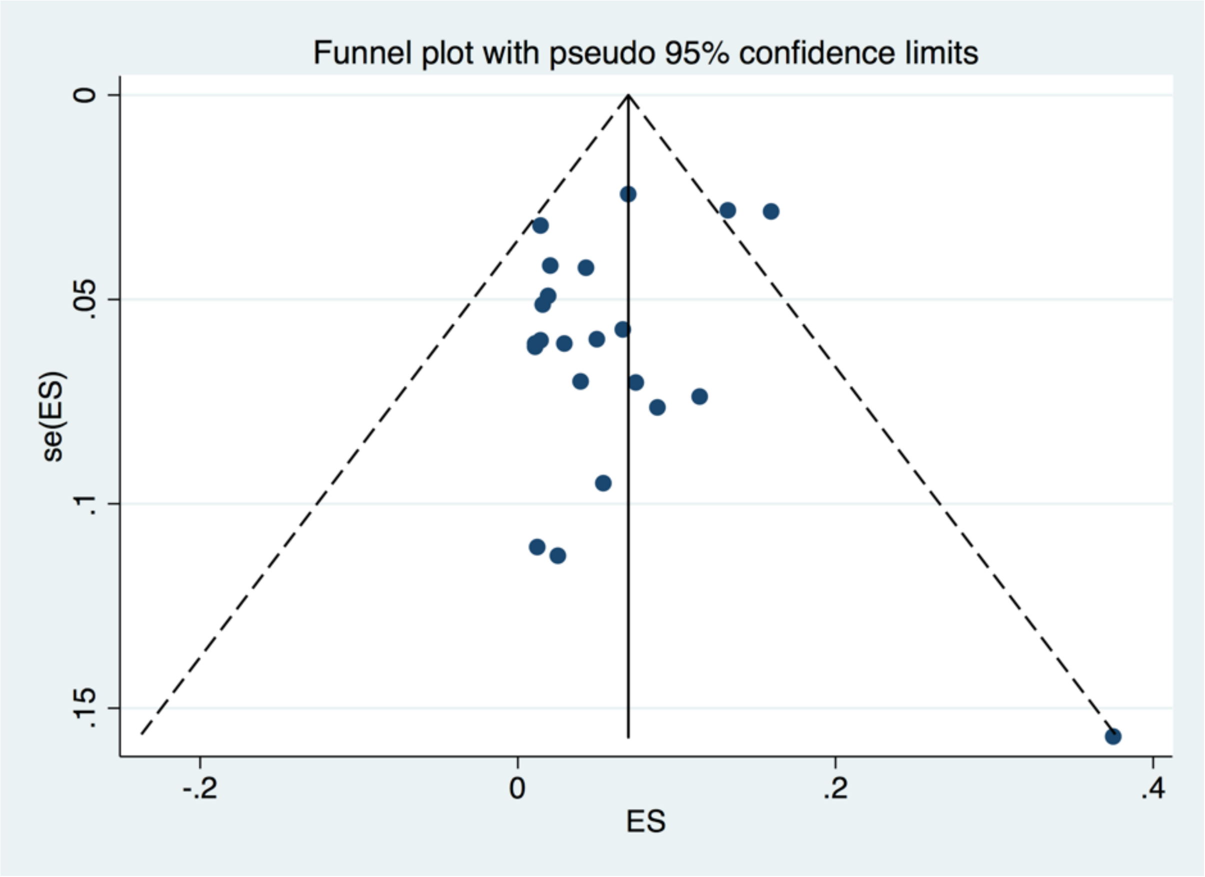

Egger's test = 0.22

© 2019 Ong JJ et al. *JAMA Network Open.*

eFigure 4. Random Effects Meta-Analysis of Any Chlamydia, Gonorrhea, or Early Syphilis Prevalence

panel A - Random effects meta-analysis of any chlamydia, gonorrhoea or early syphilis prevalence

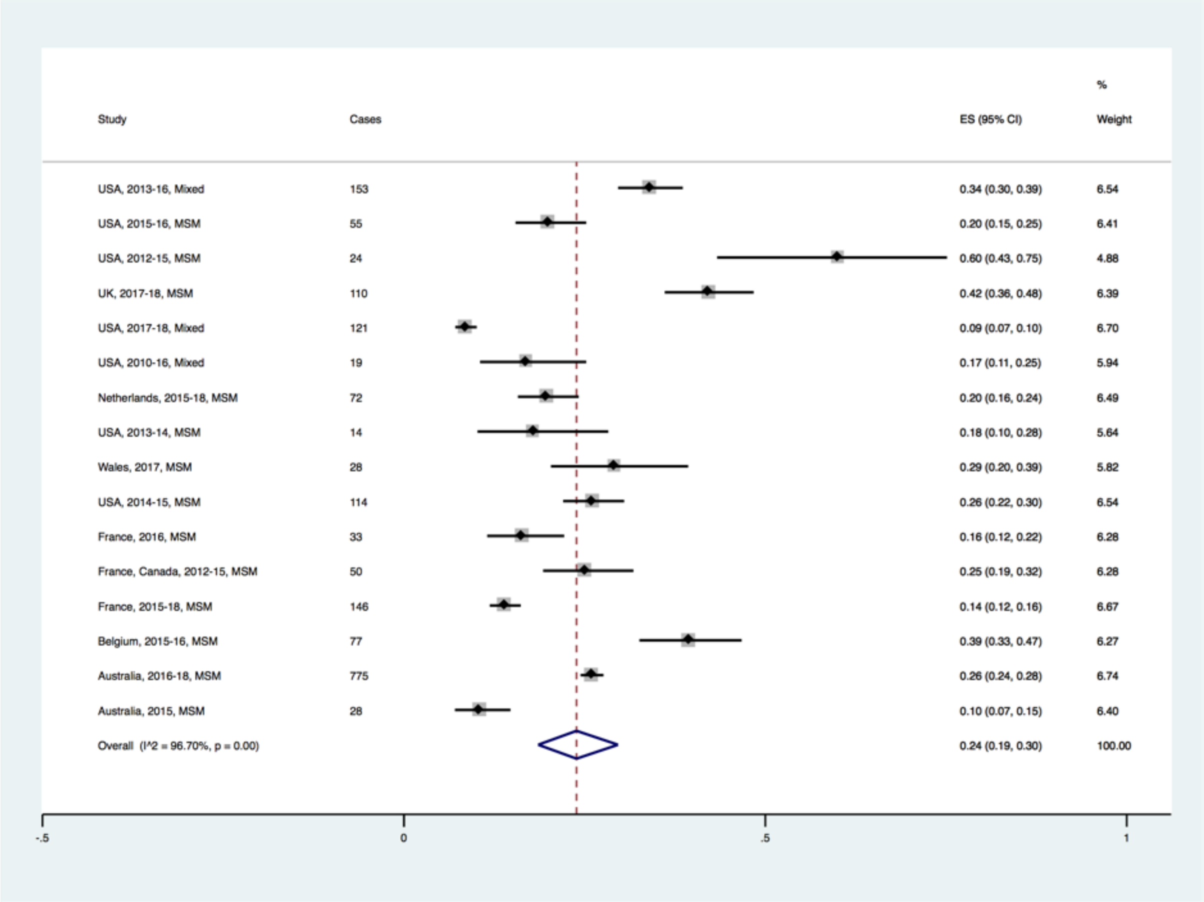

© 2019 Ong JJ et al. *JAMA Network Open.*

eFigure 4, panel B - Random effects meta-analysis of any chlamydia, gonorrhoea or early syphilis prevalence by MSM status of the study population

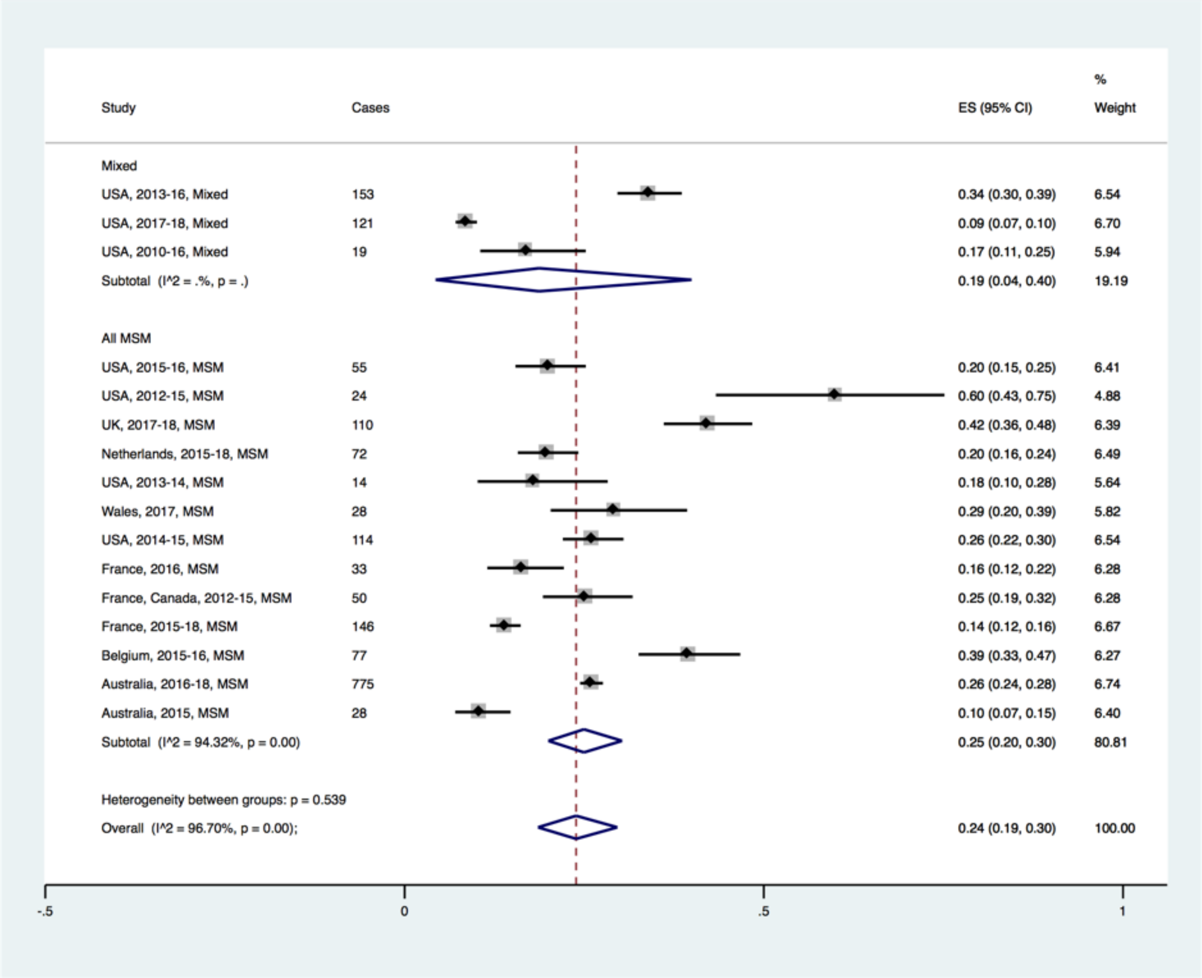

© 2019 Ong JJ et al. *JAMA Network Open.*

eFigure 4, panel C - Random effects meta-analysis of any chlamydia, gonorrhoea or early syphilis prevalence by study type

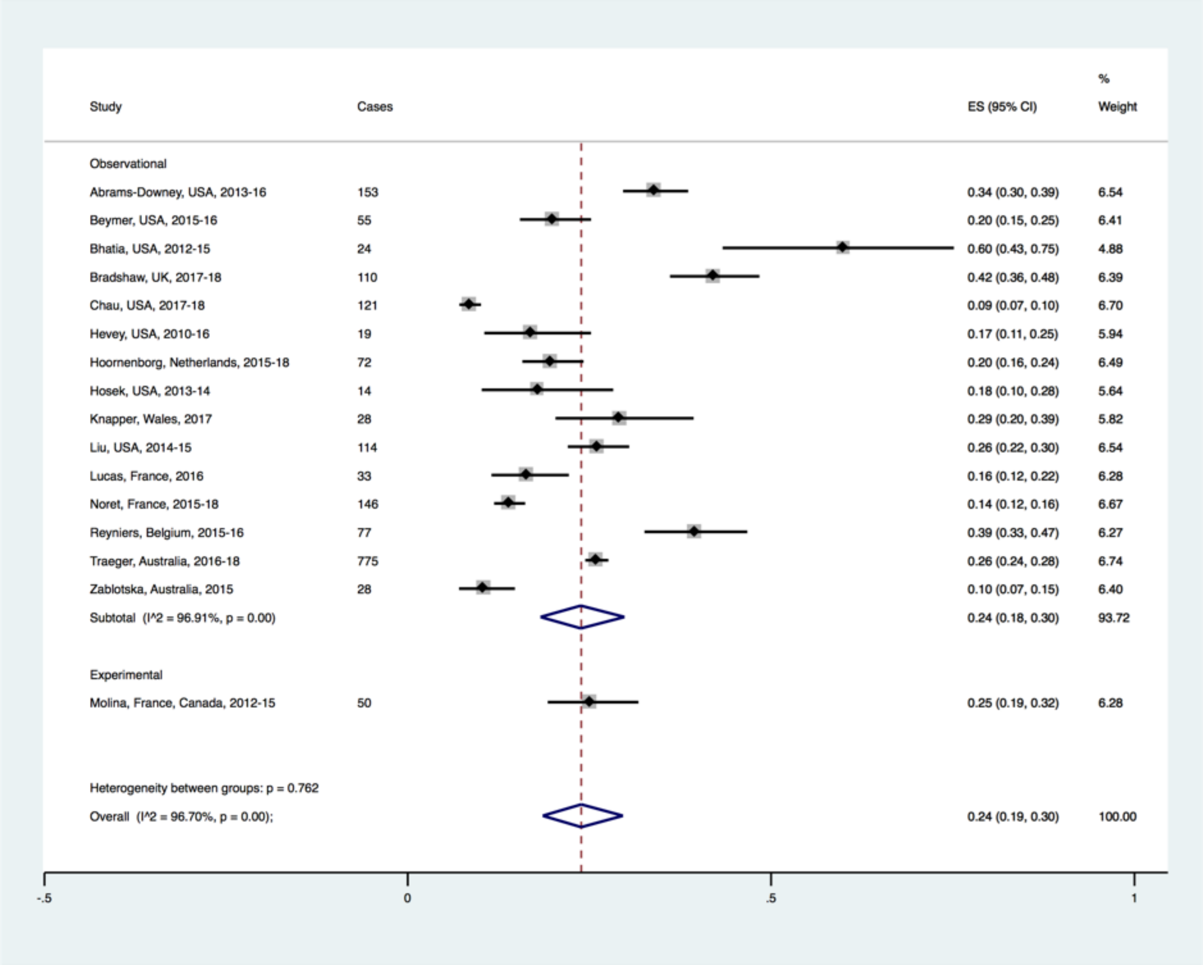

© 2019 Ong JJ et al. *JAMA Network Open.*

eFigure 4, panel D - Random effects meta-analysis of any chlamydia, gonorrhoea or early syphilis prevalence by publication status

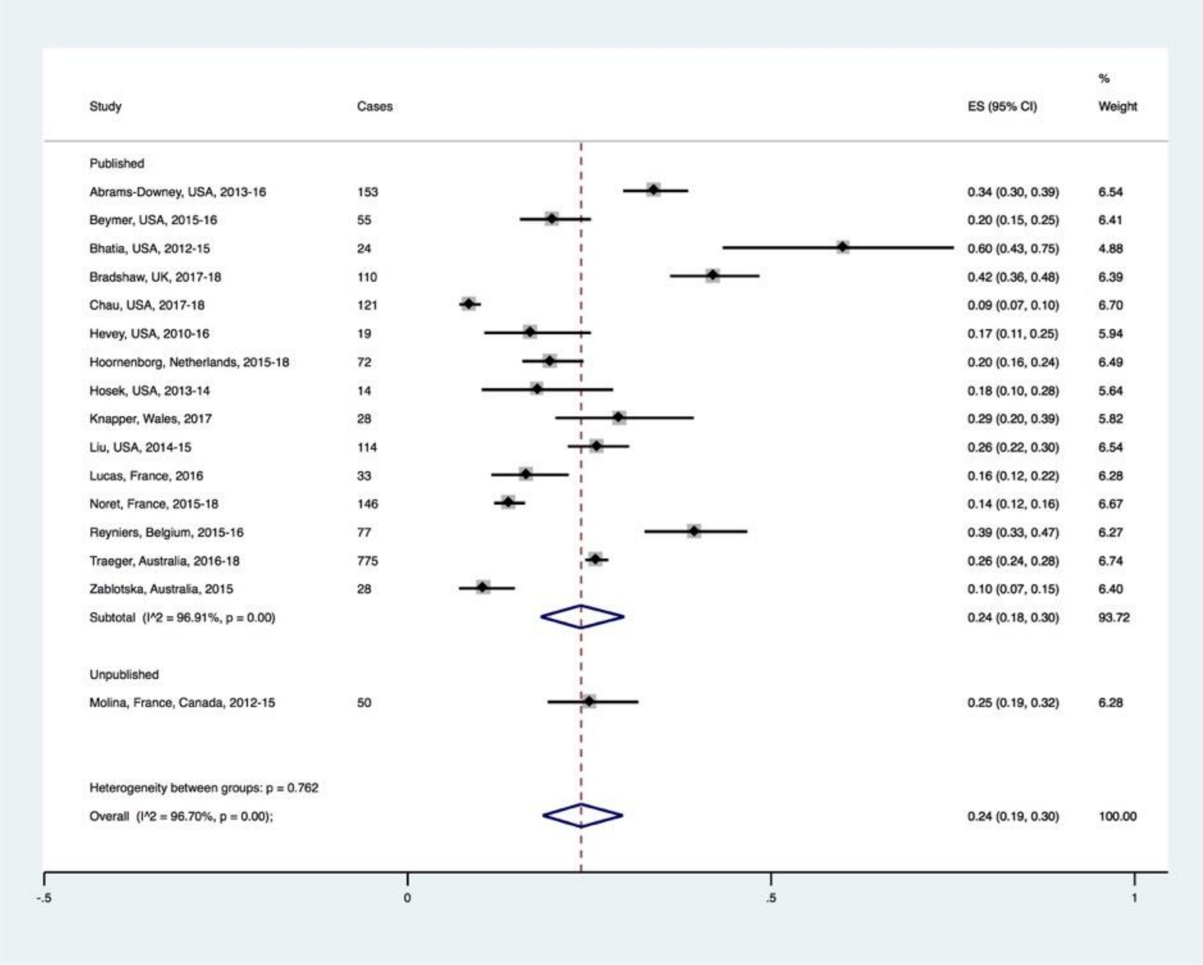

© 2019 Ong JJ et al. *JAMA Network Open.*

eTable 4. Meta-regression Results for the Predictors of Any Chlamydia, Gonorrhea, or Early Syphilis Prevalence and Sources of Between-Study Heterogeneity

Characteristic	Variable	OR (95% CI)	p value	AOR (95% CI)	p value
Population	Mixed	1		1	
	MSM only	1.05 (0.90-1.23)	0.49	1.05 (0.89-1.24)	0.51
Country income level*	LMIC	-	-	-	-
	High	-	-	-	-
Study type	Observational	1		1	
	Experimental	1.02 (0.77-1.33)	0.91	1.00 (0.76-1.33)	0.98

*All studies are from high income level

AOR = adjusted odds ratio; LMIC = low-middle income country; MSM = men who have sex with men; OR = odds ratio; RCT = randomized controlled trial

© 2019 Ong JJ et al. *JAMA Network Open.*

eFigure 4, panel E - Funnel plot and Egger's test any chlamydia, gonorrhoea or early syphilis prevalence

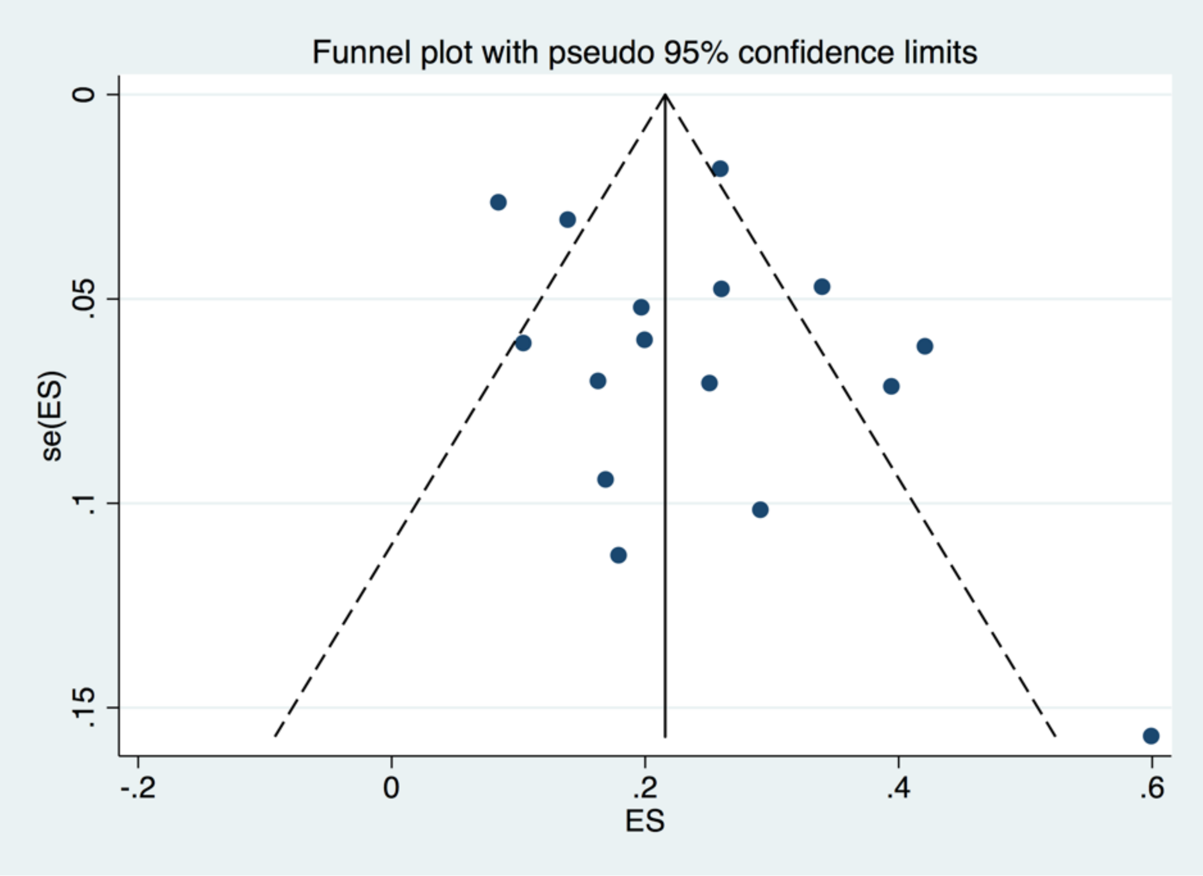

Egger's test = 0.06

© 2019 Ong JJ et al. *JAMA Network Open.*

eFigure 5. Random Effects Meta-Analysis of Hepatitis B Prevalence
panel A - Random effects meta-analysis of Hepatitis B prevalence

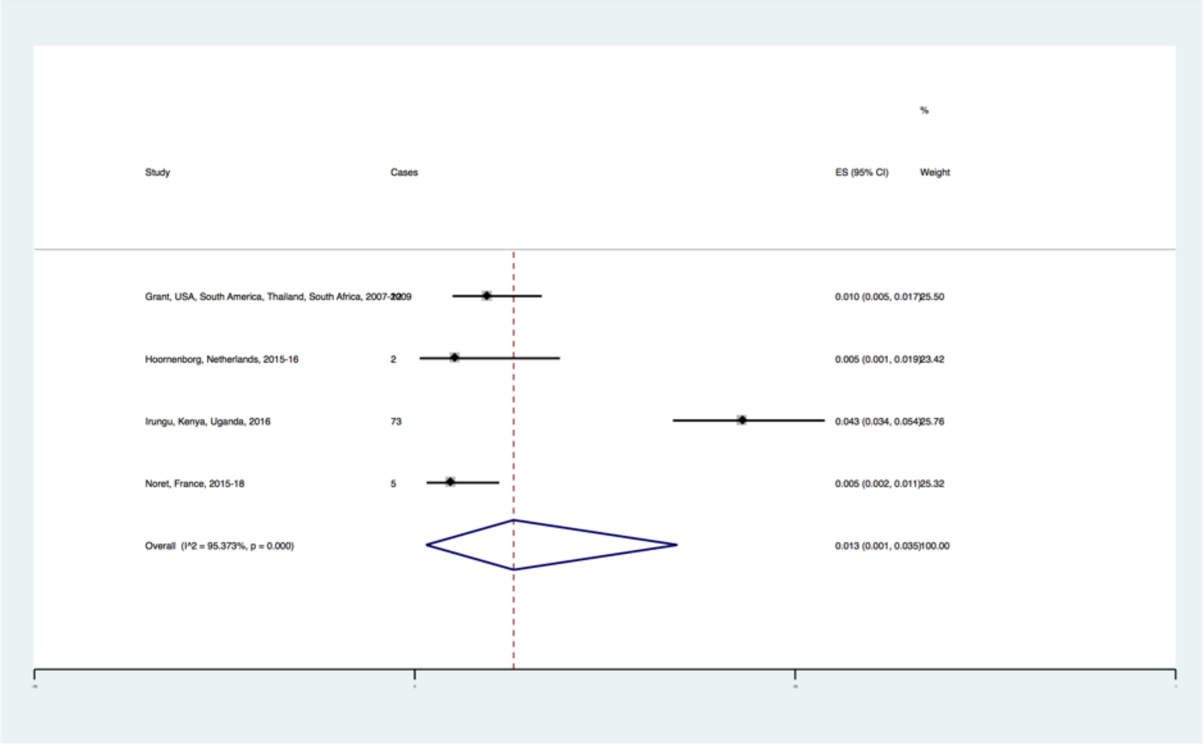

© 2019 Ong JJ et al. *JAMA Network Open.*

eFigure 5, panel B - Random effects meta-analysis of Hepatitis B prevalence by MSM status of the study population

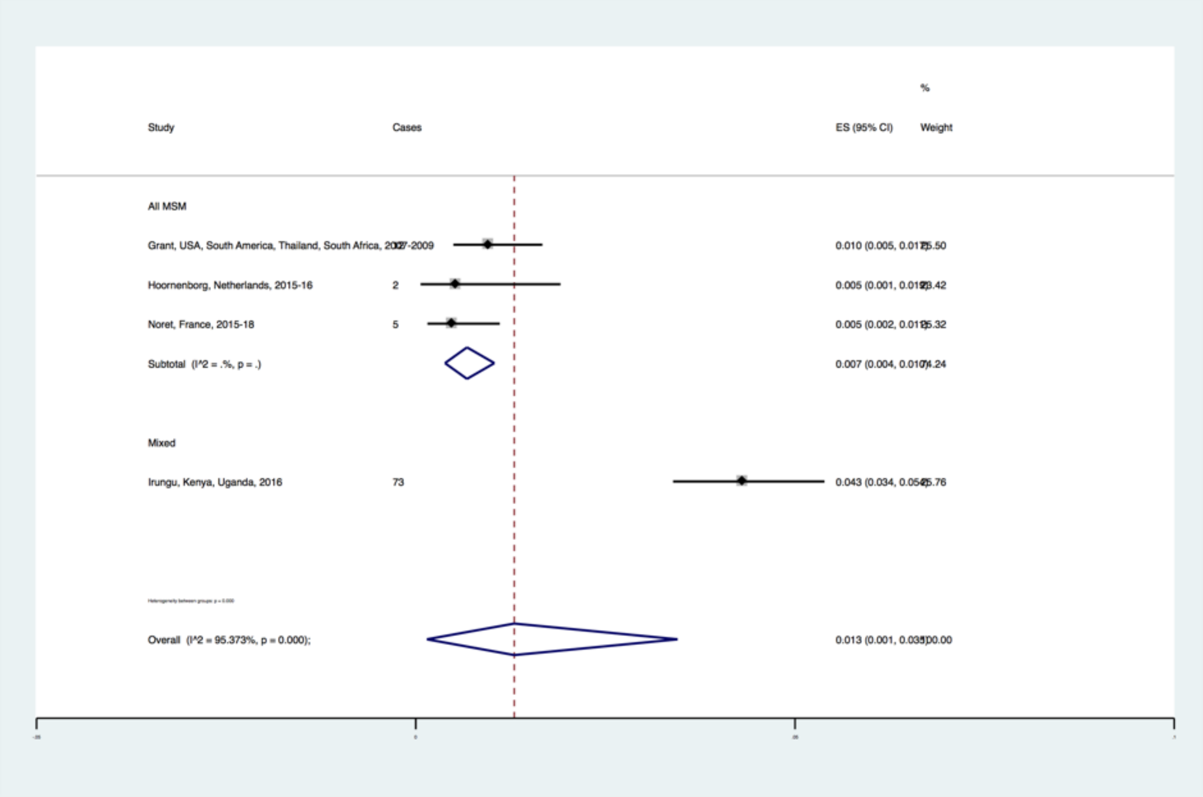

© 2019 Ong JJ et al. *JAMA Network Open.*

eFigure 5, panel C - Random effects meta-analysis of Hepatitis B prevalence by country income level

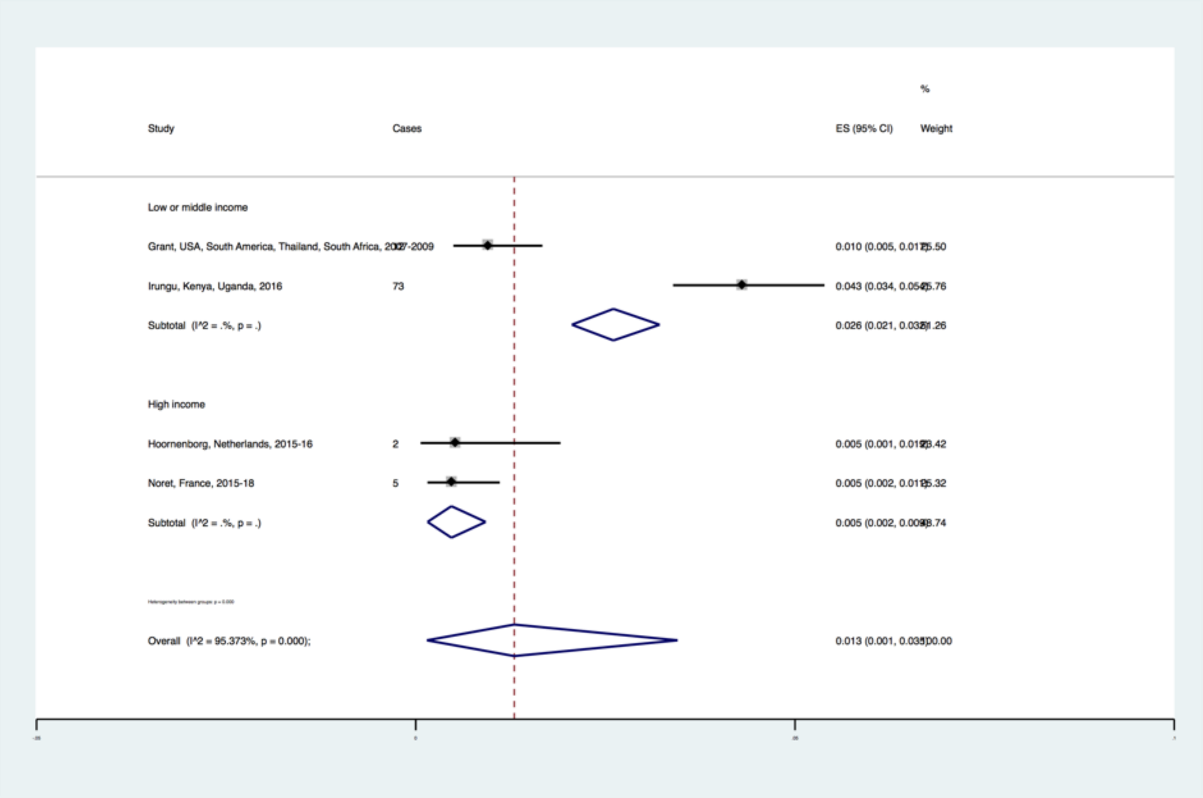

© 2019 Ong JJ et al. *JAMA Network Open.*

eFigure 5, panel D - Random effects meta-analysis of Hepatitis B prevalence by study type

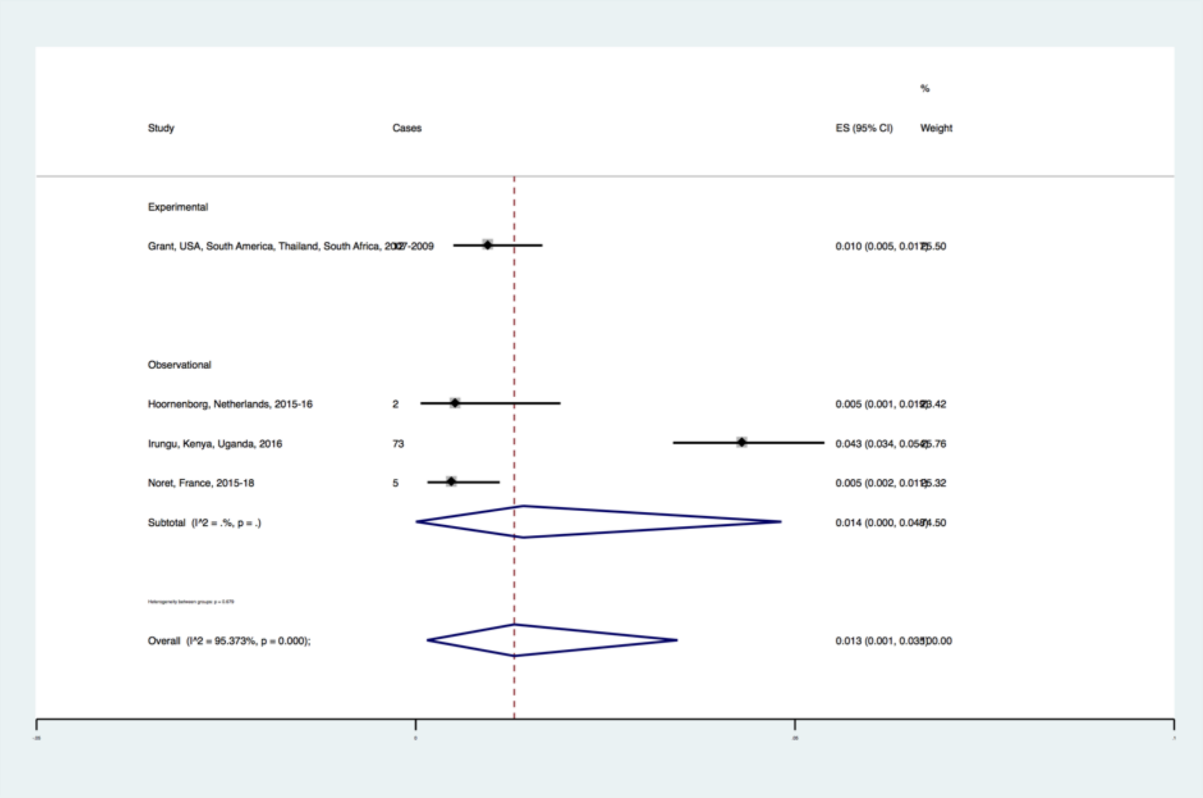

© 2019 Ong JJ et al. *JAMA Network Open.*

eFigure 5, panel E - Funnel plot and Egger’s test for Hepatitis B prevalence

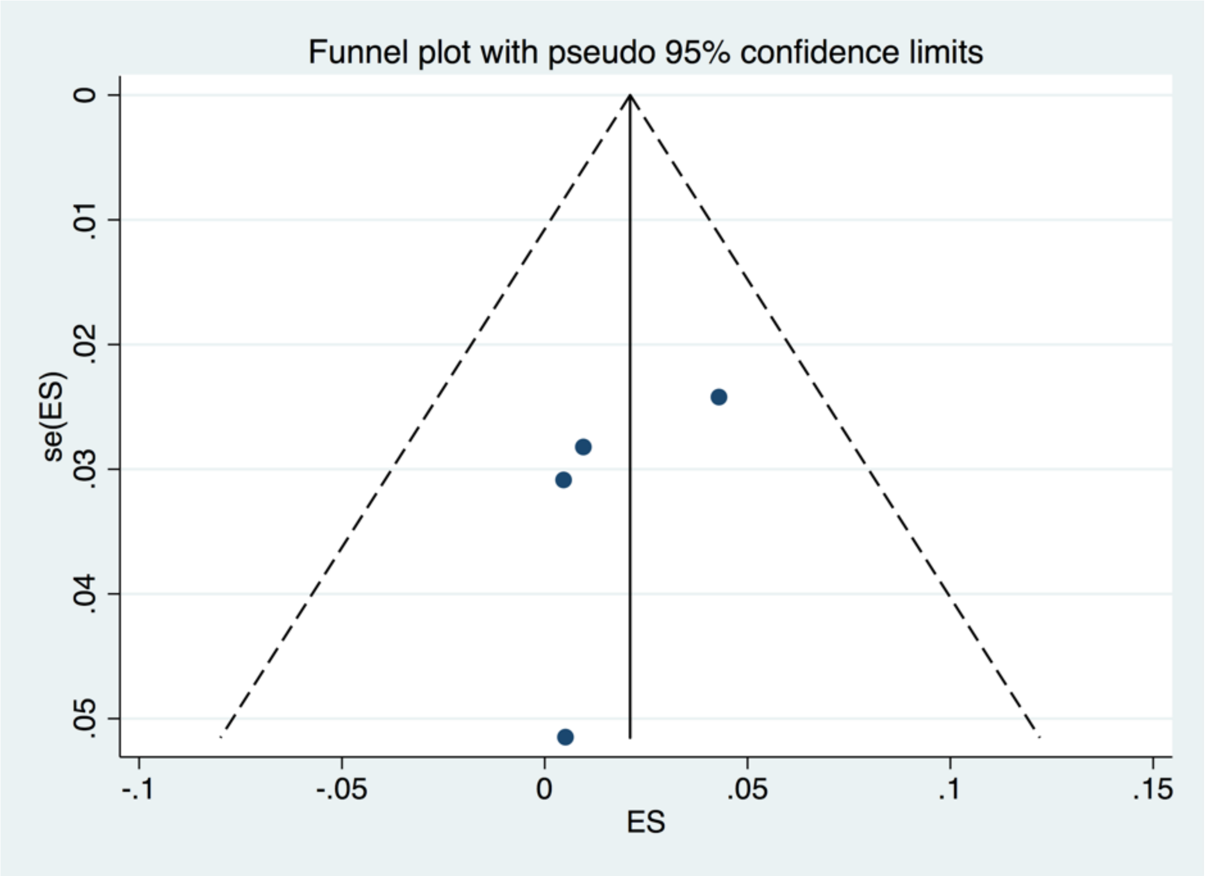

Egger’s test = 0.33

© 2019 Ong JJ et al. *JAMA Network Open.*

eFigure 6. Random Effects Meta-Analysis of Hepatitis C Prevalence
panel A - Random effects meta-analysis of Hepatitis C prevalence

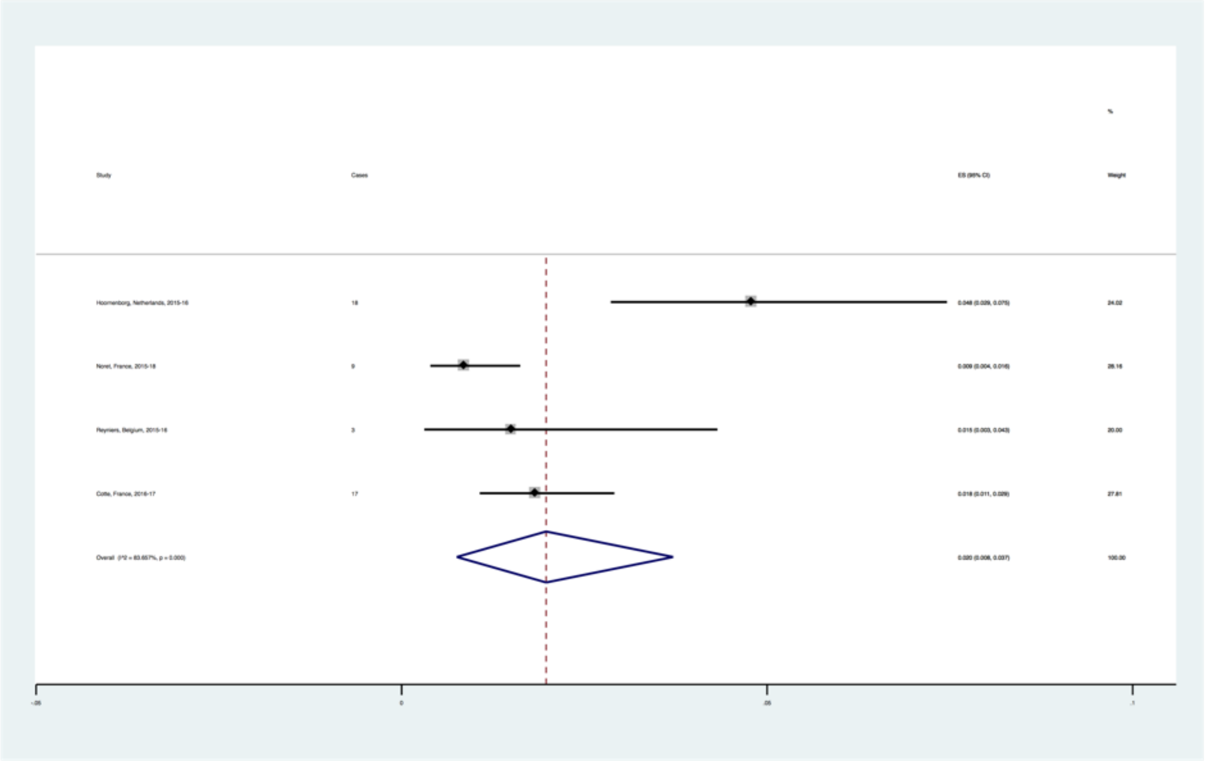

© 2019 Ong JJ et al. *JAMA Network Open.*

eFigure 6, panel B - Random effects meta-analysis of Hepatitis C prevalence by MSM status

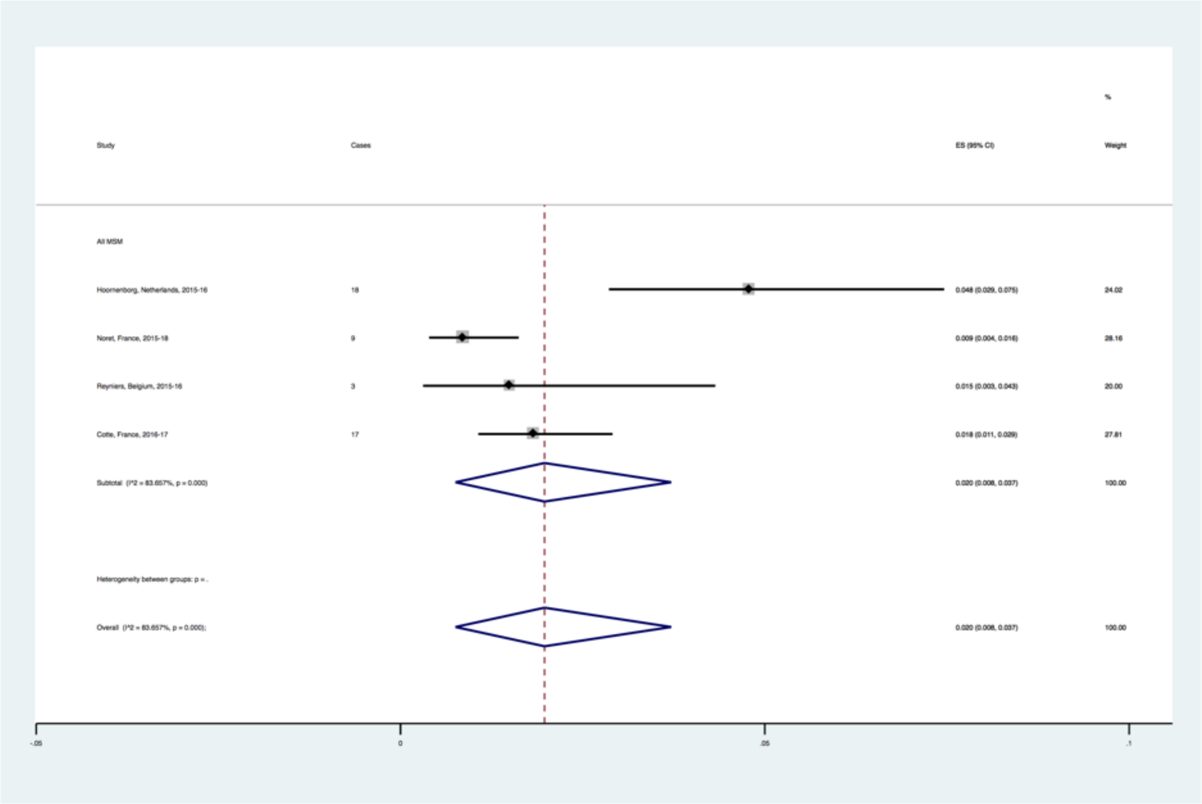

© 2019 Ong JJ et al. *JAMA Network Open.*

eFigure 6, panel C - Funnel plot and Egger's test for Hepatitis C prevalence

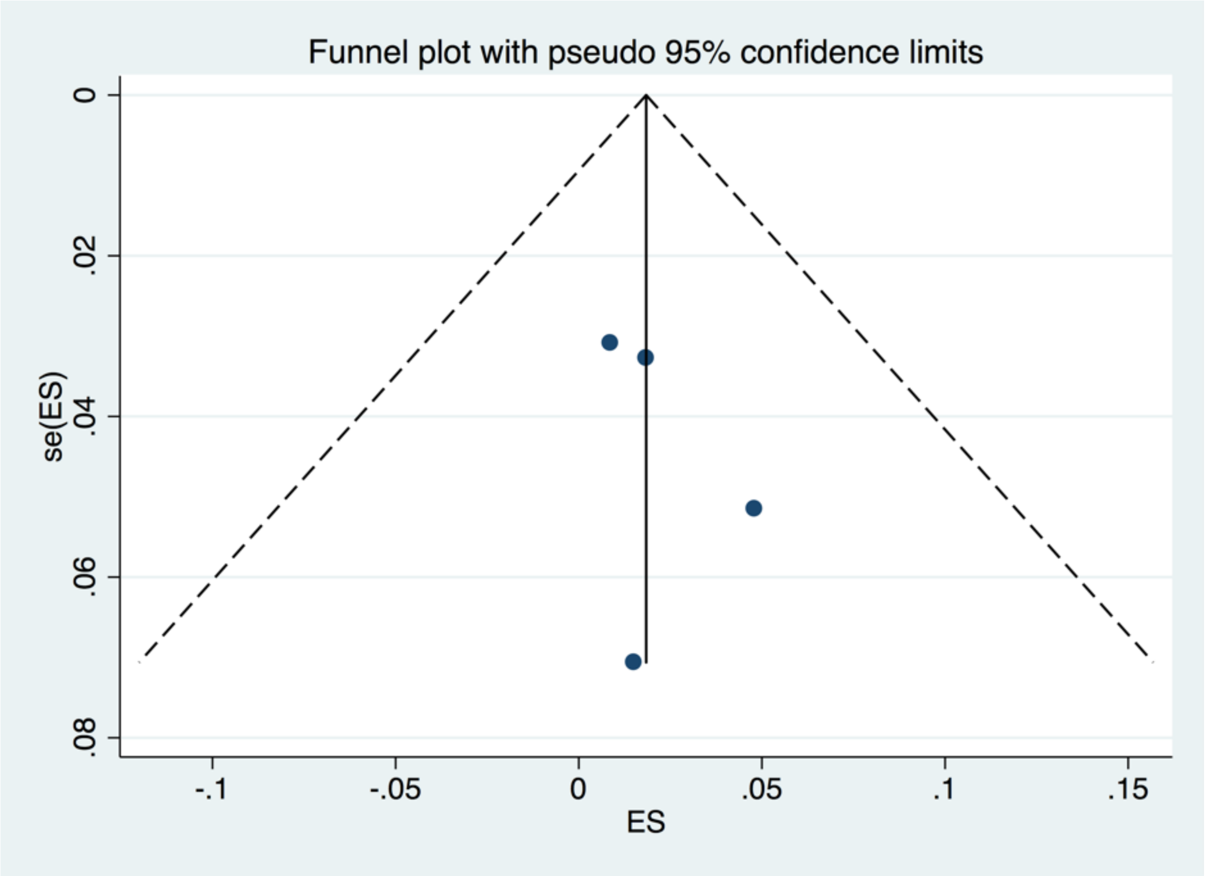

Egger's test = 0.72

© 2019 Ong JJ et al. *JAMA Network Open.*

eFigure 7. Random Effects Meta-Analysis of Chlamydia Incidence
panel A - Random effects meta-analysis of chlamydia incidence

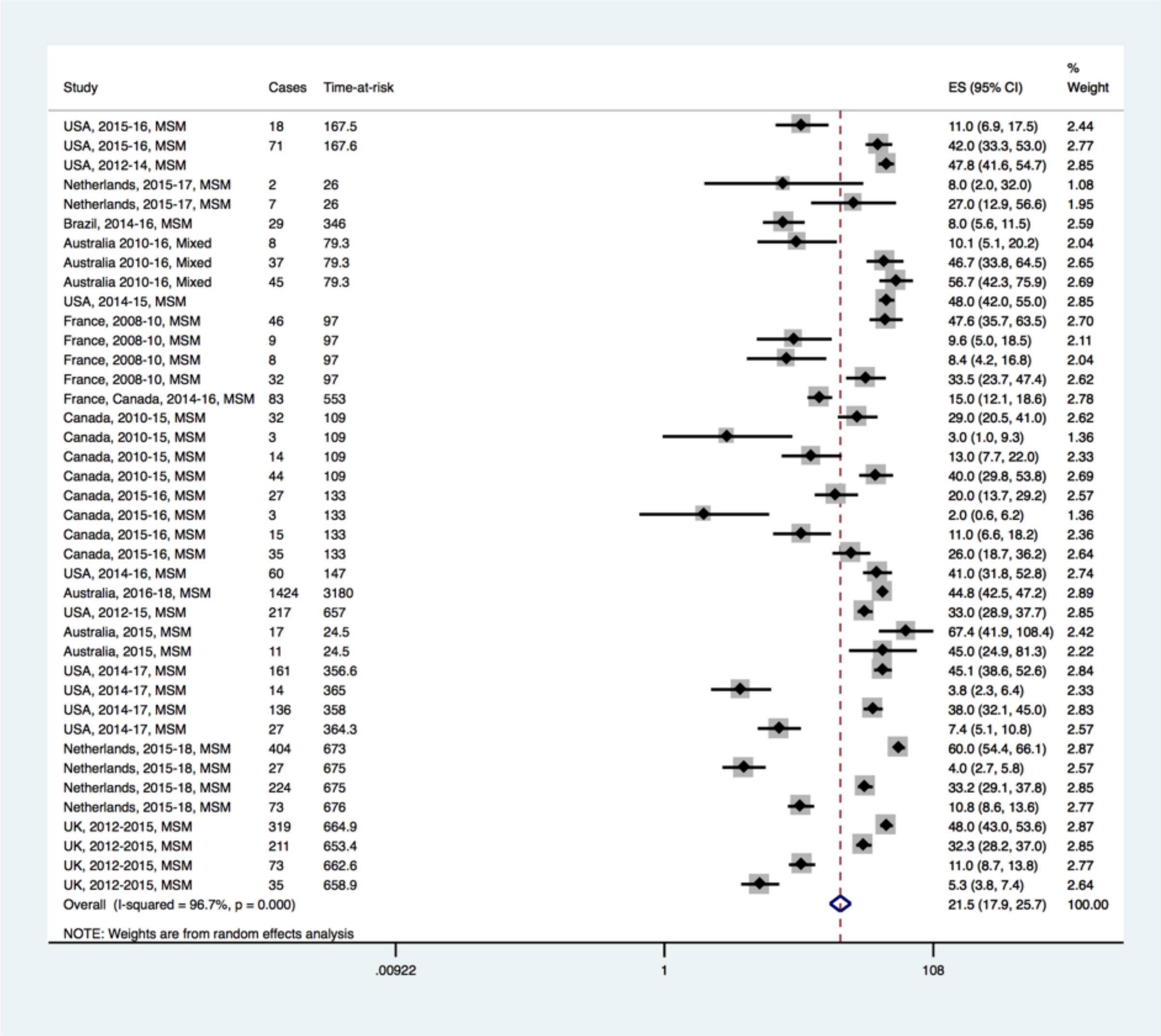

© 2019 Ong JJ et al. *JAMA Network Open.*

eFigure 7, panel B - Random effects meta-analysis of chlamydia incidence by anatomical site

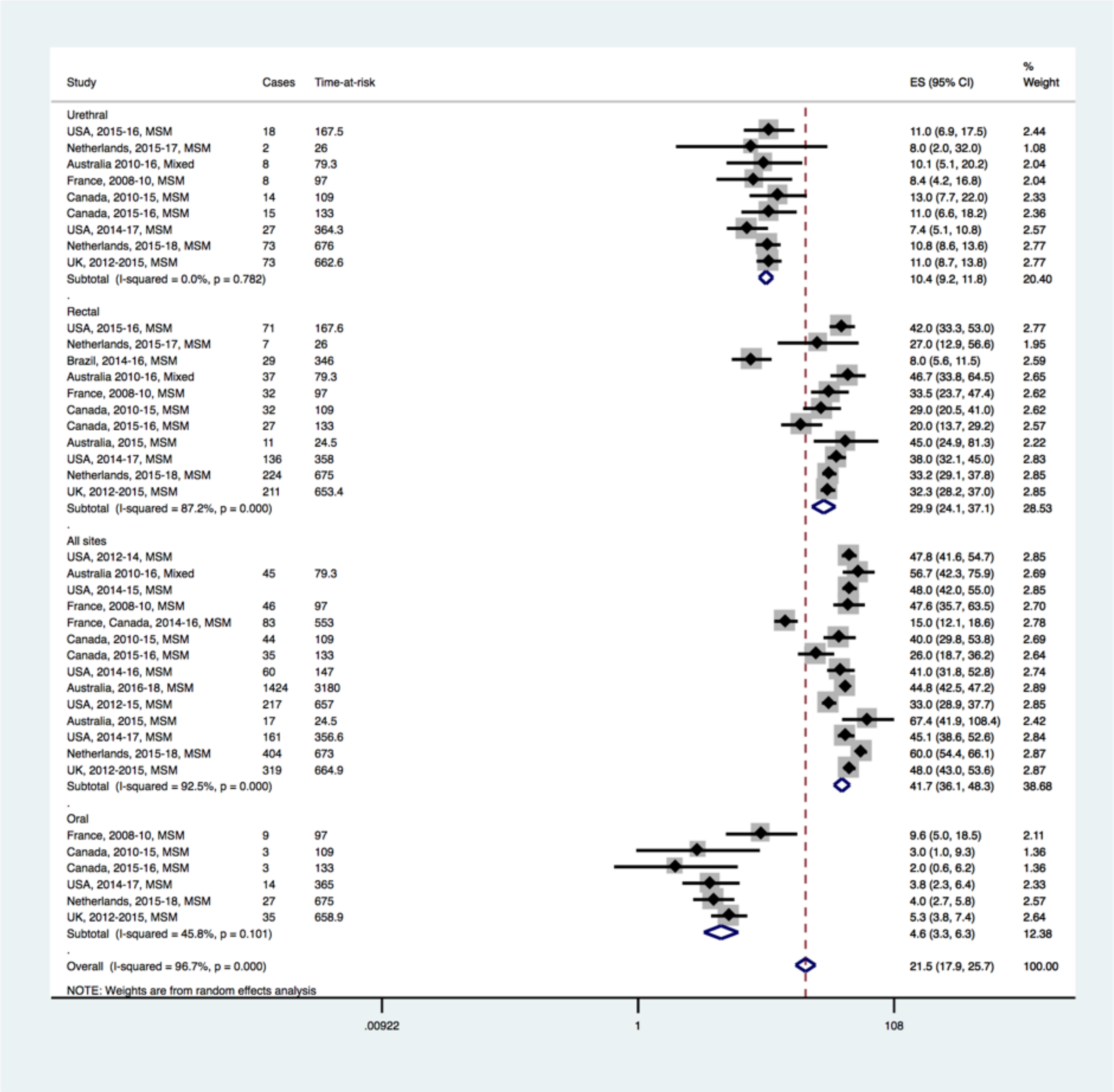

© 2019 Ong JJ et al. *JAMA Network Open.*

eFigure 7, panel C - Random effects meta-analysis of chlamydia incidence by MSM status of the study population

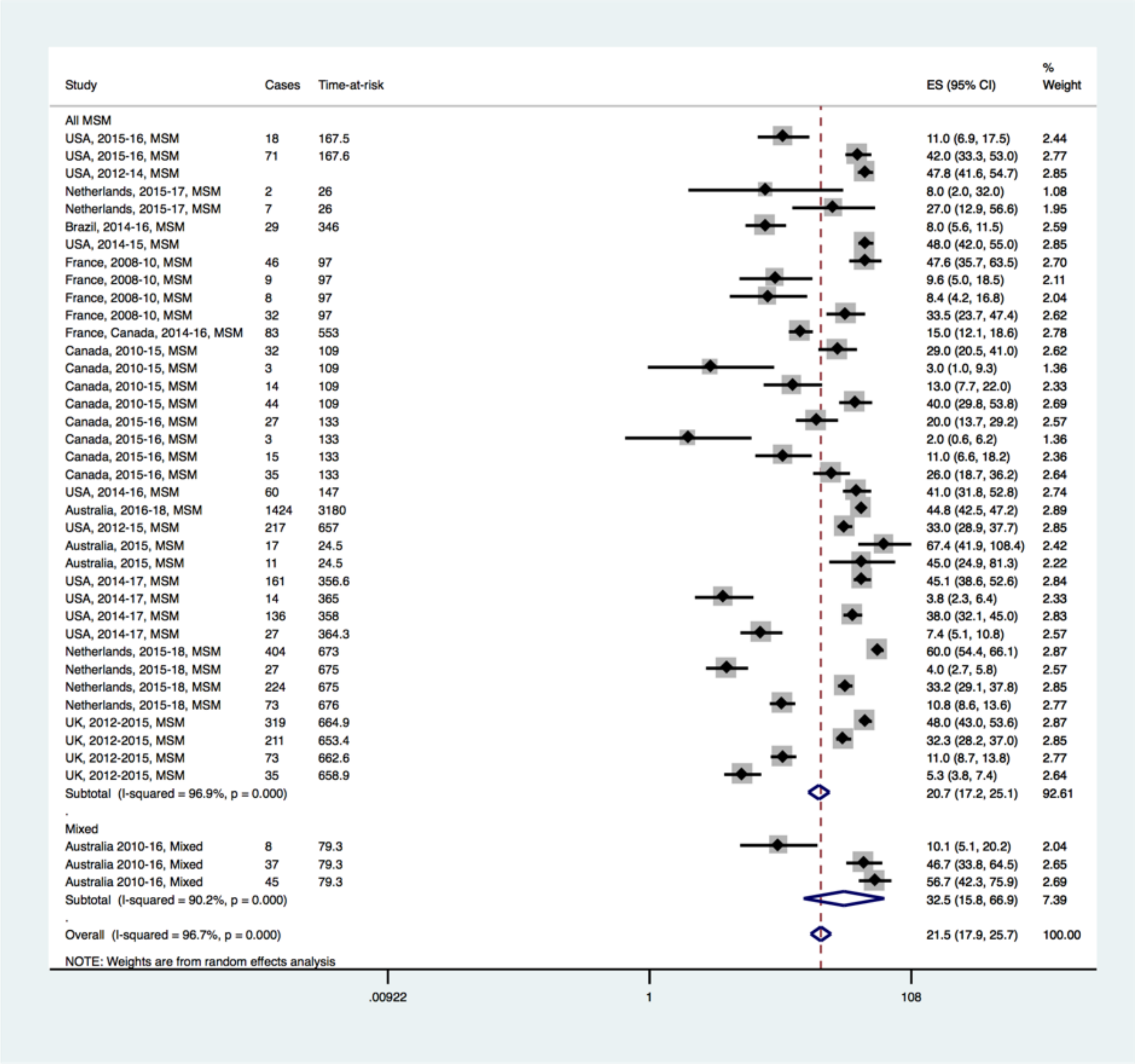

© 2019 Ong JJ et al. *JAMA Network Open.*

eFigure 7, panel D - Random effects meta-analysis of chlamydia incidence by country income level

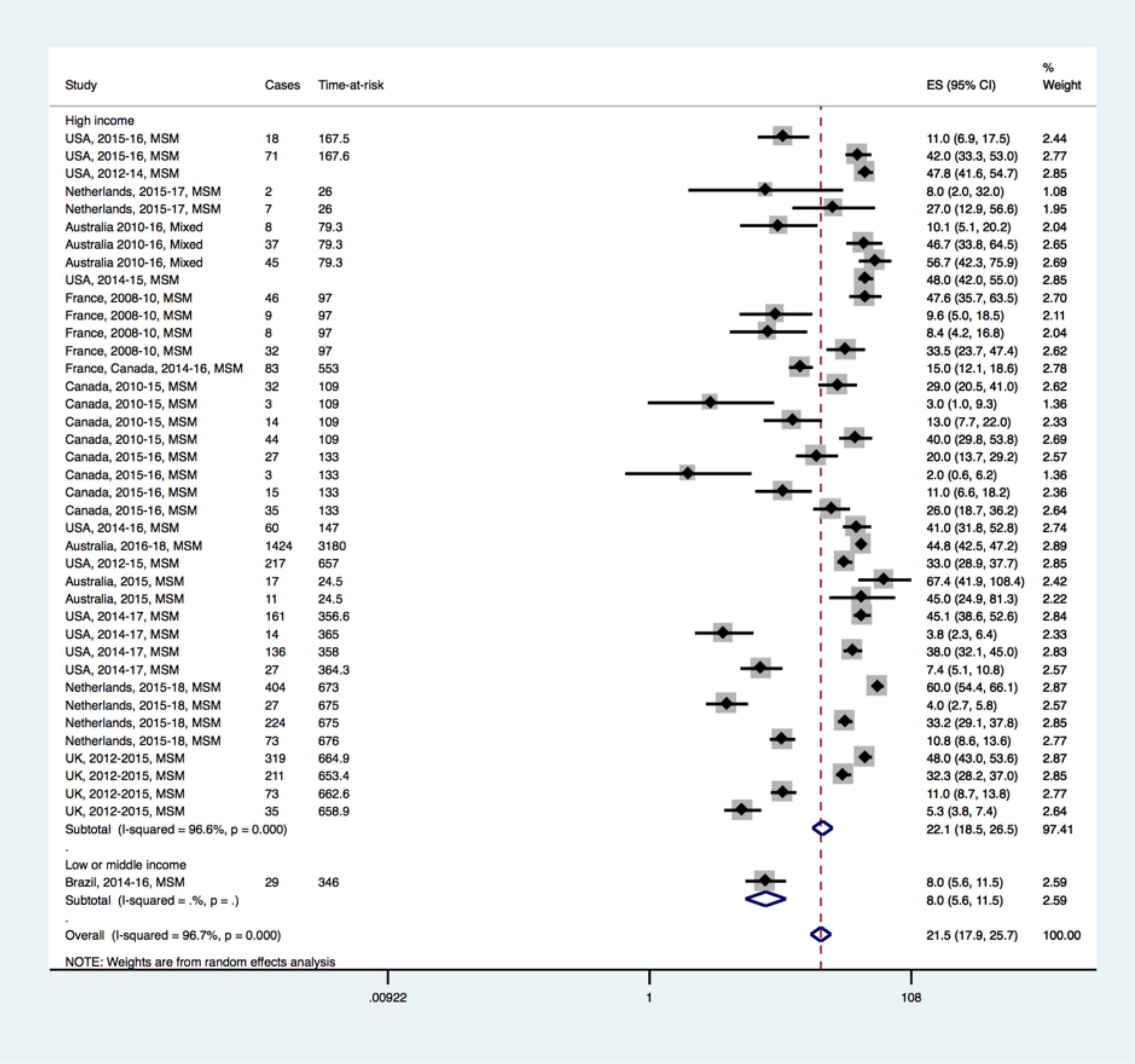

© 2019 Ong JJ et al. *JAMA Network Open.*

eFigure 7, panel E- Random effects meta-analysis of chlamydia incidence by study type

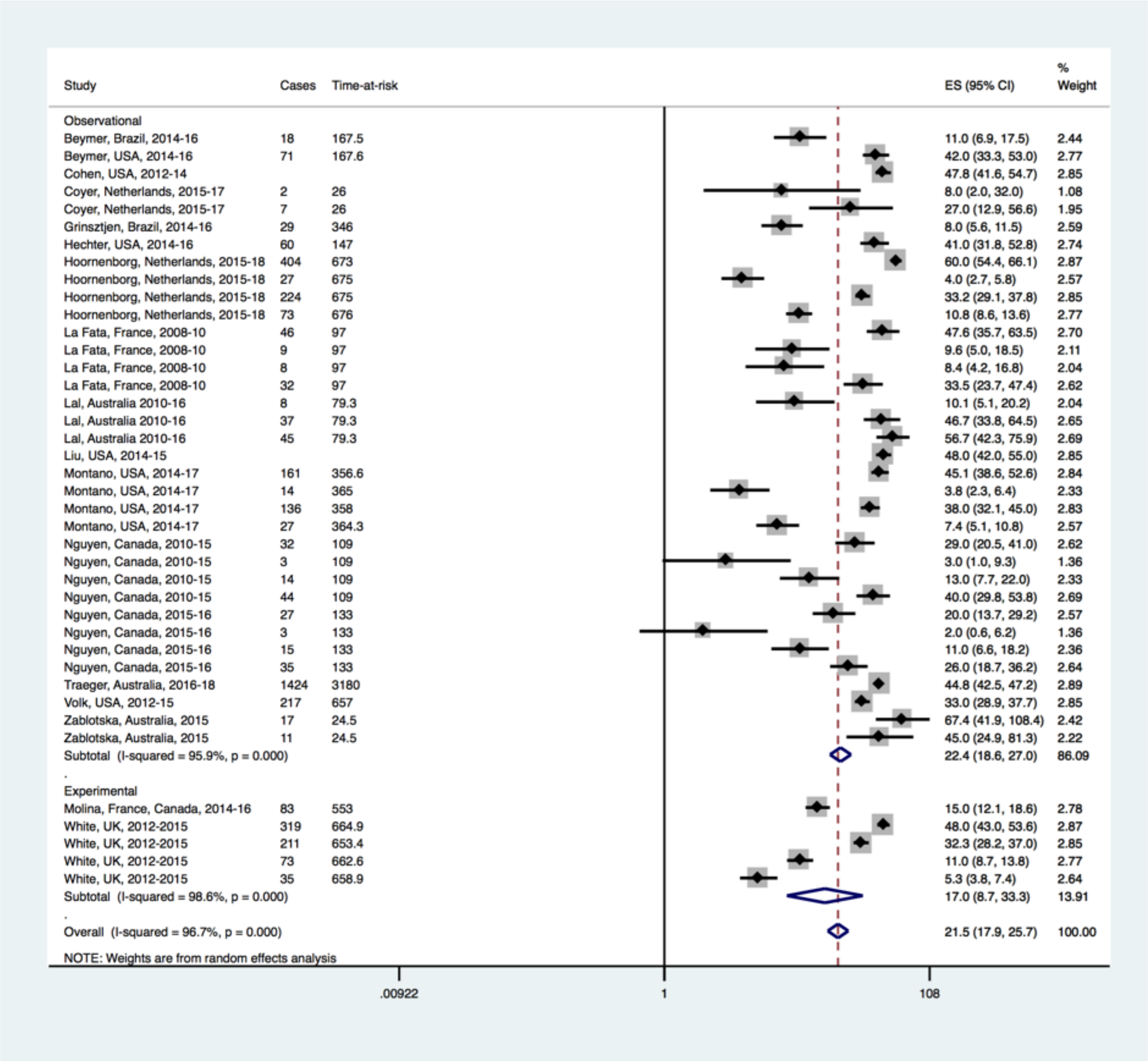

© 2019 Ong JJ et al. *JAMA Network Open.*

eFigure 7, panel F - Random effects meta-analysis of chlamydia incidence by publication status

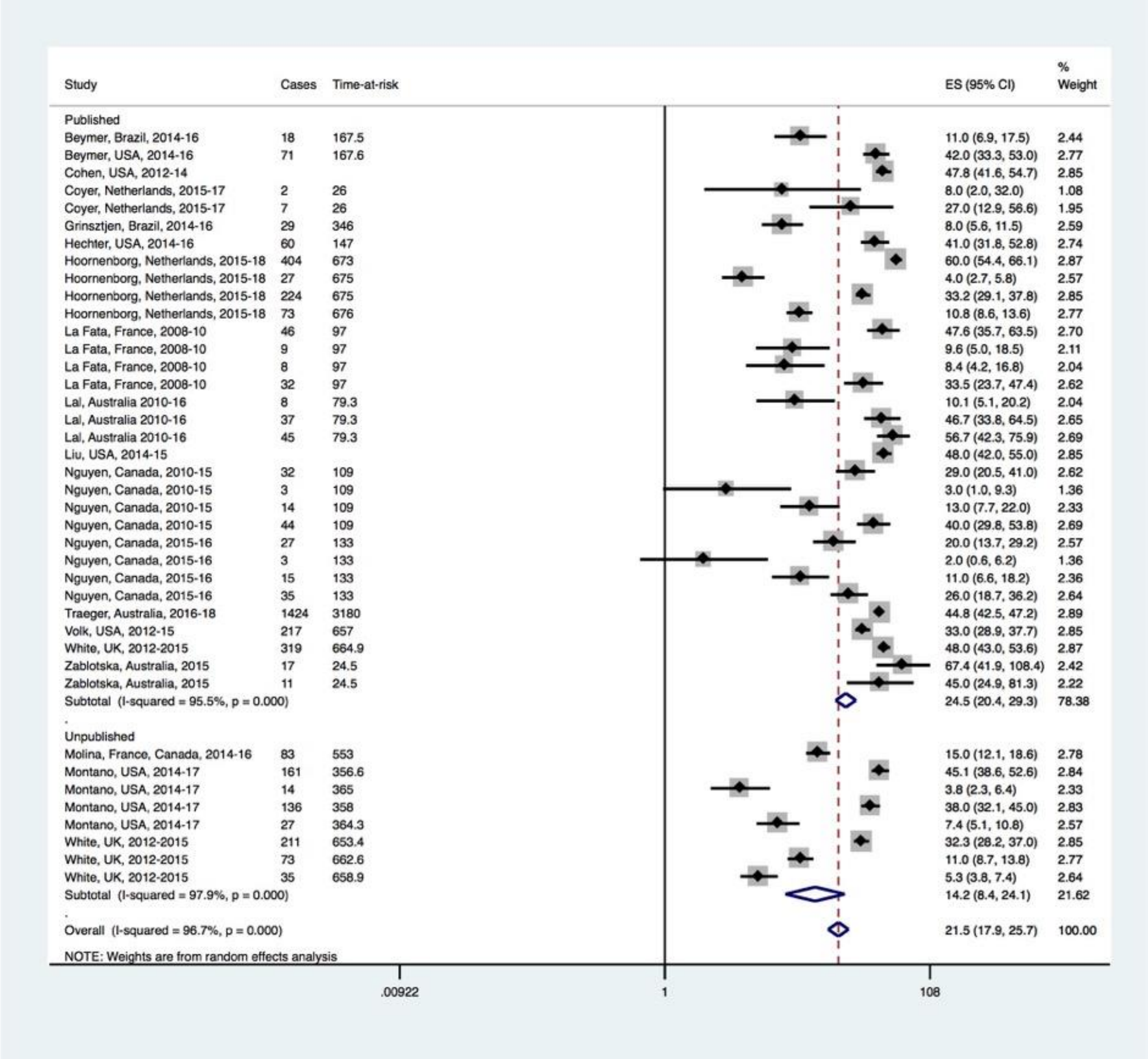

© 2019 Ong JJ et al. *JAMA Network Open.*

eTable 5. Meta-Regression Results for the Predictors of Chlamydia Incidence and Sources of Between-Study Heterogeneity

Characteristic	Variable	OR (95% CI)	p value	AOR (95% CI)	p value
Anatomical site	Oral	1		1	
	All sites	2.98 (1.78-4.97)	<0.001	8.64 (5.88-12.69)	<0.001
	Genital	0.42 (0.22-0.81)	0.01	2.16 (1.41-3.31)	0.001
	Anorectal	1.81 (0.96-3.39)	0.07	7.13 (4.77-10.67)	<0.001
Population	Mixed	1		1	
	MSM only	0.60 (0.20-1.80)	0.35	0.77 (0.50-1.19)	0.23
Country income level	LMIC	1		1	
	High	2.50 (0.40-15.46)	0.32	4.22 (2.08-8.56)	<0.001
Study type	Observational	1		1	
	Experimental	0.85 (0.36-2.03)	0.71	0.87 (0.64-1.17)	0.34

AOR = adjusted odds ratio; LMIC = low-middle income country; MSM = men who have sex with men; OR = odds ratio; RCT = randomized controlled trial

© 2019 Ong JJ et al. *JAMA Network Open.*

eFigure 7, panel G - Funnel plot and Egger's for chlamydia incidence

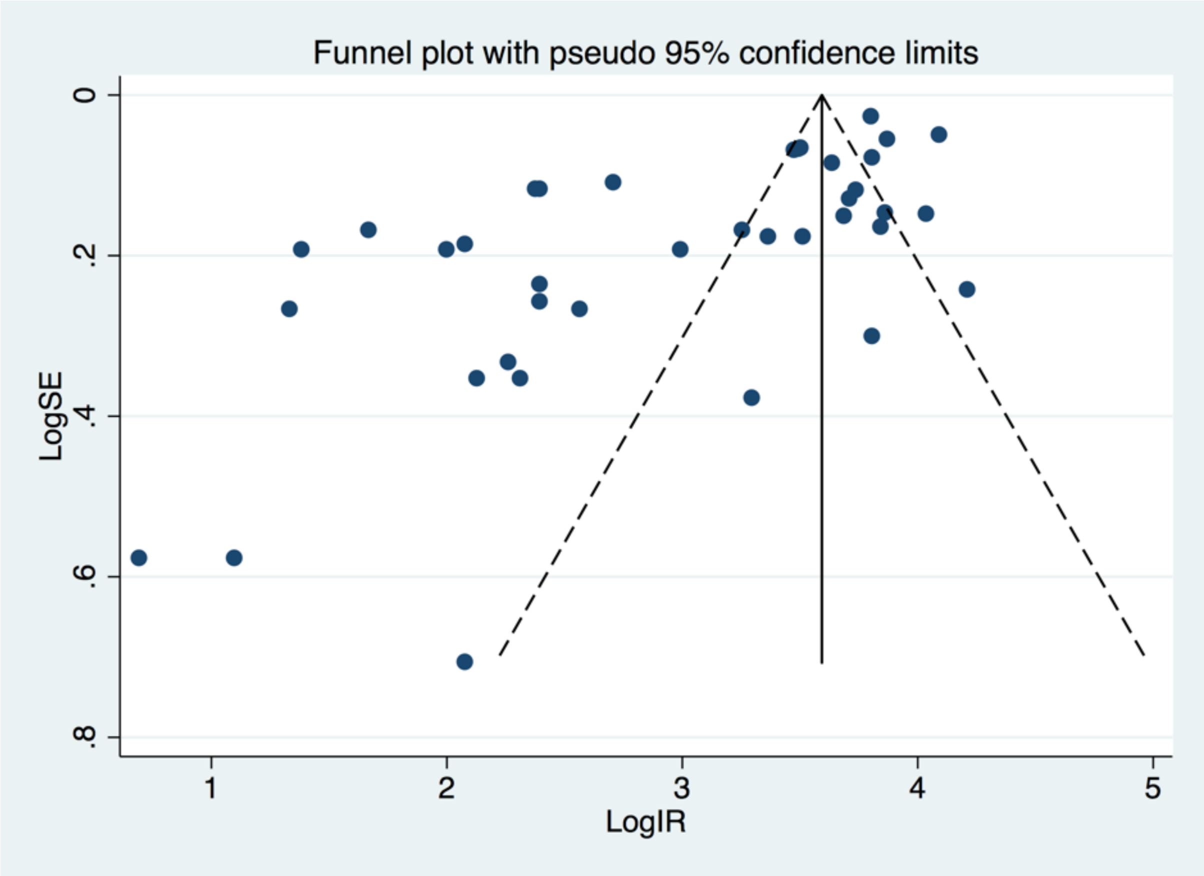

Egger's test – p<0.001

eFigure 8. Random Effects Meta-Analysis of Gonorrhea Incidence
panel A - Random effects meta-analysis of gonorrhoea incidence

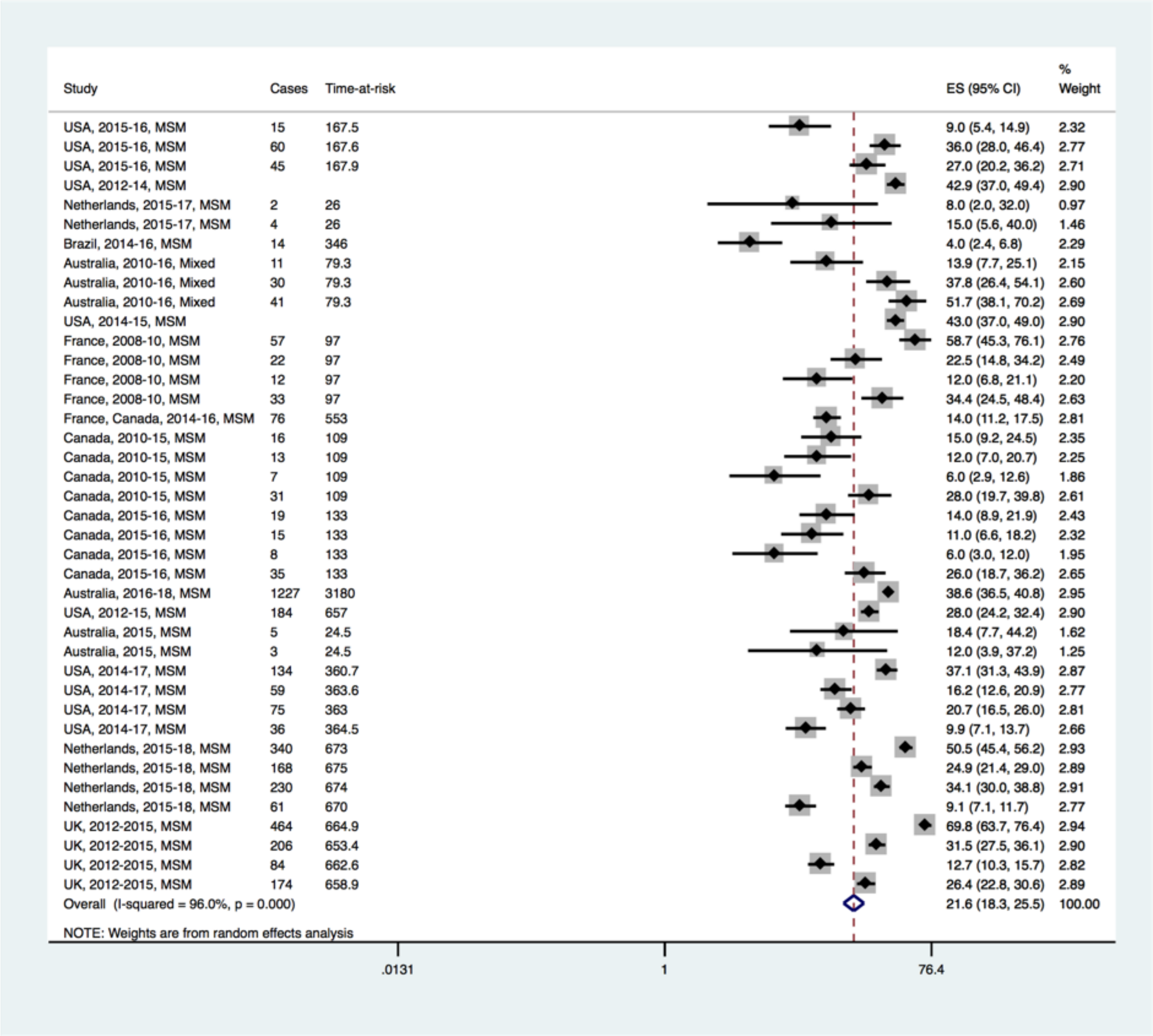

© 2019 Ong JJ et al. *JAMA Network Open.*

eFigure 8, panel B - Random effects meta-analysis of gonorrhoea incidence by anatomical site

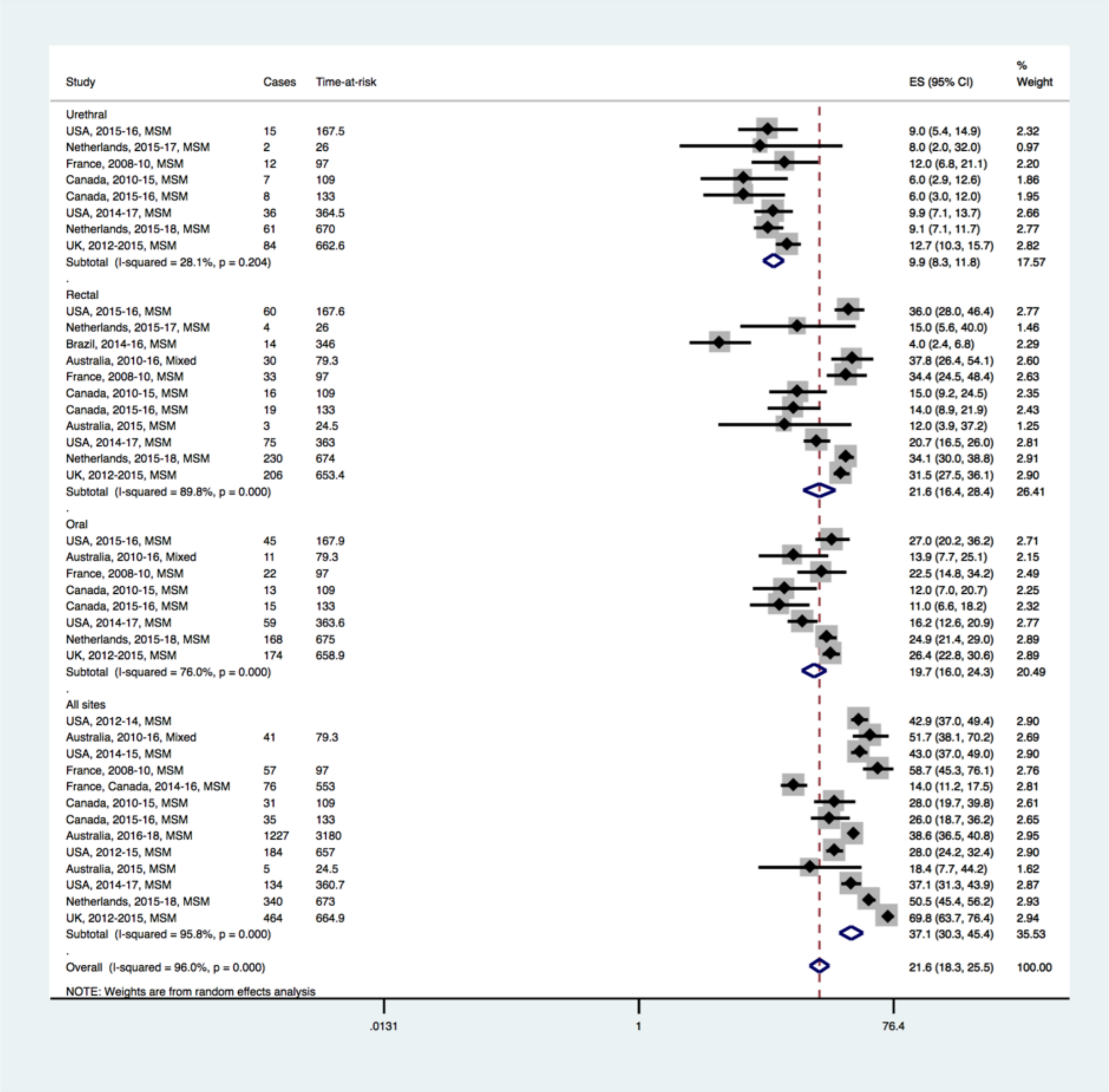

© 2019 Ong JJ et al. *JAMA Network Open.*

eFigure 8, panel C - Random effects meta-analysis of gonorrhoea incidence by MSM status of the study population

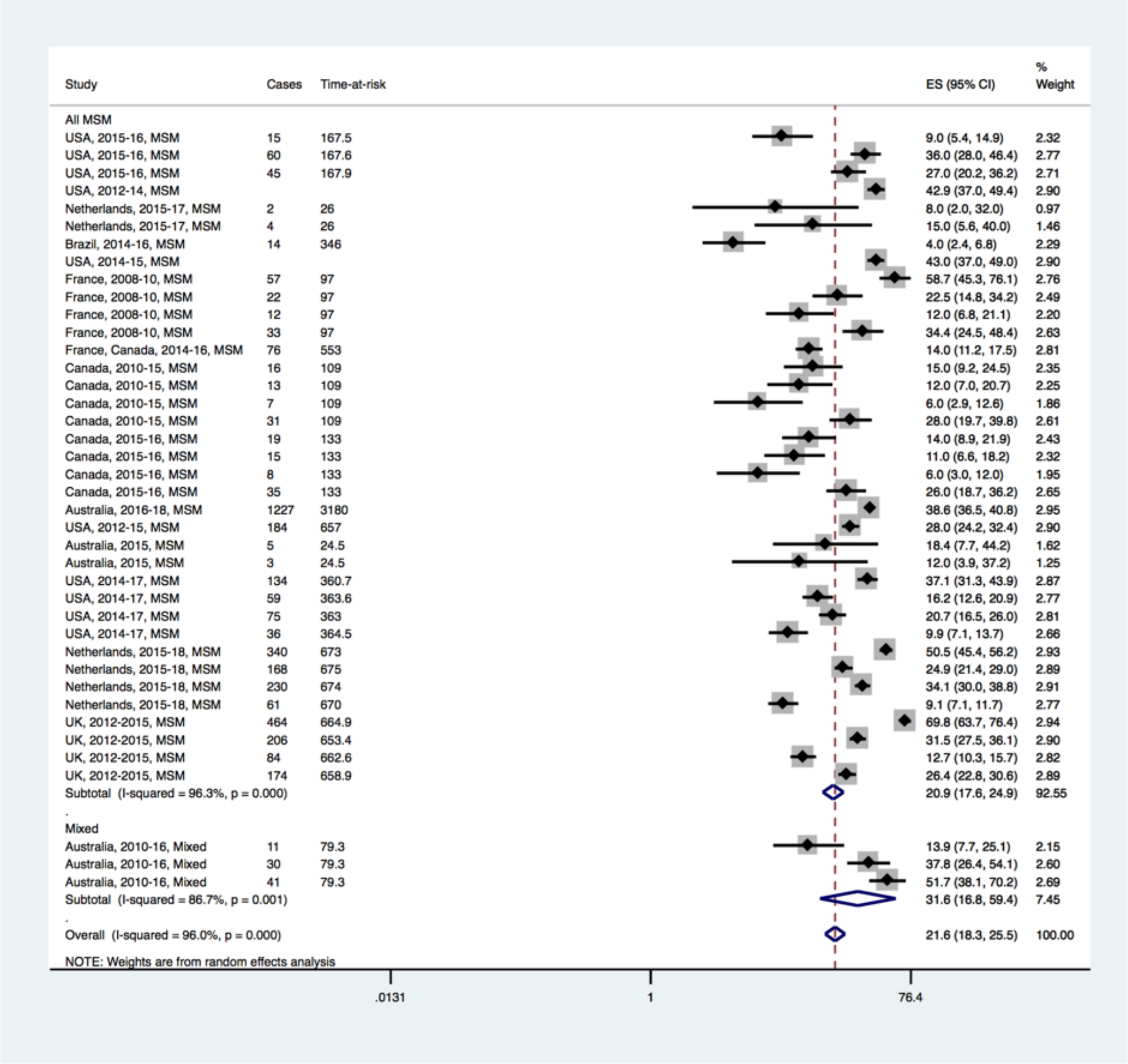

© 2019 Ong JJ et al. *JAMA Network Open.*

eFigure 8, panel D - Random effects meta-analysis of gonorrhoea incidence by country income level

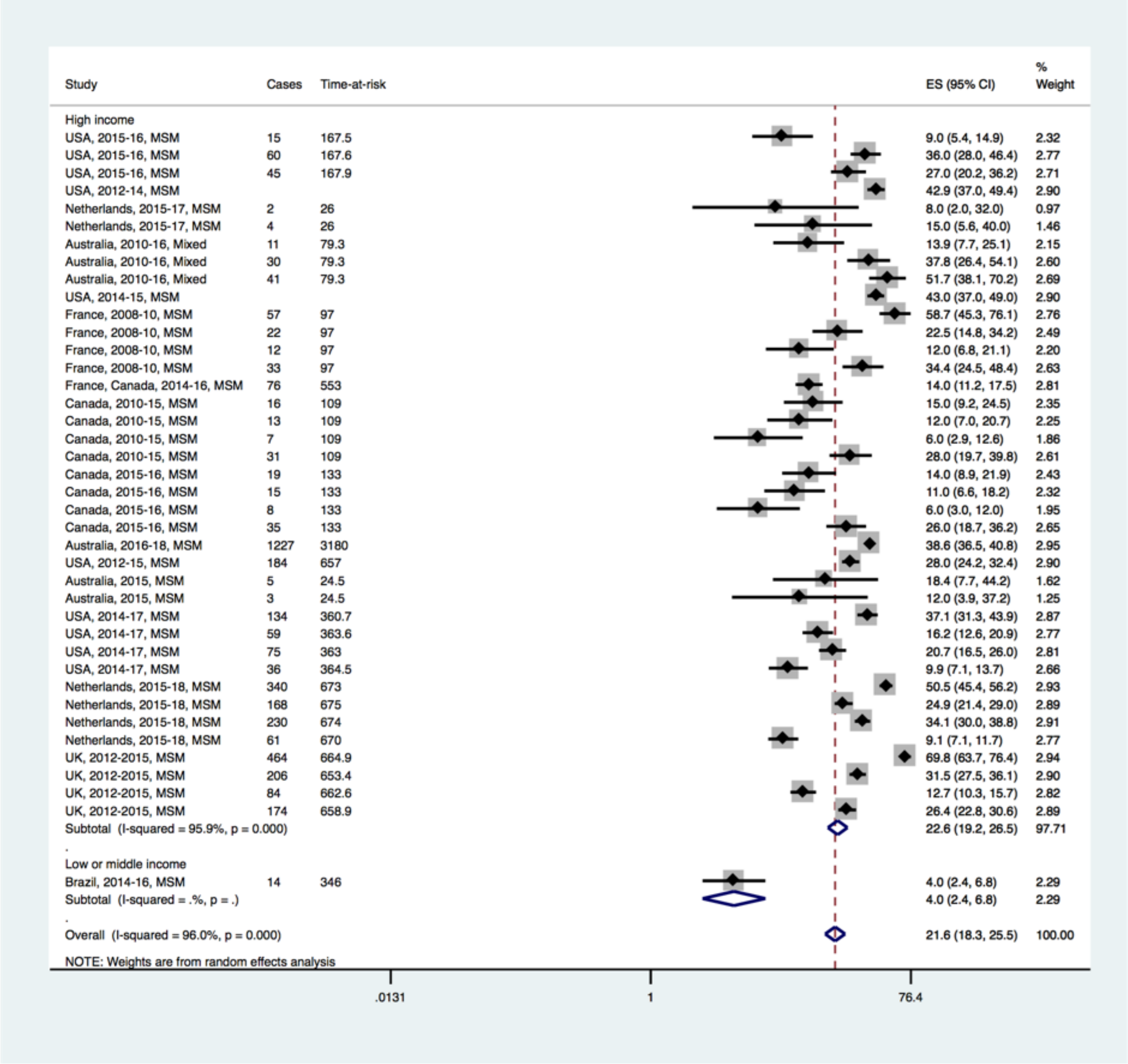

© 2019 Ong JJ et al. *JAMA Network Open.*

eFigure 8, panel E - Random effects meta-analysis of gonorrhoea incidence by study type

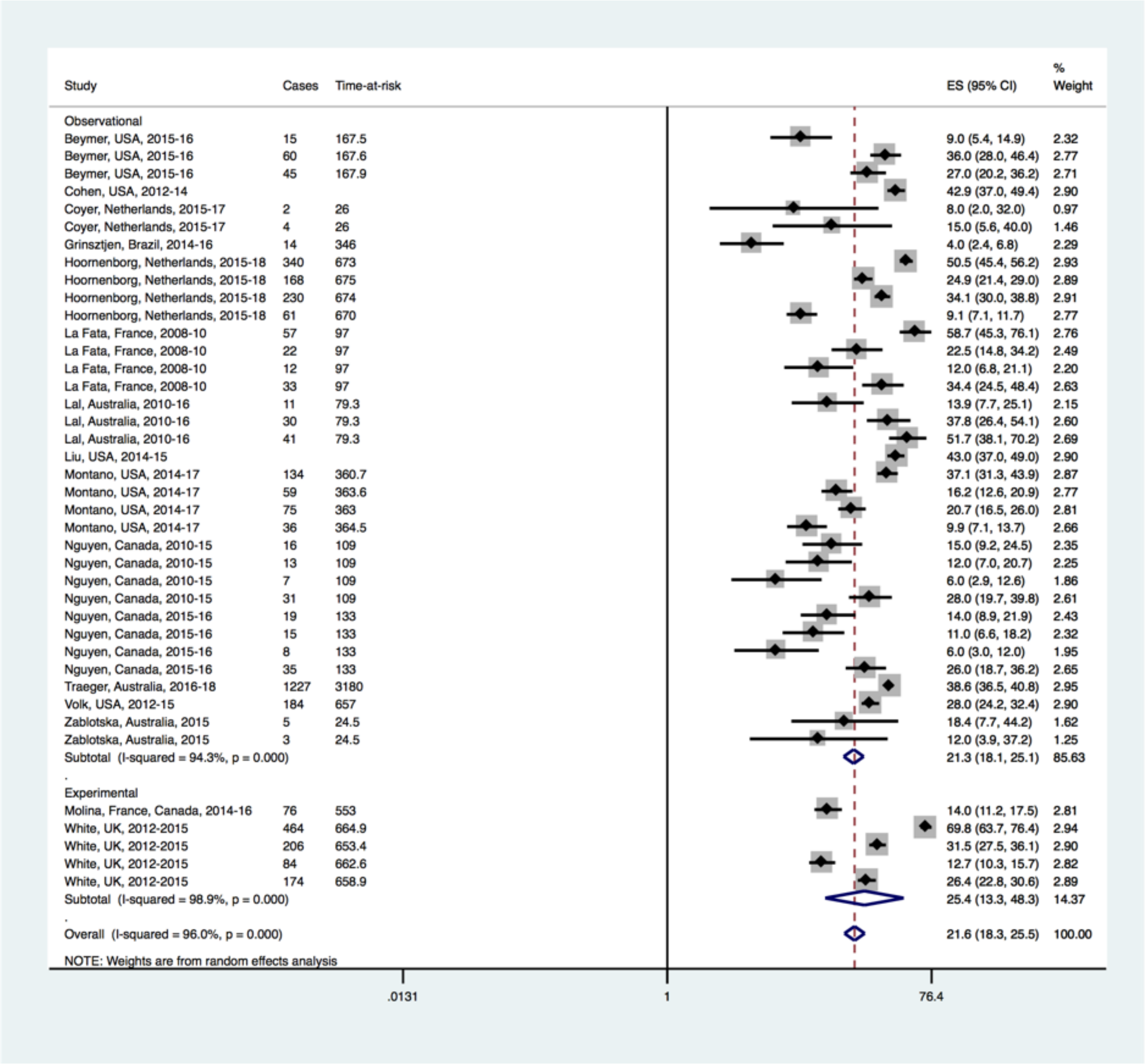

© 2019 Ong JJ et al. *JAMA Network Open.*

eFigure 8, panel F - Random effects meta-analysis of gonorrhoea incidence by publication status

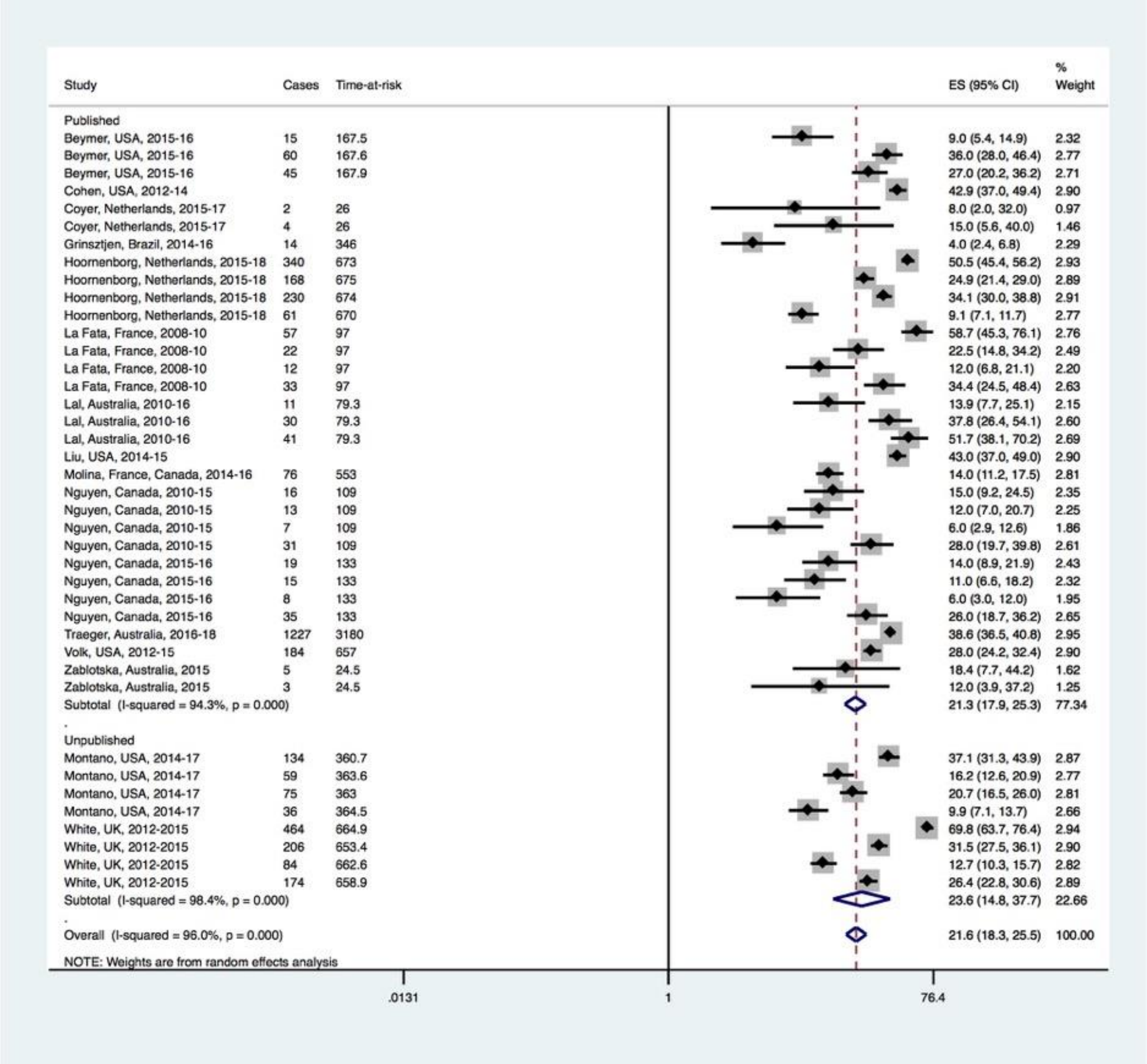

© 2019 Ong JJ et al. *JAMA Network Open.*

eTable 6. Meta-Regression Results for the Predictors of Gonorrhea Incidence and Sources of Between-Study Heterogeneity

Characteristic	Variable	OR (95% CI)	p value	AOR (95% CI)	p value
Anatomical site	Oral	1		1	
	All sites	2.20 (1.47-3.31)	<0.001	1.87 (1.27-2.76)	0.002
	Genital	0.37 (0.23-0.58)	<0.001	0.50 (0.32-0.78)	0.003
	Anorectal	1.03 (0.62-1.69)	0.92	1.33 (0.89-2.01)	0.16
Population	Mixed	1		1	
	MSM only	0.63 (0.28-1.42)	0.26	0.78 (0.46-1.33)	0.35
Country income level	LMIC	1		1	
	High	5.30 (1.40-20.06)	0.02	6.03 (2.27-15.98)	0.001
Study type	Observational	1		1	
	Experimental	1.31 (0.69-2.48)	0.40	1.20 (0.81-1.76)	0.35

AOR = adjusted odds ratio; LMIC = low-middle income country; MSM = men who have sex with men; OR = odds ratio; RCT = randomized controlled trial

© 2019 Ong JJ et al. *JAMA Network Open.*

eFigure 8, panel G – Funnel plot and Egger's test of gonorrhoea incidence

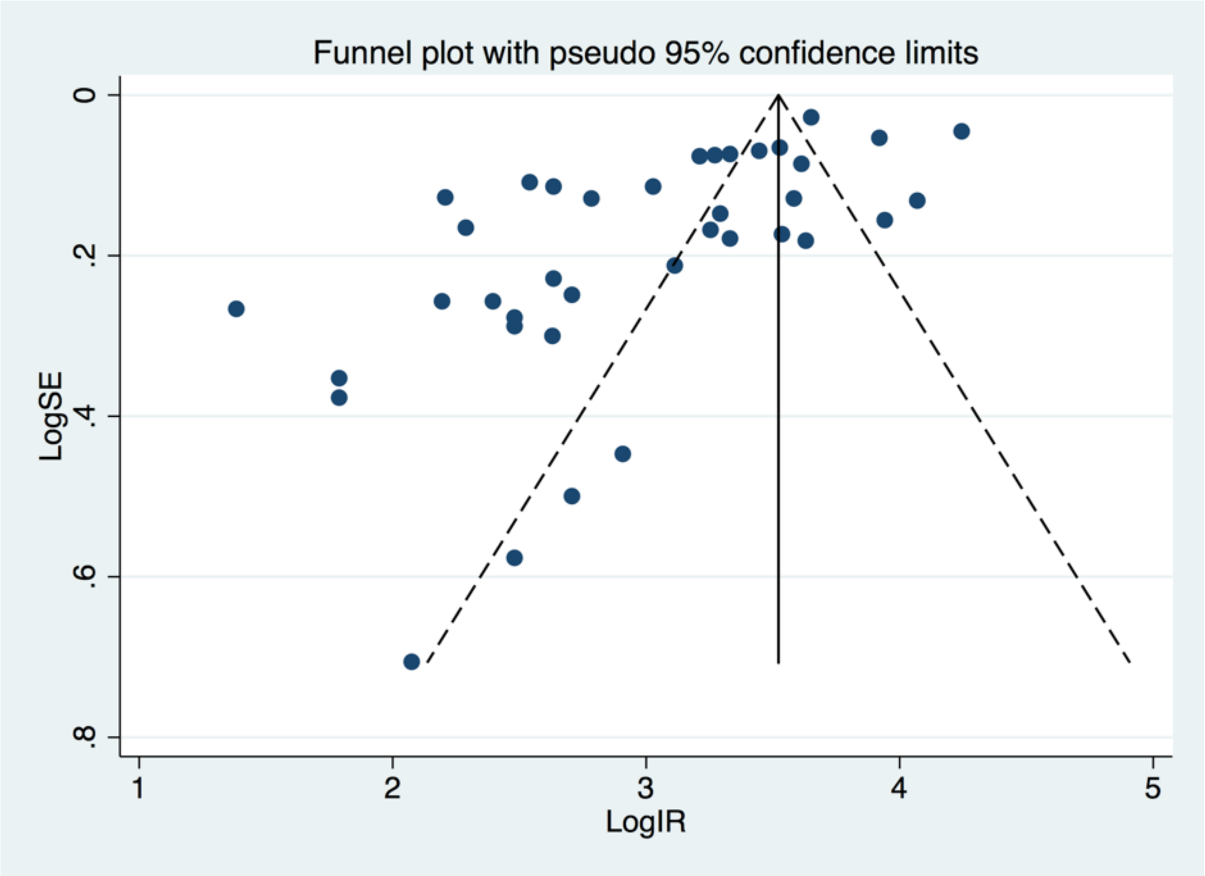

Egger's test – p<0.001

© 2019 Ong JJ et al. *JAMA Network Open.*

eFigure 9. Random Effects Meta-Analysis of Early Syphilis Incidence
panel A - Random effects meta-analysis of early syphilis incidence

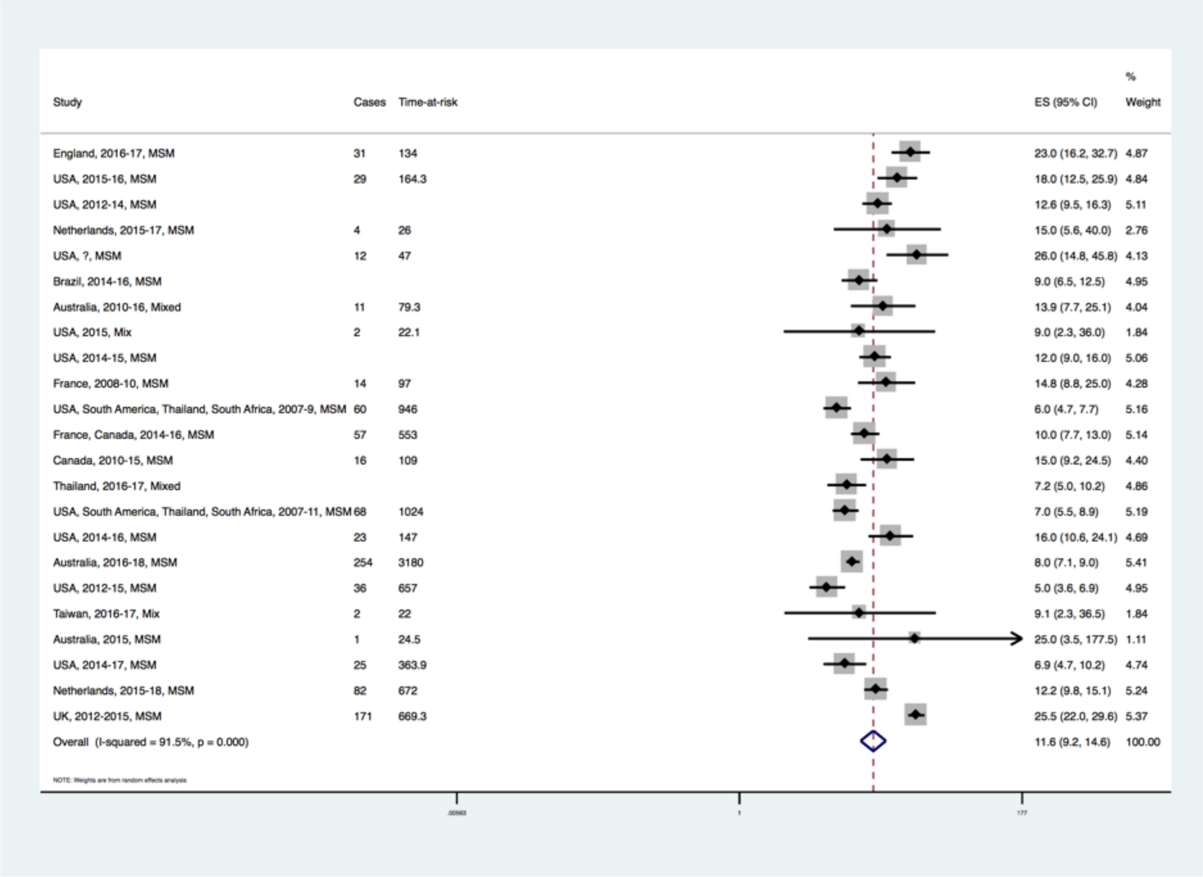

© 2019 Ong JJ et al. *JAMA Network Open.*

eFigure 9, panel B - Random effects meta-analysis of early syphilis incidence by MSM status of the study population

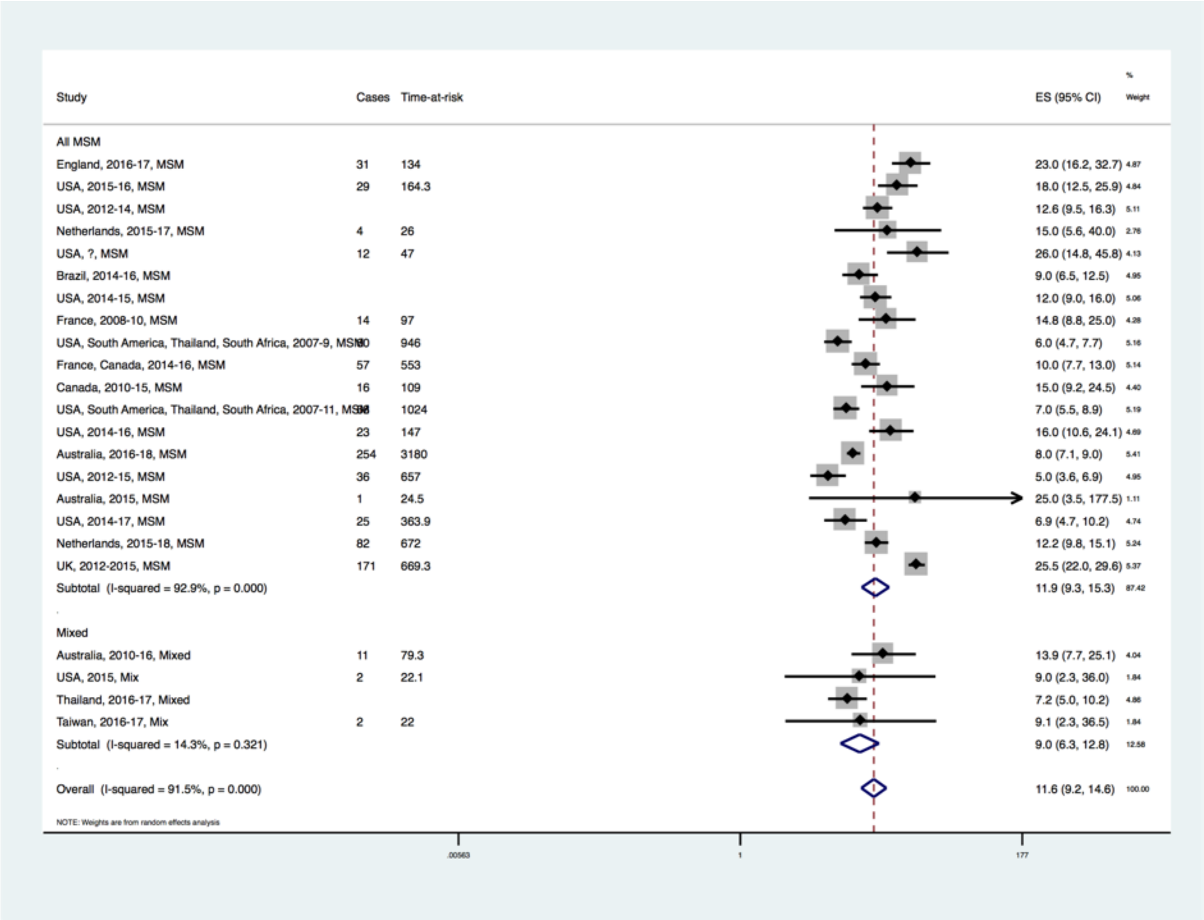

© 2019 Ong JJ et al. *JAMA Network Open.*

eFigure 9, panel C - Random effects meta-analysis of early syphilis incidence by country income level

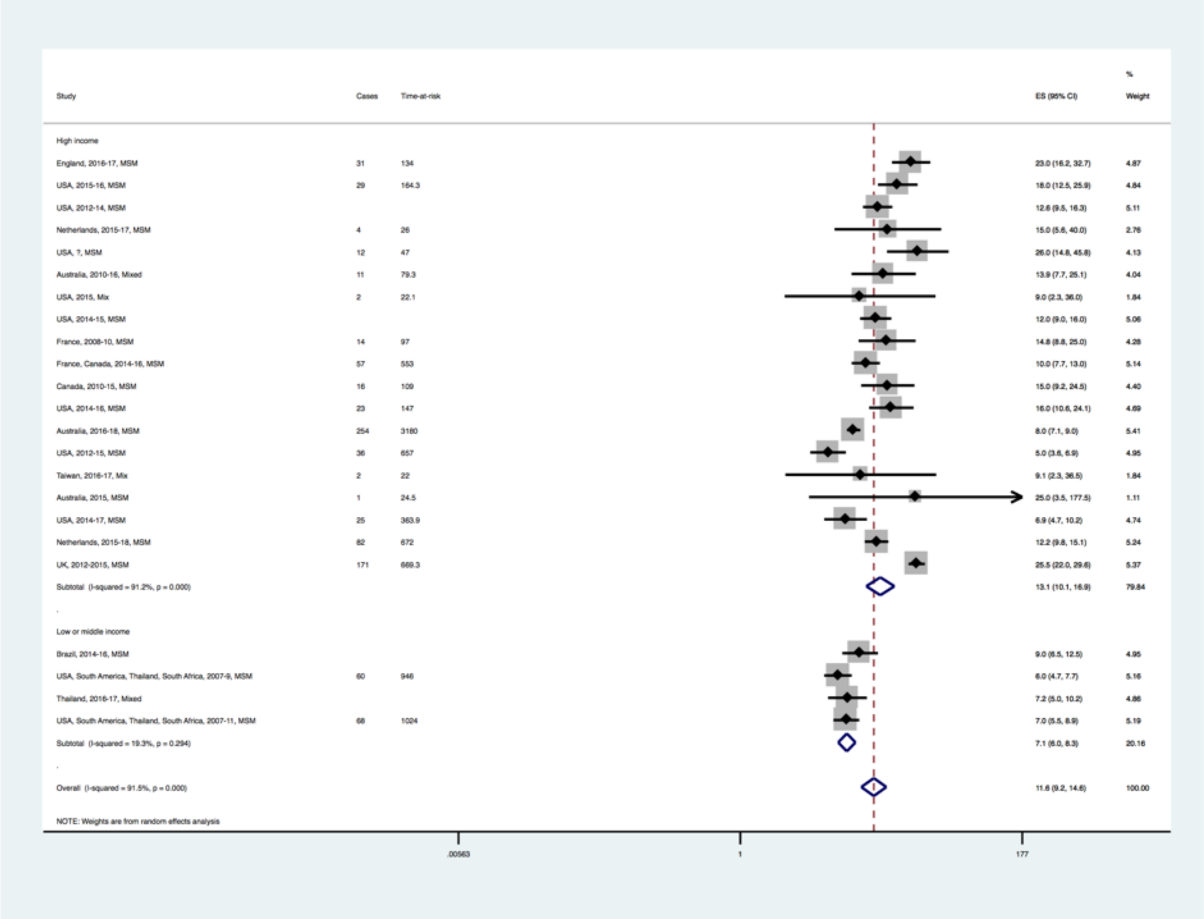

© 2019 Ong JJ et al. *JAMA Network Open.*

eFigure 9, panel D - Random effects meta-analysis of early syphilis incidence by study type

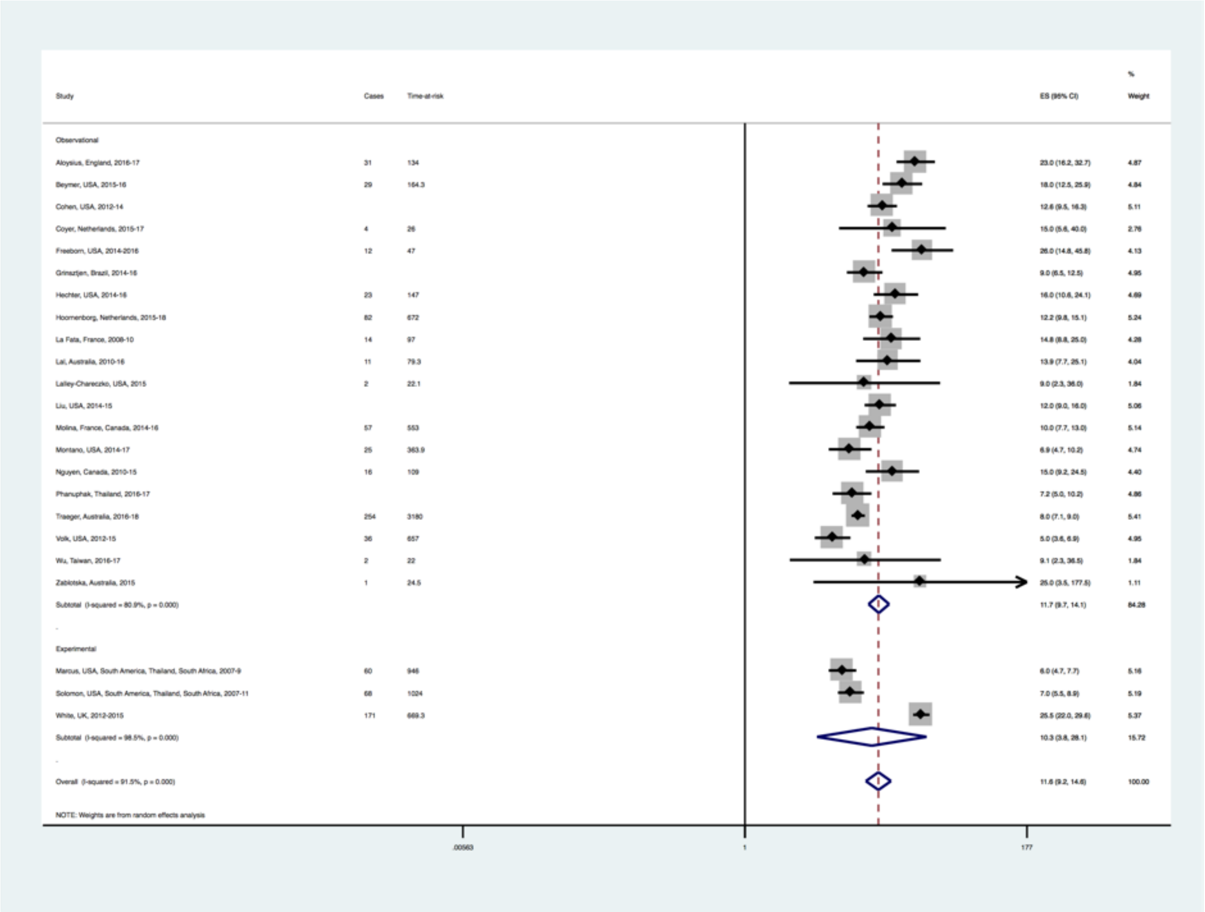

© 2019 Ong JJ et al. *JAMA Network Open.*

eFigure 9, panel E - Random effects meta-analysis of early syphilis incidence by publication status

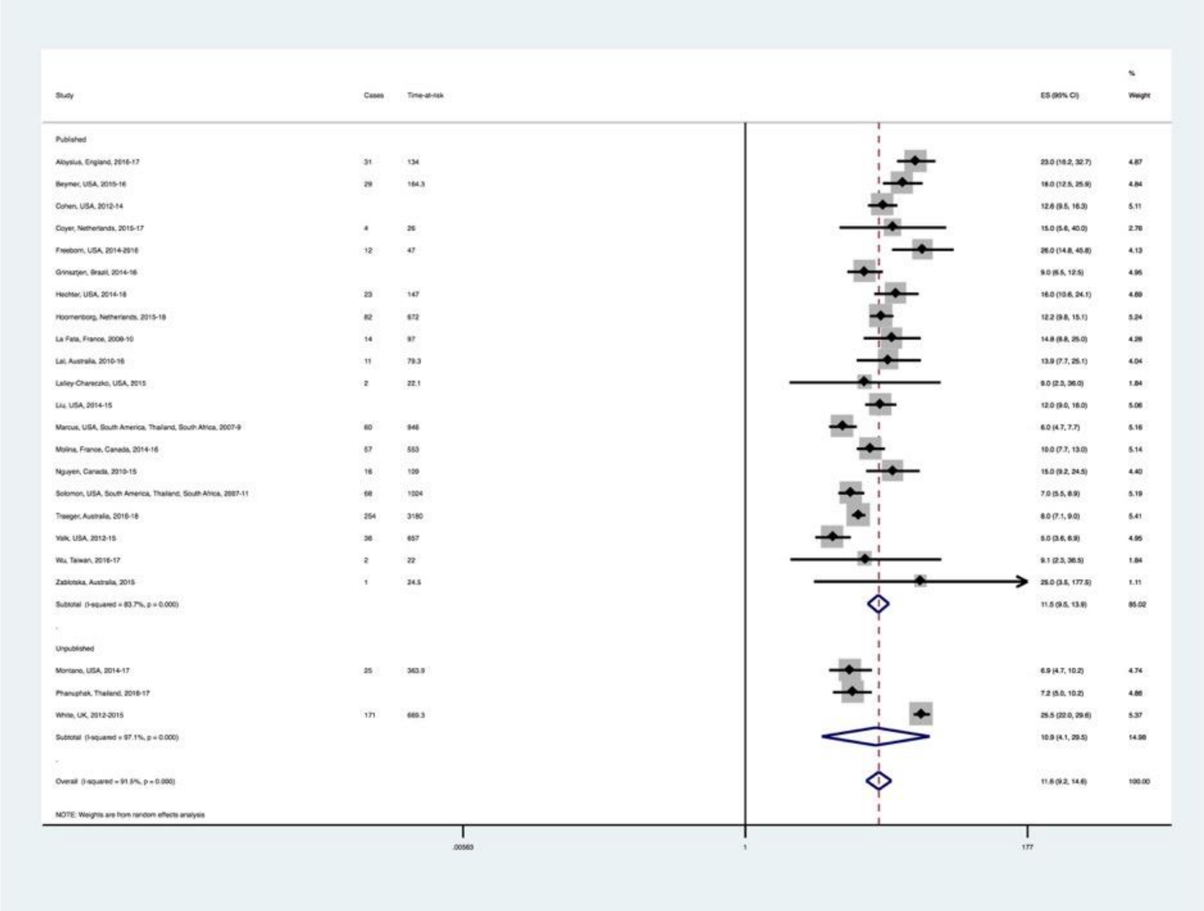

© 2019 Ong JJ et al. *JAMA Network Open.*

eTable 7. Meta-Regression Results for the Predictors of Early Syphilis Incidence and Sources of Between-Study Heterogeneity

Characteristic	Variable	OR (95% CI)	p value	AOR (95% CI)	p value
Population	Mixed	1		1	
	MSM only	1.07 (0.42-2.68)	0.89	1.09 (0.46-2.55)	0.84
Country income level	LMIC	1		1	
	High	2.03 (0.99-4.17)	0.05	3.93 (1.32-11.76)	0.02
Study type	Routine	1		1	
	RCT	0.83 (0.42-1.65)	0.58	2.04 (0.80-5.16)	0.13

AOR = adjusted odds ratio; LMIC = low-middle income country; MSM = men who have sex with men; OR = odds ratio; RCT = randomized controlled trial

© 2019 Ong JJ et al. *JAMA Network Open.*

eFigure 9, panel F – Funnel plot and Egger's test for early syphilis incidence

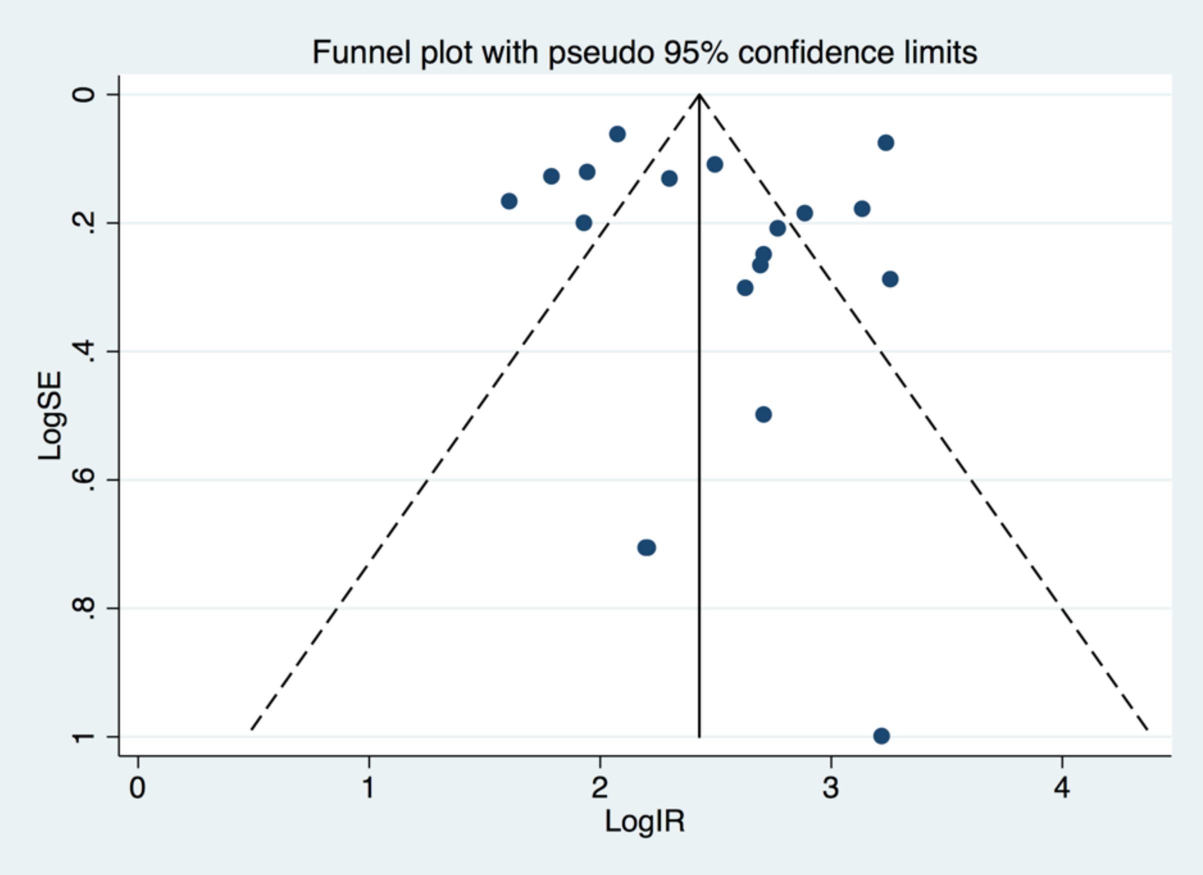

Egger's test – p=0.763

© 2019 Ong JJ et al. *JAMA Network Open.*

eFigure 10. Random Effects Meta-Analysis of Any Chlamydia, Gonorrhea or Early Syphilis Incidence

panel A - Random effects meta-analysis of any chlamydia, gonorrhoea or early syphilis incidence

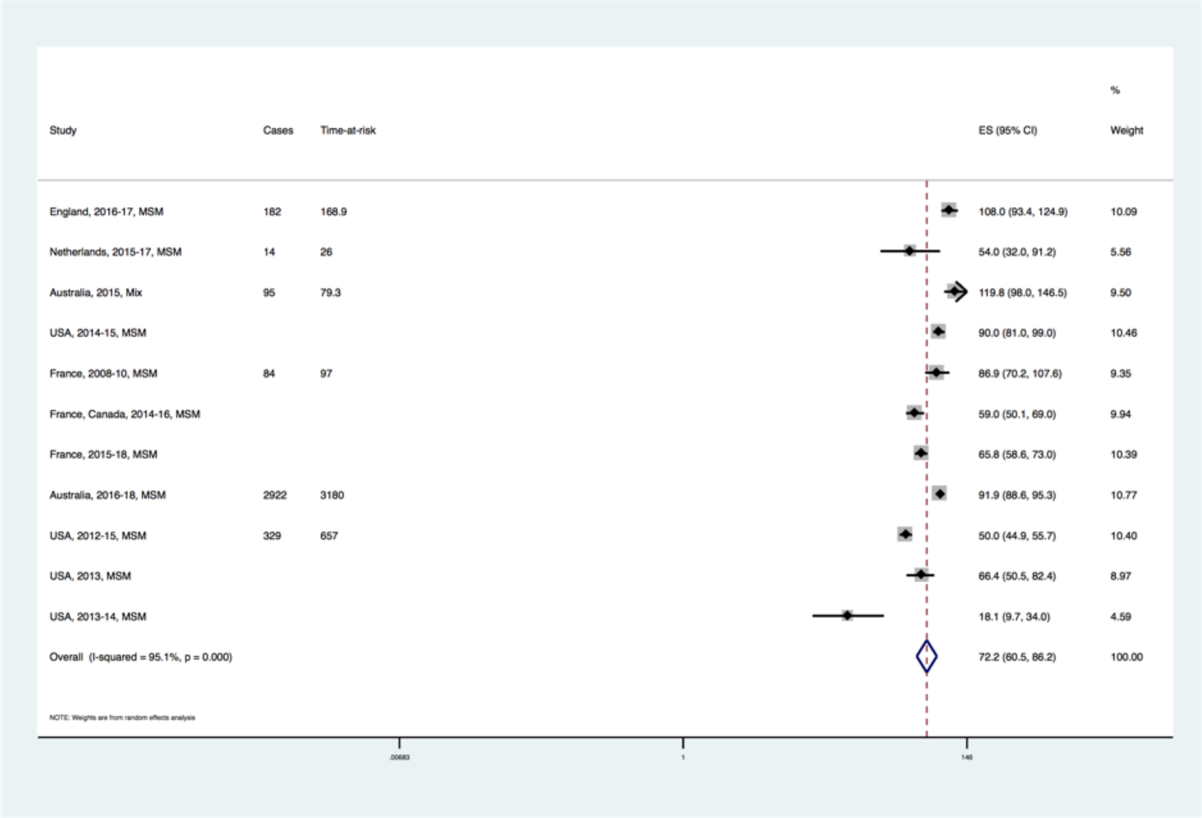

© 2019 Ong JJ et al. *JAMA Network Open.*

eFigure 10, panel B - Random effects meta-analysis of any chlamydia, gonorrhoea or early syphilis incidence by MSM status of the study population

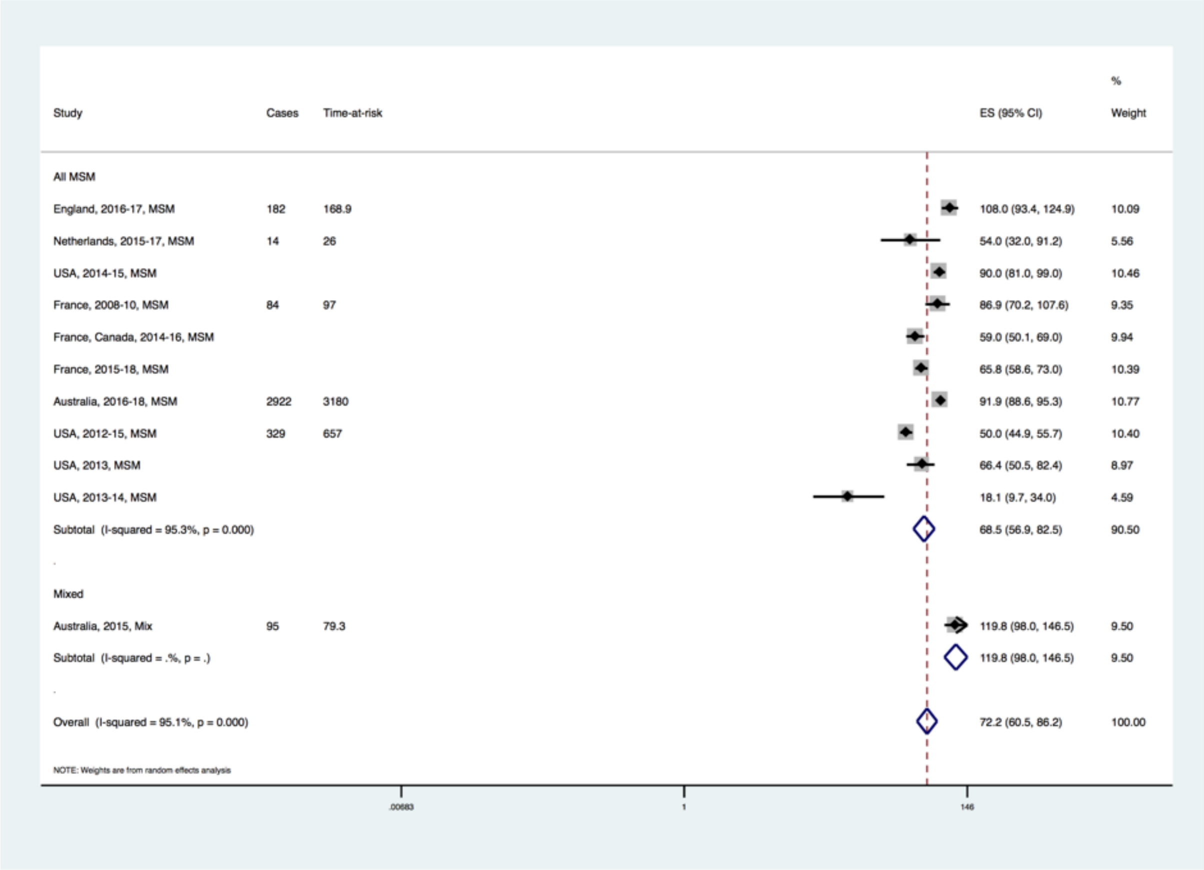

© 2019 Ong JJ et al. *JAMA Network Open.*

eFigure 10, panel C - Random effects meta-analysis of any chlamydia, gonorrhoea or early syphilis incidence by country income level

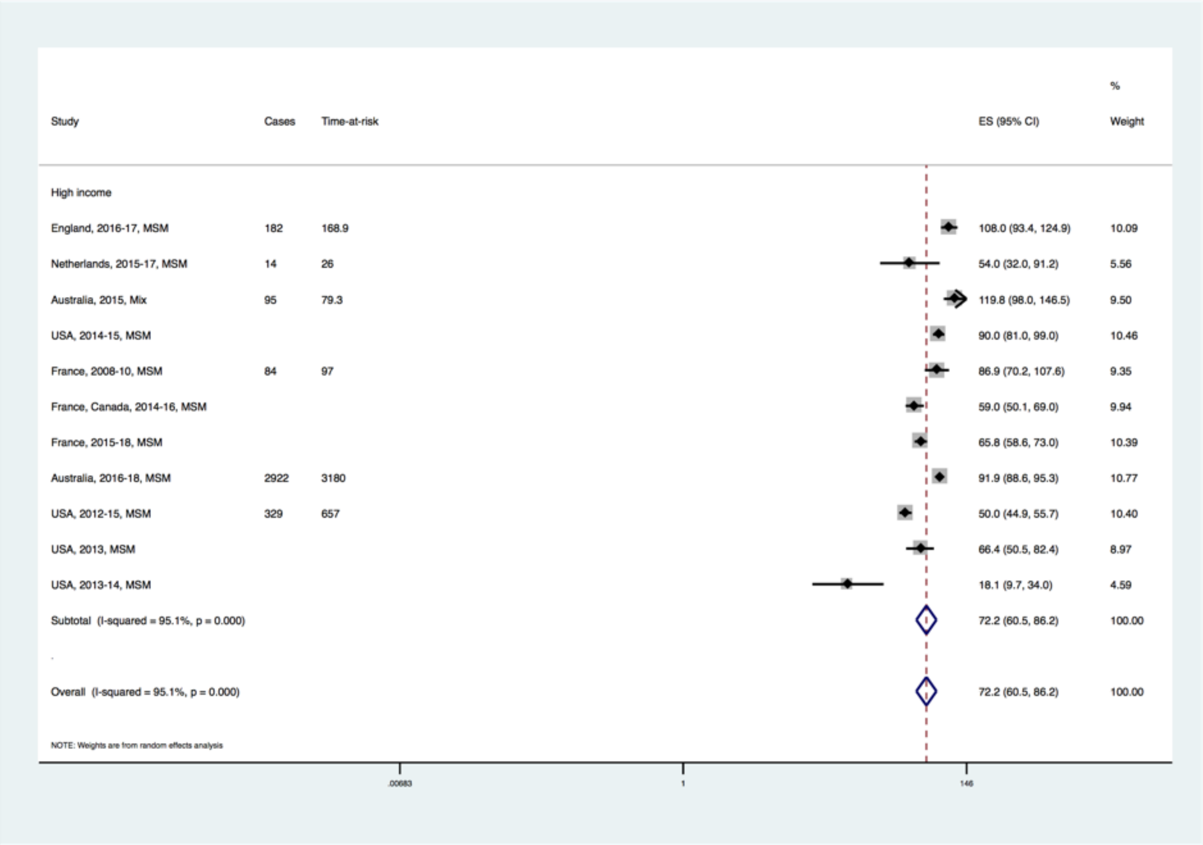

© 2019 Ong JJ et al. *JAMA Network Open.*

eFigure 10, panel D - Random effects meta-analysis of any chlamydia, gonorrhoea or early syphilis incidence by study type

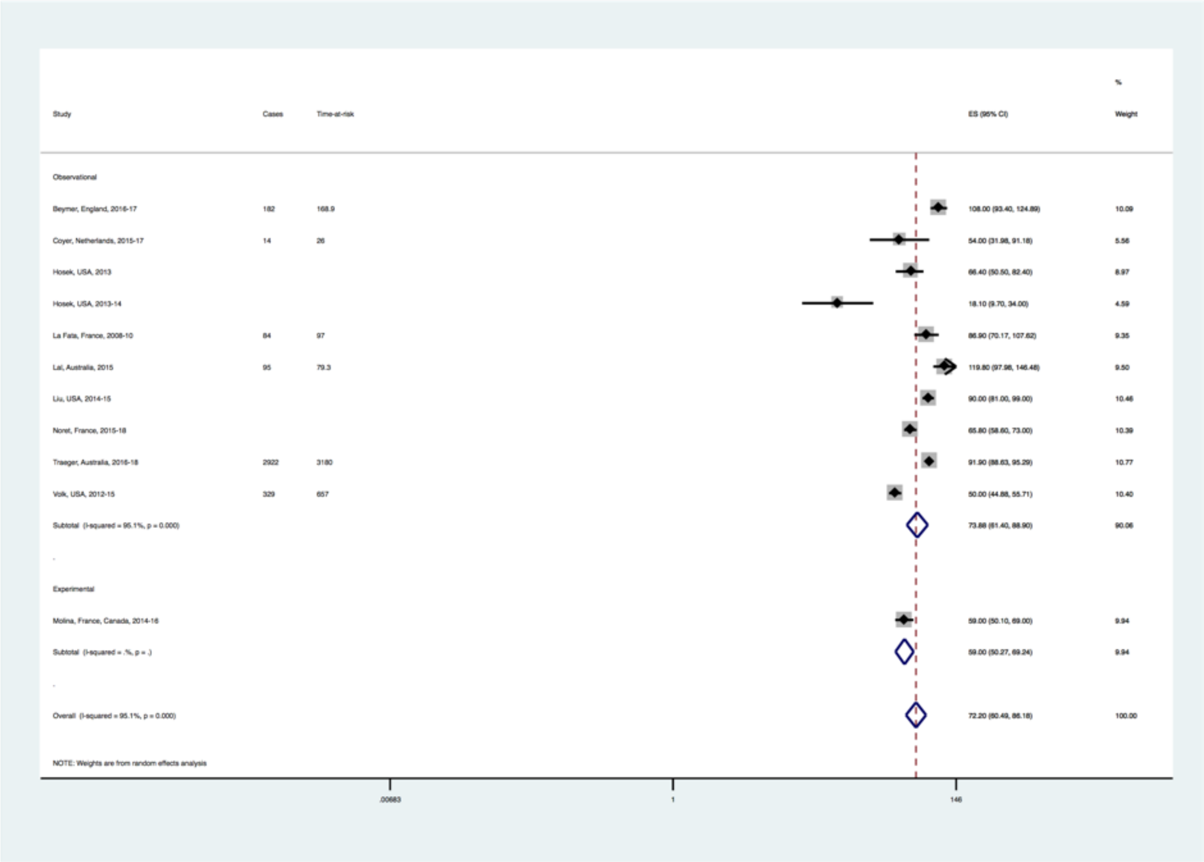

© 2019 Ong JJ et al. *JAMA Network Open.*

eFigure 10, panel E - Random effects meta-analysis of any chlamydia, gonorrhoea or early syphilis incidence by publication status

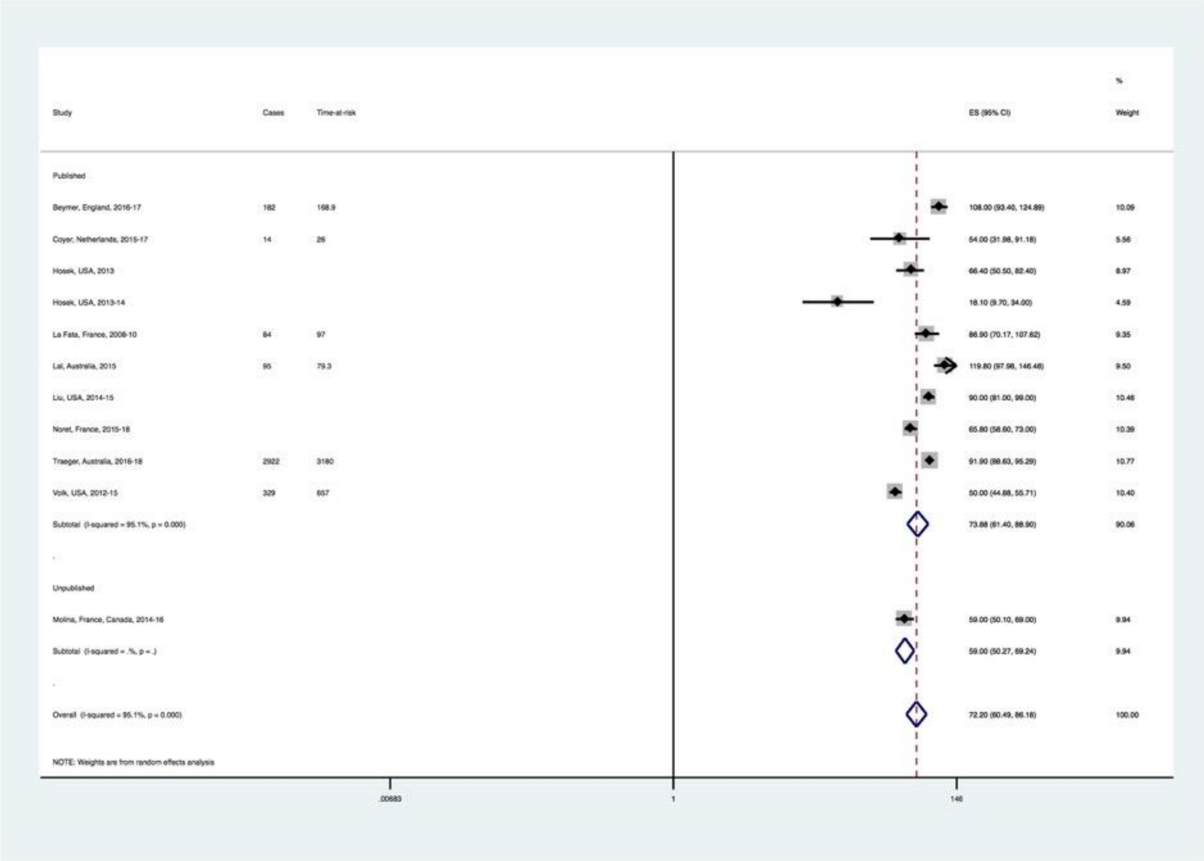

© 2019 Ong JJ et al. *JAMA Network Open.*

eFigure 10, panel F Funnel plot and Egger's test for any chlamydia, gonorrhoea or early syphilis incidence

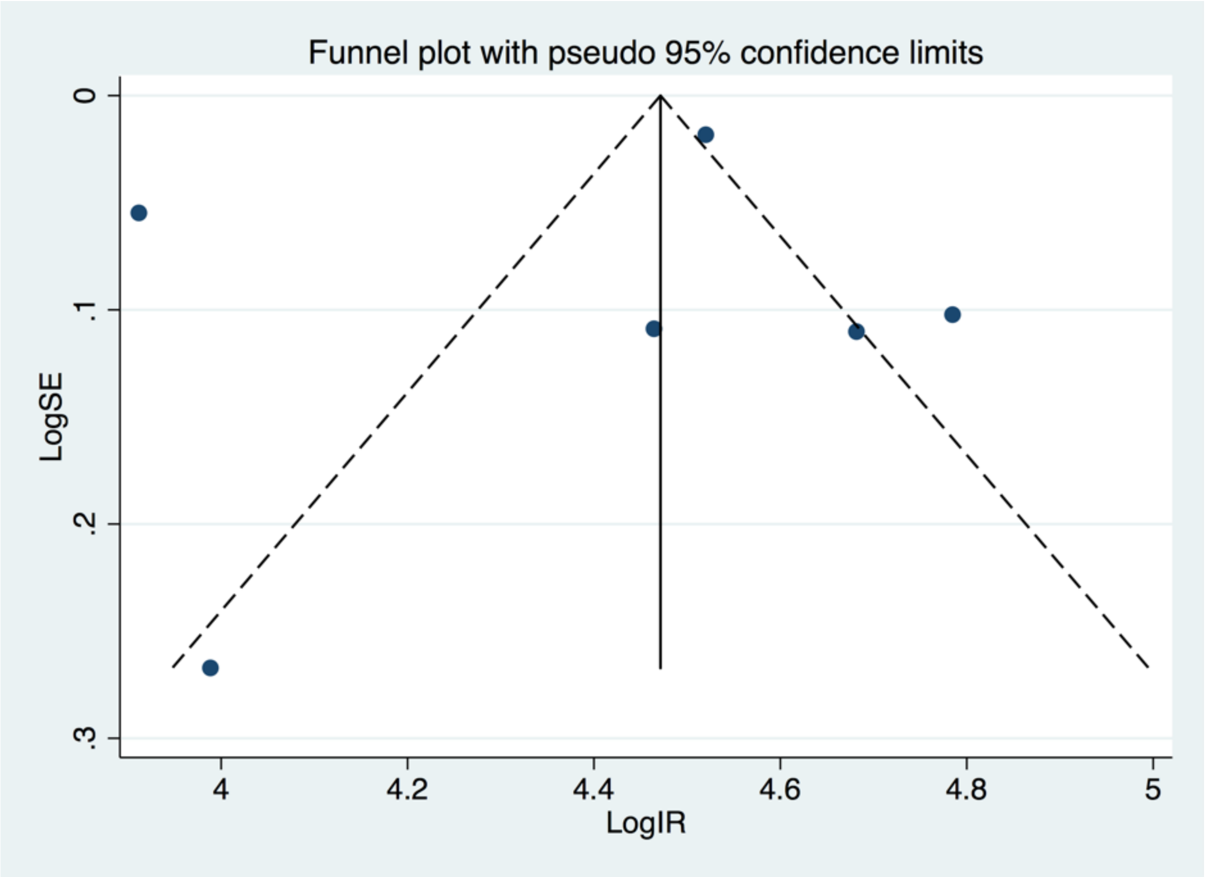

Egger's test = 0.67

© 2019 Ong JJ et al. *JAMA Network Open.*

eFigure 11. Random Effects Meta-Analysis of Hepatitis C Incidence

panel A - Random effects meta-analysis of Hepatitis C incidence

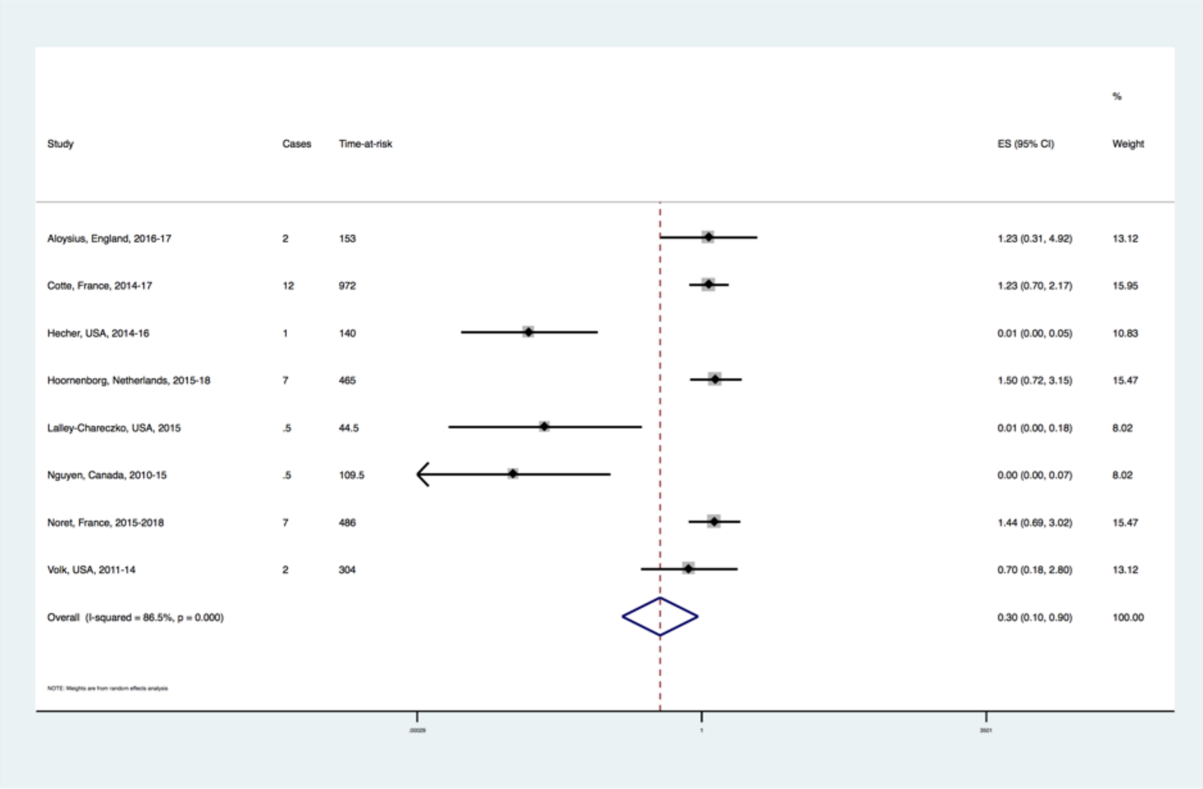

© 2019 Ong JJ et al. *JAMA Network Open.*

eFigure 11, panel B - Random effects meta-analysis of Hepatitis C incidence by MSM status of the study population

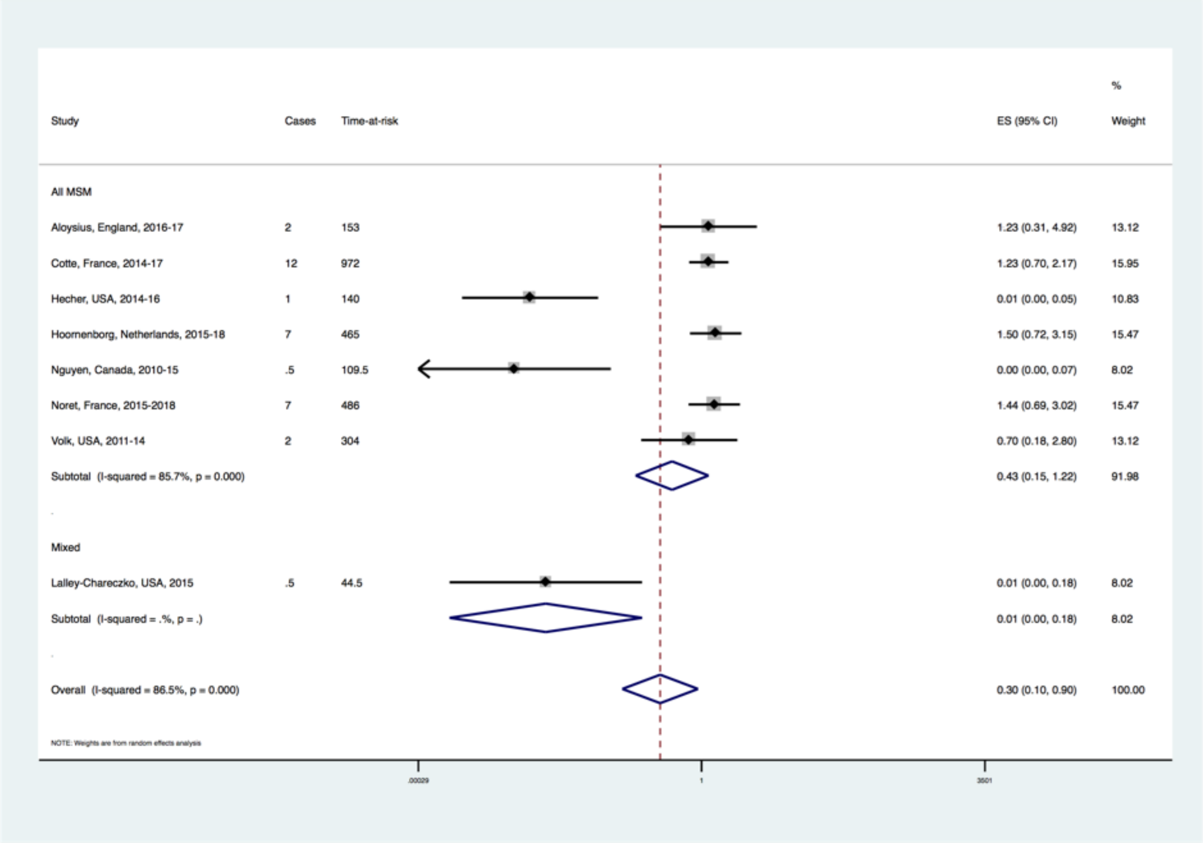

© 2019 Ong JJ et al. *JAMA Network Open.*

eFigure 11, panel C - Random effects meta-analysis of Hepatitis C incidence by country income level

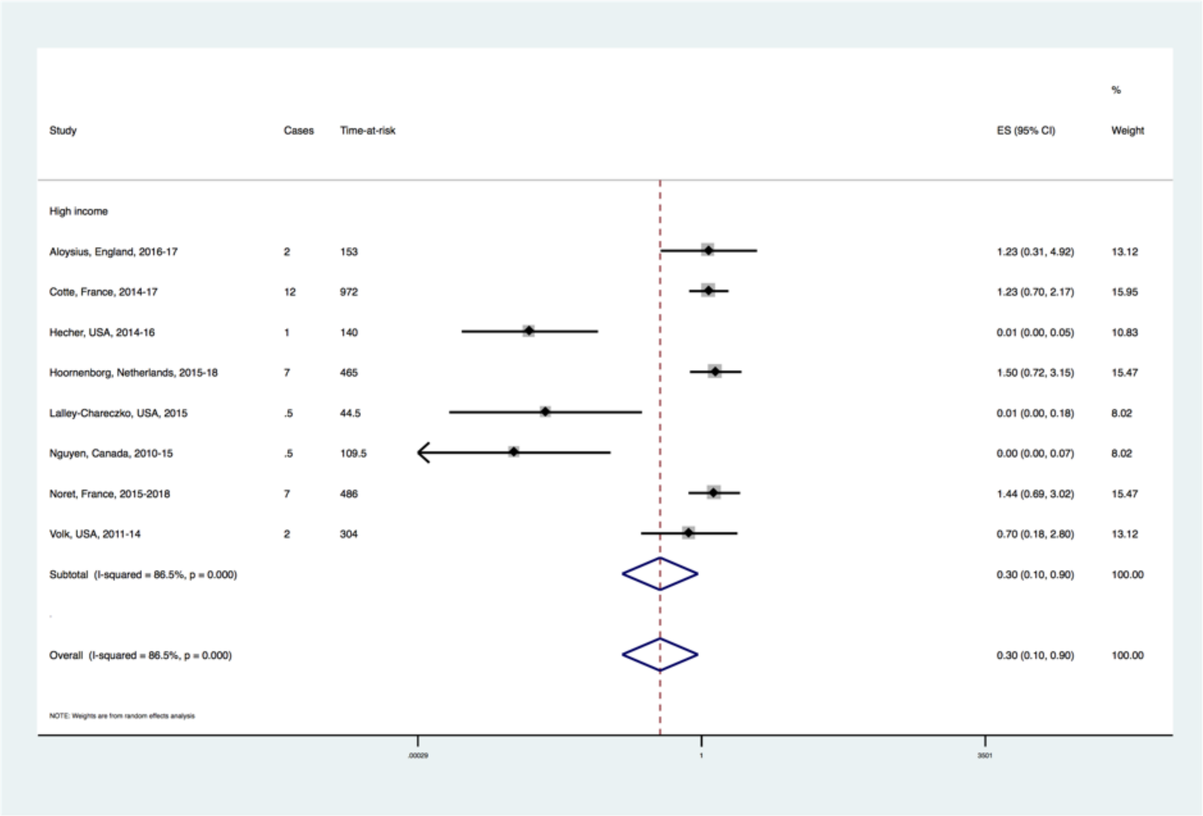

© 2019 Ong JJ et al. *JAMA Network Open.*

eFigure 11, panel D Funnel plot and Egger's test for Hepatitis C incidence

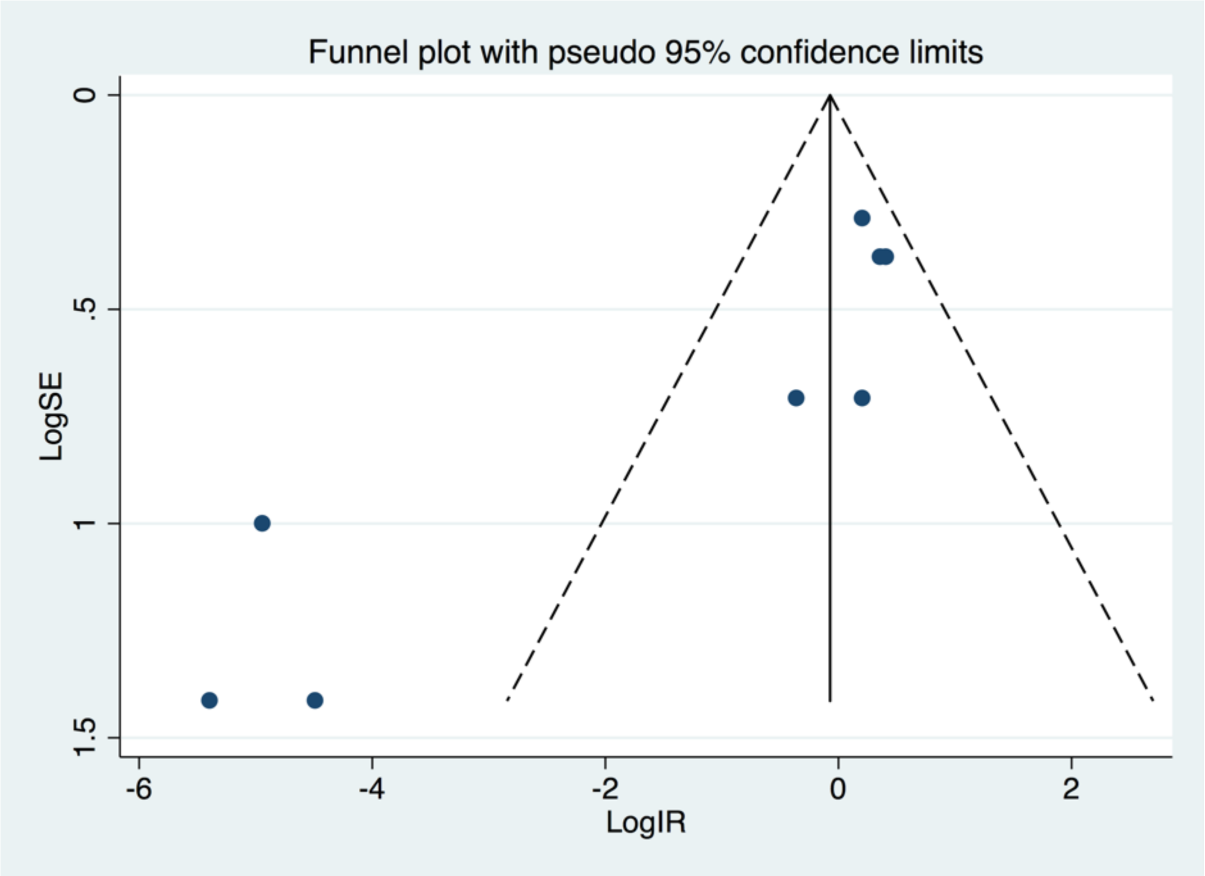

Egger's Test = 0.008

© 2019 Ong JJ et al. *JAMA Network Open.*
